# Global burden and genetic insights of RA and JIA in ages 0–19 years: GBD 2021 and MR analysis

**DOI:** 10.3389/fimmu.2025.1661461

**Published:** 2026-01-14

**Authors:** Pingyu An, Zhiqiang Ma, Tian Tian, Shibo Zhang, Jian Wang, Zhiliang Tian

**Affiliations:** 1 Department of Pediatrics, The Second Affiliated Hospital of Harbin Medical University, Harbin, China; 2 Department of Dermatology, Leiden University Medical Center, Leiden, Netherlands; 3 Department of Neurosurgery, The Second Affiliated Hospital of Harbin Medical University, Harbin, China; 4 Department of Pediatrics, The Sixth Affiliated Hospital of Harbin Medical University, Harbin, China

**Keywords:** epidemiology, global burden of disease, juvenile idiopathic arthritis, Mendelian randomization, rheumatoid arthritis

## Abstract

**Background:**

Rheumatoid arthritis (RA) is a chronic autoimmune arthritis that predominantly affects adults, whereas juvenile idiopathic arthritis (JIA) comprises a heterogeneous group of childhood−onset arthritides defined by age at onset, clinical phenotype, and immunological profile. Although RA and JIA are classified as distinct diseases, they share overlapping clinical and immunological features, and the nosological relationship between RA and some JIA subtypes remains debated. The global burden of RA and JIA in young people, and the similarities and differences in their underlying immunogenetic features, remain incompletely understood.

**Methods:**

We utilized GBD 2021 data to describe trends in incidence, prevalence, and disability-adjusted life years for RA in individuals aged 0–19 years, as defined within the GBD framework, from 1990 to 2021. In parallel, we performed two−sample Mendelian randomization (MR) using genome−wide association study summary statistics from European−ancestry cohorts to assess associations of genetically proxied levels of 731 immune−cell traits, 91 circulating inflammatory proteins, and 1,400 serum metabolites with RA and JIA risk.

**Results:**

From 1990 to 2021, global age−standardized incidence and prevalence of RA in individuals aged 0–19 years increased, whereas disability−adjusted life years declined slightly. The aggregated burden was higher in females and in high−sociodemographic index regions. MR identified overlapping genetically proxied immune−cell and inflammatory protein traits for RA and JIA, including CD28 on CD8^+^CD45RA^+^ T cells, CD25 on memory B cells, signaling lymphocytic activation molecule, and Fms−like tyrosine kinase 3 ligand, whereas higher genetically predicted levels of activated regulatory T cells and HLA−DR on dendritic cells were associated with lower risk in both diseases.

**Conclusion:**

Our study highlights a rising global burden of GBD−defined RA among children and adolescents and delineates shared and distinct patterns of genetically predicted immune−cell, inflammatory protein, and metabolite traits associated with RA and JIA. These observations suggest that immunologically informed approaches could complement existing age- and phenotype-based classifications and help refine early recognition and risk stratification of inflammatory arthritis across the life course.

## Graphical Abstract

**Figure:**
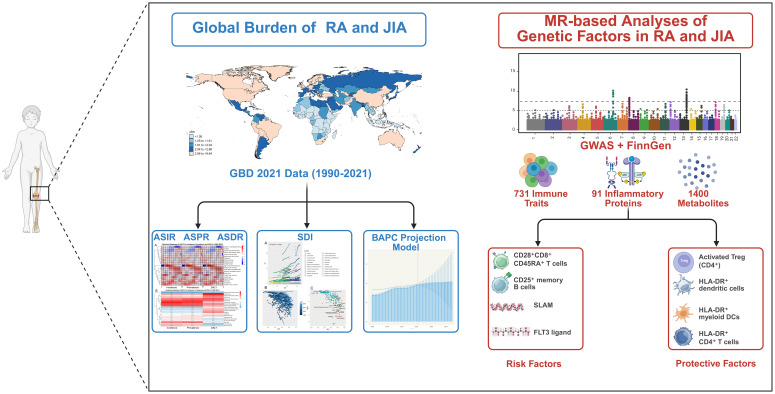
Study overview integrating GBD burden estimation and two‑sample Mendelian randomization. The left panel shows the epidemiological component based on Global Burden of Disease (GBD) 2021 estimates (1990–2021), used to quantify incidence, prevalence, and disability‑adjusted life years (DALYs) for GBD‑defined RA in individuals aged 0–19 years (in the GBD framework, RA and JIA ICD‑10 codes are modelled jointly). We further examined socio-demographic index (SDI), inequality/frontier patterns, and projected future trends using a Bayesian age–period–cohort (BAPC) model. The right panel shows the genetic component: two‑sample Mendelian randomization (MR) using summary statistics from large GWAS and FinnGen evaluated associations between genetically proxied levels of 731 immune‑cell traits, 91 circulating inflammatory proteins, and 1,400 serum metabolites and the risk of RA and JIA. Boxes highlight representative traits showing positive or inverse associations with disease risk. RA, rheumatoid arthritis; JIA, juvenile idiopathic arthritis; GBD, Global Burden of Disease; MR, Mendelian randomization; GWAS, genome‑wide association study; DALY, disability‑adjusted life year; ASIR/ASPR/ASDR, age‑standardized incidence/prevalence/DALY rate; SDI, socio-demographic index; BAPC, Bayesian age–period–cohort; DC, dendritic cell; Treg, regulatory T cell.

## 1 Introduction

Rheumatoid arthritis (RA) is a chronic, disabling autoimmune disease characterized by persistent synovitis and progressive joint destruction, and affects approximately 0.2–1% of the global population, predominantly in adults (1, 2). Juvenile idiopathic arthritis (JIA), defined as arthritis of unknown cause persisting for at least six weeks with onset before 16 years of age, is the most common chronic inflammatory rheumatic disease in childhood (3). Based on clinical and laboratory features, the International League of Associations for Rheumatology (ILAR) classifies JIA into seven subtypes (3, 4). Current classification criteria treat RA and JIA as separate disease entities, but they share overlapping clinical and immunological features, and the nosological relationship between RA and some JIA subtypes remains a matter of debate. In particular, rheumatoid factor (RF)–positive polyarticular JIA closely resembles adult−onset RA in clinical course and, at least partly, in underlying pathogenesis, whereas other JIA subtypes such as oligoarticular, systemic, enthesitis−related, and psoriatic arthritis have distinct clinical and genetic profiles that do not correspond to RA (4, 5). A proportion of children with JIA transition to adult rheumatic diseases, yet the mechanisms underlying these trajectories remain incompletely understood. In both RA and JIA, complex interactions between genetic susceptibility, dysregulated innate and adaptive immune responses, and environmental exposures contribute to disease onset and progression; however, precise pathogenic pathways and robust biomarkers that distinguish these conditions are still lacking (5).

Despite their substantial clinical and societal impact, contemporary data on the global burden of RA and JIA in children and adolescents remain limited. The Global Burden of Disease (GBD) study provides harmonized estimates of incidence, prevalence, and disability−adjusted life years (DALYs) for RA, but in individuals aged 0–19 years the RA category aggregates ICD−10 codes M05–M06.9 and M08.0–M08.8, thereby including some JIA cases under the RA label (6). In this framework, RA and JIA cannot be separated at the population level. In this epidemiological analysis, the term “RA in ages 0–19 years” is used to denote this aggregated GBD category, while explicitly acknowledging that RA and JIA remain nosologically distinct. From an immunological perspective, it is necessary to clarify the extent to which RA and JIA share causal immune pathways across the age spectrum. Mendelian randomization (MR) using genome−wide association study (GWAS) data offers an opportunity to assess the effects of genetically proxied immune−cell traits, circulating inflammatory proteins, and metabolites on disease risk, thereby complementing observational and experimental findings. In this study, we first use GBD 2021 estimates to characterize global, regional, and national trends in the burden of RA among individuals aged 0–19 years from 1990 to 2021 and to project this burden to 2044. We then perform two−sample MR analyses to compare the associations of 731 immune−cell traits, 91 inflammatory proteins, and 1,400 blood metabolites with RA and JIA. By integrating global burden estimates with genetically informed immune traits, we aim to provide a more comprehensive view of shared and distinct pathways in RA and JIA and to support the development of mechanism−based diagnostic and preventive strategies that move beyond purely age−based classifications.

## 2 Methods

### 2.1 Data sources

The global RA burden data used in this study were obtained from the GBD 2021 database, which covers 371 diseases, injuries, and impairments across 204 countries and territories, along with 88 risk factors. Under the coordination of the Institute for Health Metrics and Evaluation (IHME), GBD integrates data from population surveys, hospital records, clinical registries, and systematic reviews to ensure statistical accuracy and consistency (6, 7). Detailed methodologies for data collection and analysis have been previously described in GBD publications.

In GBD 2021, estimates of RA in children and adolescents are based on ICD−10 codes M05-M06.9 and M08.0-M08.8, thereby aggregating RA and JIA−coded arthritis cases without distinguishing ILAR JIA subtypes. Accordingly, in the present analyses we use the term “RA in ages 0–19 years” to denote this aggregated GBD category, while acknowledging that RA and JIA are nosologically distinct diseases. According to the World Health Organization, children and adolescents are defined as individuals aged 0 to 19 years. We extracted annual data on RA prevalence, incidence, and DALYs for this age group from 1990 to 2021. Individuals were stratified into four age groups: early childhood (<5 years), middle childhood (5–9 years), early adolescence (10–14 years), and late adolescence (15–19 years).

Age−standardized prevalence rates, incidence rates, and DALY rates were estimated using a Bayesian hierarchical model to adjust for differences in age structure and facilitate comparability of disease burden across populations. Covariates including age, sex, location, and socio-demographic index (SDI) were incorporated into the modeling framework. SDI is a composite measure of income, education, and fertility rates, scaled from 0 (lowest income, least education, highest fertility) to 1 (highest income, most education, lowest fertility). Data quality was assessed using standard GBD metrics, with uncertainty intervals (UIs) calculated as the 2.5th and 97.5th percentiles of posterior distributions derived from 1,000 Monte Carlo simulations. All estimates are reported with corresponding 95% UIs to reflect variability and reliability.

For epidemiological analyses, data processing was conducted using R software (version 4.4.0) and Python (version 3.9). As this study utilized publicly available secondary data without individual−level identifiers, no additional ethical approval was required. The University of Washington Institutional Review Board reviewed and approved a waiver of informed consent for the GBD study. This study was reported following the Strengthening the Reporting of Observational Studies in Epidemiology (STROBE) guidelines (8).

For the MR analyses, genetic instruments for the exposure traits were obtained from publicly available GWAS. Data on immune−cell traits (n = 731) were sourced from a GWAS investigating the genetic architecture of immune cells (9). Circulating inflammatory protein data (n = 91) were derived from a GWAS conducted among 14,824 individuals of European ancestry. Blood metabolite data (n = 1,091 metabolites and 309 metabolite ratios) were obtained from a comprehensive GWAS based on the Canadian Longitudinal Study on Aging (CLSA) cohort comprising 8,299 individuals (10, 11). Summary statistics for RA outcomes were obtained from the FinnGen consortium database (Release 12, M13_RHEUMA), and those for JIA were sourced from a GWAS conducted by Hinks and colleagues (12, 13). All GWAS participants were predominantly of European descent.

All GWAS summary statistics used in the MR analyses were obtained from the FinnGen consortium and the OpenGWAS public database. Data were de−identified before release and did not involve any personal or identifiable information. Consequently, no additional institutional ethical approval or informed consent was required for the MR component of this study.

### 2.2 Statistical analysis

To evaluate trends in the ASRs of prevalence, incidence, and DALYs, we used the estimated annual percentage change (EAPC) method. ASRs are expressed per 100,000 population, calculated using the formula:


$$ASR\;=\;\frac{\begin{array}{l} \left(\Sigma_{\text{i}=1}^{\text{A}}w_{i}\;r_{i}\right) \end{array}}{\left(\Sigma_{\text{i}=1}^{\text{A}}w_{i}\right)}\;\times \;100,000$$

(r_i_: the age-specific rate in the $i^{th}$ age group; w_i_: the number of individuals in the $i^{th}$ age group of the standard population; A: the total number of age groups).

The EAPC was used to assess temporal trends by fitting a regression model with the natural logarithm of the age-standardized rate (ln[ASR]) as the dependent variable and calendar year (X) as the independent variable, following the model: ln(ASR) = α + βX + e, where α represents the intercept, β the trend coefficient, and e the error term. The EAPC was calculated using the formula 100 × [exp(β) – 1], describing the overall trend across the period. The 95% confidence intervals (CIs) for EAPCs were computed to evaluate the significance of trends: trends were defined as increasing if both the EAPC estimate and the lower limit of the CI were positive, decreasing if both the EAPC estimate and the upper limit of the CI were negative, and stable otherwise. Gaussian process regression and Pearson’s correlation coefficient were used to assess the associations between EAPCs, ASRs, and SDI. The average annual percent change (AAPC) was derived by averaging the annual percentage change (APC) values across all years, allowing comparisons across multiple periods. Z-score standardization of AAPCs and EAPCs was performed before hierarchical clustering to facilitate multidimensional comparisons across regions and timeframes during visualization.

### 2.3 Prediction analysis

The Bayesian Age-Period-Cohort (BAPC) model was used to project the incidence and burden of rheumatoid arthritis among individuals aged 0–19 years from 2022 to 2044. The BAPC model operates within a Bayesian framework, incorporating prior information and generating credible intervals to capture uncertainty while simultaneously quantifying the effects of age, period, and cohort.

### 2.4 Frontier analysis

Data Envelopment Analysis (DEA) was employed as the core method for frontier analysis, using the Free Disposal Hull (FDH) approach to accommodate non-linear data structures. Frontier analysis was used to assess the relative potential of countries or regions to reduce the burden of rheumatoid arthritis. The linear relationship between the SDI and the annual age-standardized DALY rate in 2021 was quantified using Spearman’s rank correlation coefficient, ranging from -1 to 1, with values closer to 1 or -1 indicating strong positive or negative correlations, respectively.

### 2.5 Global health inequality analysis

To quantify global health inequality in the burden of RA, we used two established metrics: the slope index of inequality (SII) and the concentration index. The SII was derived from regression analysis across populations ranked by socioeconomic status, reflecting differences in health outcomes between the lowest and highest groups. A positive SII indicates a higher burden among higher socioeconomic groups, whereas a negative SII indicates a higher burden among lower socioeconomic groups. The concentration index, ranging from -1 to 1, assessed the relative inequality in the distribution of rheumatoid arthritis burden, with positive values indicating concentration in higher socioeconomic groups, negative values indicating concentration in lower groups, and a value of 0 representing perfect equality. The concentration index was calculated using the Lorenz curve, quantifying inequality as the area between the Lorenz curve and the line of equality. All analyses followed established guidelines for inequality measurement in global health research to ensure robustness and comparability across populations and regions. This analysis highlighted persistent global health disparities and identified vulnerable populations who may benefit from targeted interventions.

### 2.6 Mendelian randomization analysis

We performed two−sample MR analyses to explore the potential causal effects of genetically proxied levels of immune−cell traits, circulating inflammatory proteins, and blood metabolites on the risk of RA and JIA. For each exposure, single nucleotide polymorphisms (SNPs) associated at a suggestive genome-wide significance threshold (P < 1×10^−5^) were selected as instrumental variables. Independent SNPs were retained by clumping at r² < 0.1 within a 500-kb window using a European ancestry reference panel. Harmonization was conducted to align the effect alleles between exposure and outcome datasets, with ambiguous palindromic SNPs removed. The inverse-variance weighted (IVW) method was used as the primary analysis, while sensitivity analyses included MR-Egger regression, weighted median, weighted mode, and simple mode approaches. Heterogeneity was assessed using Cochran’s Q statistic, and horizontal pleiotropy was evaluated via the MR-Egger intercept and the MR-PRESSO global test. Steiger filtering was applied to confirm the directionality of causation from exposure to outcome. All analyses were conducted in R (version 4.4.0) using the TwoSampleMR (v0.5.6), Mendelian Randomization, mr.raps, and ieugwasr packages.

## 3 Results

### 3.1 Temporal trends and spatial distribution of rheumatoid arthritis in individuals aged 0–19 years

**Figure 1:**
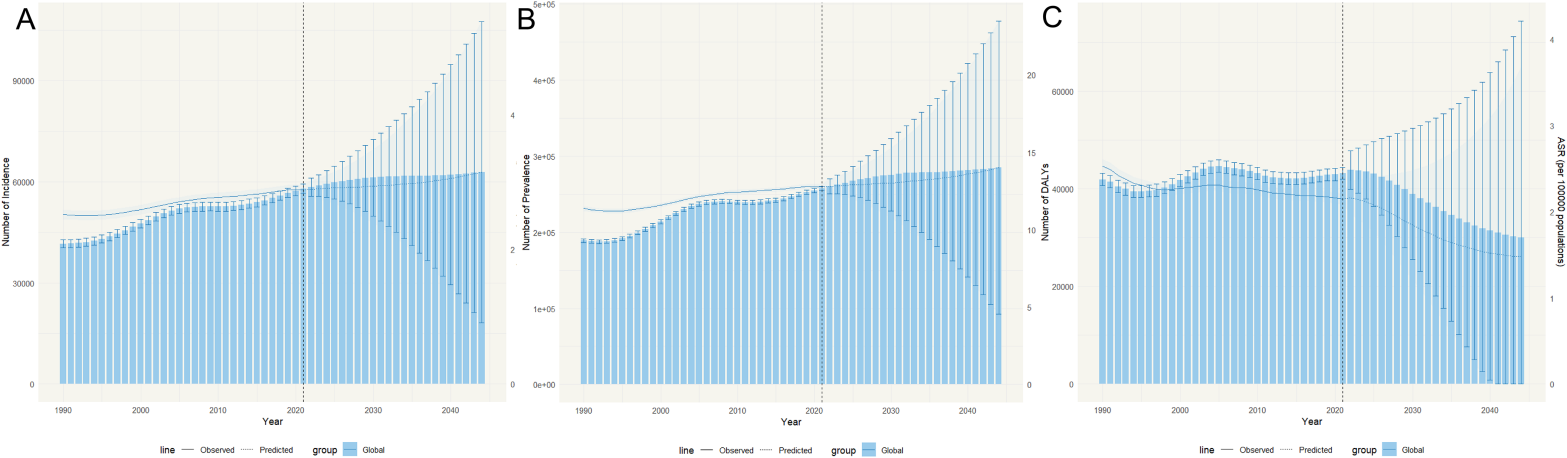
Predicted trends in incidence, prevalence, DALY numbers, and age-standardized rates (per 100,000 population) from 2022 to 2044, based on BAPC. The bar chart illustrates changes in numbers, while the curve depicts changes in age-standardized rates. **(A)** Predictions of RA incidence numbers and age-standardized incidence rates using the BAPC model from 2022 to 2044; **(B)** Predictions of RA prevalence numbers and age-standardized prevalence rates using the BAPC model from 2022 to 2044. **(C)** Predictions of RA DALY numbers and age-standardized DALY rates using the BAPC model from 2022 to 2044. DALY, disability-adjusted life year; BAPC, Bayesian Age-Period-Cohort; RA, rheumatoid arthritis.

From 1990 to 2021, global incident cases of RA as defined in the GBD study (ICD−10 M05-M06.9 and M08.0-M08.8) among individuals aged 0–19 years increased by 38.88%, from 41,651.7 to 57,850.9, with consistently higher numbers in females. The age-standardized incidence rate (ASIR) rose from 1.8 to 2.1 per 100,000 (EAPC 0.54%; 95% CI 0.50–0.57), and the age-standardized prevalence rate (ASPR) increased from 8.3 to 9.3 per 100,000 (EAPC 0.51%; 95% CI 0.46–0.55). While total DALYs rose modestly (3.07%), the age-standardized DALY rate (ASDR) declined from 1.8 to 1.6 per 100,000 (EAPC –0.35%; 95% CI –0.42 to –0.28) (**Table 1**). Bayesian age–period–cohort projections suggest that incidence and prevalence will continue to rise through 2044, whereas DALYs and ASDR will gradually decline after a peak in 2022 (**Figure 1**). In 2021, middle SDI regions had the highest absolute burden, whereas high SDI regions reported the highest ASIR, ASPR, and ASDR with the fastest growth. Detailed national and regional data are presented in **Table 1** and **Supplementary 1** (**Supplementary Figures S1**, **S2**; **Supplementary Table S1**). Overall, national trends aligned with regional patterns.

**Table 1:** Incidence, prevalence, DALYs, and age-standardized rates in 2021, and EAPC of ASRs from 1990 globally and across GBD regions.

Location	Incidence			Prevalence			DALYs		
	Case number (95%UI)	ASR (per 100,000) (95%UI)	1990–2021 EAPC (95% CI)	Case number (95%UI)	ASR (per 100,000) (95%UI)	1990–2021 EAPC (95% CI)	Case number (95%UI)	ASR (per 100,000) (95%UI)	1990–2021 EAPC (95% CI)
Global									
Global	57850.9 (35193.5, 85991.5)	2.1 (1.3, 3.1)	0.54 (0.5, 0.57)	257574.8 (167806.8, 367524.2)	9.3 (6.1, 13.3)	0.51 (0.46, 0.55)	43219.4 (26591.3, 67980.4)	1.6 (1, 2.5)	-0.35 (-0.42, -0.28)
Male	17034.5 (9939, 26394.6)	1.2 (0.7, 1.9)	0.44 (0.41, 0.47)	81279.2 (49252.7, 119153.6)	5.7 (3.5, 8.4)	0.38 (0.34, 0.42)	14319.8 (8783.4, 22673.1)	1 (0.6, 1.6)	-0.55 (-0.62, -0.48)
Female	40816 (25419, 60023)	3 (1.9, 4.5)	0.6 (0.56, 0.64)	176295.6 (116721.6, 248459.3)	13.1 (8.7, 18.5)	0.59 (0.54, 0.64)	28899.6 (17962.4, 44716.8)	2.2 (1.3, 3.3)	-0.23 (-0.3, -0.15)
SDI									
High SDI	8429.6 (5978.7, 11441.7)	3.4 (2.4, 4.5)	0.96 (0.86, 1.06)	48141 (36928.7, 61053.2)	18.6 (14.2, 23.6)	1.11 (0.99, 1.24)	7361.8 (4767.5, 11125)	2.9 (1.8, 4.3)	0.49 (0.36, 0.62)
High-middle SDI	7964.8 (4742, 12088.1)	2.5 (1.5, 3.8)	0.93 (0.9, 0.96)	34352.6 (21544.6, 49804.5)	10.6 (6.7, 15.4)	0.91 (0.87, 0.94)	6057.7 (3750, 9490.4)	1.9 (1.2, 2.9)	-0.19 (-0.26, -0.12)
Middle SDI	19694.3 (11777.3, 29786.8)	2.5 (1.5, 3.7)	0.77 (0.75, 0.8)	82159.5 (51756.3, 120115.8)	10.2 (6.4, 14.9)	0.71 (0.68, 0.73)	14555.5 (9156.9, 22563.9)	1.8 (1.1, 2.8)	-0.43 (-0.52, -0.34)
Low-middle SDI	15418.9 (9123.3, 23085.9)	1.9 (1.1, 2.9)	0.79 (0.74, 0.83)	65066.5 (40142.9, 94997.1)	8 (4.9, 11.7)	0.71 (0.67, 0.75)	10654.4 (6157.3, 17086.5)	1.3 (0.8, 2.1)	0.33 (0.31, 0.36)
Low SDI	6310.3 (3490.6, 9992.1)	1.1 (0.6, 1.8)	0.47 (0.4, 0.55)	27705.8 (15780.5, 42534)	4.9 (2.8, 7.6)	0.43 (0.37, 0.49)	4561.5 (2403.9, 7594.8)	0.8 (0.4, 1.3)	0.28 (0.24, 0.33)
GBD regions									
High-income Asia Pacific	780.7 (460.9, 1180.1)	2.2 (1.3, 3.3)	0.69 (0.66, 0.71)	3457.2 (2163.8, 5024.4)	9.6 (6, 14)	0.65 (0.62, 0.69)	575.3 (341.3, 922.5)	1.6 (1, 2.6)	-0.55 (-0.72, -0.39)
High-income North America	3787.9 (3015.5, 4773.4)	4 (3.2, 5)	0.98 (0.75, 1.21)	25814.2 (21377.1, 31228.6)	25.5 (21.1, 30.9)	0.99 (0.73, 1.25)	3841.6 (2547.5, 5582.2)	3.8 (2.5, 5.5)	0.78 (0.55, 1.01)
Western Europe	3355.5 (2104.6, 4885.3)	3.4 (2.1, 4.9)	0.6 (0.55, 0.65)	17099.9 (11321.6, 23560.3)	16.7 (11, 23.1)	0.67 (0.61, 0.73)	2647.5 (1595, 4224.1)	2.6 (1.6, 4.1)	0.15 (0.12, 0.19)
Australasia	225 (128.6, 352.7)	2.8 (1.6, 4.4)	1.12 (1.05, 1.19)	1049.3 (645.6, 1508)	13 (8, 18.7)	1.1 (1.03, 1.17)	159.8 (81.3, 269.7)	2 (1, 3.4)	0.67 (0.62, 0.71)
Andean Latin America	1666.3 (1086.9, 2386.7)	6.8 (4.4, 9.7)	1.26 (1.2, 1.32)	6480.8 (4494.3, 8876)	26.3 (18.2, 36)	1.13 (1.09, 1.18)	1008.6 (578.5, 1603.5)	4.1 (2.3, 6.5)	0.16 (0.06, 0.26)
Tropical Latin America	2830.5 (1811.6, 4079.3)	4 (2.6, 5.8)	0.46 (0.39, 0.53)	11790.9 (7793.7, 16421.6)	16.5 (10.9, 23.1)	0.44 (0.37, 0.51)	2233.4 (1472.3, 3250.6)	3.2 (2.1, 4.6)	0.29 (0.15, 0.44)
Central Latin America	2286.6 (1297.9, 3667)	2.4 (1.4, 3.9)	0.71 (0.55, 0.86)	9266.8 (5608, 14161.9)	9.7 (5.9, 14.9)	0.63 (0.49, 0.77)	1822.2 (1179.1, 2765.3)	1.9 (1.3, 2.9)	-0.48 (-0.58, -0.39)
Southern Latin America	628 (357.4, 988.2)	2.9 (1.7, 4.6)	1.11 (1.02, 1.2)	2985.9 (1836.8, 4345.6)	13.7 (8.4, 19.9)	1.04 (0.95, 1.13)	526.4 (309.4, 851.3)	2.4 (1.4, 3.9)	0.3 (0.14, 0.47)
Caribbean	266.5 (150.3, 416.7)	1.6 (0.9, 2.6)	0.63 (0.58, 0.67)	1171.5 (704.4, 1751.8)	7.1 (4.3, 10.7)	0.58 (0.53, 0.62)	295.5 (179.5, 462.3)	1.8 (1.1, 2.9)	-0.05 (-0.1, 0.01)
Central Europe	541.6 (311.6, 835.7)	2.1 (1.2, 3.3)	0.79 (0.76, 0.83)	2332.8 (1418.1, 3439.7)	9 (5.5, 13.3)	0.72 (0.68, 0.76)	386.1 (227.9, 626.5)	1.5 (0.9, 2.4)	-1.03 (-1.2, -0.86)
Eastern Europe	921 (509.4, 1486.4)	1.9 (1, 3)	0.84 (0.78, 0.9)	3727.2 (2204, 5756.7)	7.7 (4.5, 11.8)	0.75 (0.68, 0.81)	930.4 (668.6, 1320.3)	1.9 (1.4, 2.7)	-0.81 (-0.99, -0.62)
Central Asia	713.5 (412.2, 1123.6)	2.2 (1.3, 3.5)	0.9 (0.86, 0.94)	2843.3 (1732.3, 4261.3)	8.9 (5.5, 13.4)	0.81 (0.77, 0.85)	476.6 (278.8, 759.5)	1.5 (0.9, 2.4)	1.24 (1.01, 1.48)
North Africa and Middle East	5159.7 (3175.1, 7628.7)	2.2 (1.3, 3.2)	1.18 (1.11, 1.25)	21947.6 (13943.3, 31204.3)	9.2 (5.8, 13.1)	1.06 (1, 1.12)	4155.4 (2607.4, 6244.6)	1.7 (1.1, 2.6)	0.48 (0.37, 0.58)
South Asia	16373.6 (9704.7, 24515.3)	2.2 (1.3, 3.2)	0.84 (0.74, 0.93)	67764.6 (42242.9, 99510.8)	8.8 (5.5, 13)	0.76 (0.67, 0.84)	10253.7 (5719.7, 16736.2)	1.3 (0.7, 2.2)	0.51 (0.45, 0.57)
Southeast Asia	2634.8 (1446.3, 4212.4)	1.1 (0.6, 1.7)	0.71 (0.67, 0.75)	12844.1 (7410.3, 19372)	5.2 (3, 7.9)	0.65 (0.59, 0.71)	2426.2 (1456.6, 3851)	1 (0.6, 1.6)	-0.27 (-0.3, -0.24)
East Asia	10016.3 (5916.6, 15197)	2.8 (1.7, 4.3)	1.01 (0.98, 1.05)	41908.1 (26163, 61703.4)	11.9 (7.4, 17.5)	0.99 (0.96, 1.02)	7552.7 (4714.8, 11781.1)	2.1 (1.3, 3.3)	-0.36 (-0.51, -0.22)
Oceania	67.3 (37.7, 105.8)	1.1 (0.6, 1.8)	0.44 (0.4, 0.47)	324.3 (190.4, 489)	5.5 (3.2, 8.2)	0.42 (0.4, 0.45)	48.6 (25.1, 84.1)	0.8 (0.4, 1.4)	0.47 (0.45, 0.48)
Western Sub-Saharan Africa	2345.2 (1264.5, 3779.2)	0.9 (0.5, 1.5)	0.71 (0.64, 0.78)	10526.8 (5797.7, 16318.1)	4.3 (2.4, 6.6)	0.64 (0.59, 0.7)	1544.9 (758.8, 2711.7)	0.6 (0.3, 1.1)	0.63 (0.58, 0.69)
Eastern Sub-Saharan Africa	1805 (895.9, 3086)	0.8 (0.4, 1.4)	0.4 (0.36, 0.44)	8240.9 (4326.4, 13324.7)	3.7 (2, 6)	0.35 (0.32, 0.38)	1262.3 (597, 2324.5)	0.6 (0.3, 1)	0.13 (0.09, 0.16)
Central Sub-Saharan Africa	623.7 (313.6, 1055.4)	0.9 (0.5, 1.5)	0.26 (0.22, 0.3)	2803.5 (1499.2, 4461.8)	4.1 (2.2, 6.5)	0.19 (0.16, 0.22)	431.6 (203, 842)	0.6 (0.3, 1.2)	-0.02 (-0.06, 0.01)
Southern Sub-Saharan Africa	822.1 (469.7, 1308.6)	2.6 (1.5, 4.1)	-0.1 (-0.21, 0.02)	3195.2 (1932, 4845.6)	9.9 (6, 15.1)	-0.07 (-0.18, 0.04)	640.7 (406.5, 993.8)	2 (1.3, 3.1)	-1.26 (-1.52, -1)

The highest values in each column are highlighted in red and the lowest values in blue. DALYs, disability-adjusted life years; EAPC, estimated annual percentage change.

### 3.2 Age and regional patterns of rheumatoid arthritis burden among children and adolescents

From 1990 to 2021, the burden of GBD−defined RA among individuals aged 0–19 years was consistently concentrated in the 10–19-year group, with a rising proportion among adolescents aged 15–19 years (**Supplementary Figure S3** in **Supplementary 1**). Middle SDI region accounted for the largest share of new and prevalent cases, while incidence and prevalence rates were highest in Andean Latin America, particularly among those aged 15–19 years (**Supplementary Figures S4**, **S5** in **Supplementary 1**). Global incident and prevalent cases steadily increased, with the sharpest rise in adolescents. DALYs also rose, with Andean Latin America maintaining the highest DALY rates throughout the period (**Supplementary Figure S6** in **Supplementary 1**).

### 3.3 Sex differences in the burden of rheumatoid arthritis among children and adolescents

From 1990 to 2021, the number of incident RA cases among individuals aged 5–19 years increased globally, with females consistently bearing a higher burden across all age groups. The rise in incidence was more pronounced among females, suggesting a widening sex disparity over time. Analysis of ASIR trends showed that females had higher rates than males in both 1990 and 2021 (**Supplementary Figure S7** in **Supplementary 1**). In 2021, female ASIR continued to rise in several regions, whereas male ASIR remained relatively stable. These findings highlight significant sex and regional heterogeneity in RA burden, likely driven by genetic, hormonal, and environmental factors.

### 3.4 Association between RA burden and SDI and frontier analysis

Globally, the ASDR of GBD−defined RA showed a weak positive correlation with SDI (Spearman’s R = 0.20; P < 0.001), with higher burdens reported in higher-SDI regions. Somalia, Eritrea, Chad, Madagascar, Niger, Burundi, Ethiopia, Mali, Djibouti, and Guinea-Bissau had the smallest effective differences and ASDRs (range, 0.45–0.54). In contrast, Bolivia, the Netherlands, Ireland, Brazil, Finland, Kuwait, the United States, the United Kingdom, Paraguay, and Peru had the largest effective differences and ASDRs, suggesting greater deviations from expected burden levels based on their socio-economic development (**Supplementary Figure S8** in **Supplementary 1**, **Supplementary Table S2** in **Supplementary 1**). These patterns were consistent with overall global and regional trends.

### 3.5 Health inequality in the burden of rheumatoid arthritis among children and adolescents

In both 1990 and 2021, the concentration curves for DALYs due to GBD−defined RA lay below the equality line, indicating a higher burden in countries with higher SDI levels. The concentration index remained stable over time (0.21 [95% CI, 0.18–0.25] in 1990 and 0.21 [95% CI, 0.18–0.24] in 2021), suggesting persistent socio-economic disparities. Regression analysis further showed that RA ASDR increased with SDI in both years, with identical regression slopes (both equal to 1), indicating no significant change in inequality over the study period (**Supplementary Figure S9** in **Supplementary 1**). The United States, China, and India played major roles in shaping the global distribution of disease burden across different development levels.

### 3.6 Two-sample Mendelian randomization analysis

**Figure 2:**
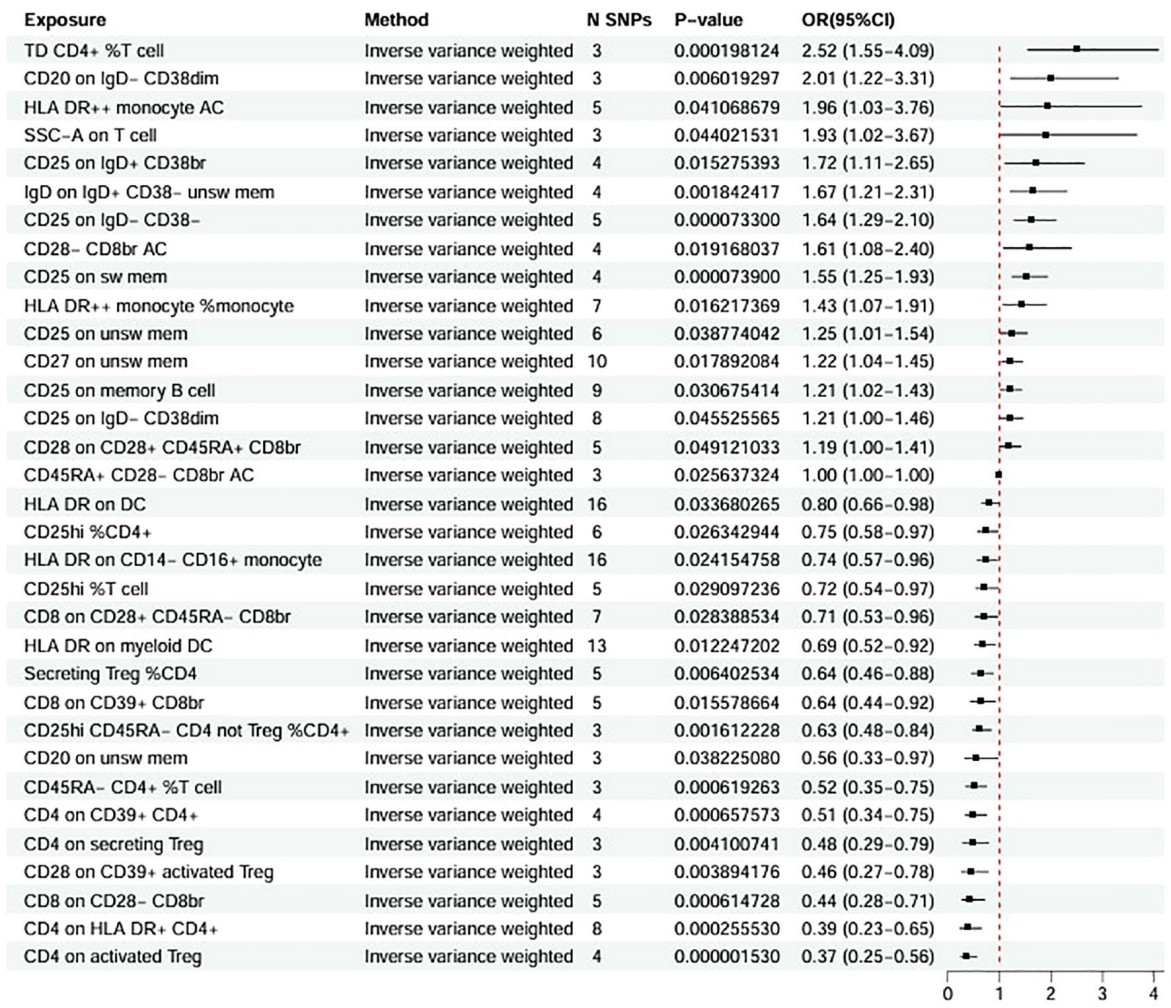
Mendelian randomization estimates of the causal associations between immune cells and JIA. The forest plot shows the statistically significant results of IVW Mendelian randomization analyses evaluating the associations between 731 immune cells and the risk of JIA. Each row represents a specific immune cell, with ORs and 95% CIs plotted. ORs greater than 1 indicate increased risk, and ORs less than 1 indicate a protective effect. JIA, juvenile idiopathic arthritis; IVW, inverse-variance weighted; OR, odds ratio; CI, confidence intervals.

Using the inverse-variance weighted method in two-sample MR, genetically proxied levels of 120 immune-cell traits, 19 circulating inflammatory proteins, and 10 blood metabolites were associated with RA risk at P < 0.05, and 33, 6, and 6 traits, respectively, with JIA risk. In RA, genetically predicted higher levels of CD4^+^ activated cells showed the strongest positive association with risk (OR 1.11; 95% CI 1.04–1.18), whereas higher levels of HLA−DR^++^ monocytes were most strongly inversely associated with risk (OR 0.82; 95% CI 0.75–0.89) (**Supplementary Figure S10** in **Supplementary 1**). In JIA, higher proportions of TD CD4^+^ %T cells showed the largest positive association with disease risk (OR 2.52; 95% CI 1.55–4.09), while higher CD4 expression on activated regulatory T cells was associated with lower risk (OR 0.37; 95% CI 0.25–0.56) (**Figure 2**).

For circulating proteins, genetically proxied interferon−γ levels were associated with increased RA risk (OR 1.13; 95% CI 1.04–1.23), whereas fibroblast growth factor 19 levels were inversely associated with risk (OR 0.92; 95% CI 0.87–0.97) (**Supplementary Figure S11** in **Supplementary 1**). In JIA, CXCL9 showed the strongest positive association with disease risk (OR 10.97; 95% CI 5.23–23.05), whereas levels of lymphotoxin−α (historically termed TNF−β in the source proteomic GWAS) were inversely associated with risk (OR 0.42; 95% CI 0.33–0.54); given the established pro−inflammatory role of lymphotoxin−α in arthritis, this finding should be interpreted with caution (**Supplementary Figure S12**).

Among metabolites, X−12714 exhibited the strongest positive association with RA risk (OR 1.15; 95% CI 1.07–1.24), whereas 3−hydroxyhexanoate was inversely associated with risk (OR 0.89; 95% CI 0.83–0.96) (**Supplementary Figure S13** in **Supplementary 1**). In JIA, the cholesterol to oleoyl−linoleoyl−glycerol (18:1 to 18:2)[2] ratio showed the largest positive association with risk (OR 2.55; 95% CI 1.10–5.91), while propyl 4−hydroxybenzoate sulfate levels were inversely associated (**Supplementary Figure S14**; **Supplementary Tables S3**, **S4** in **Supplementary 1**).

Selected genetically proxied immune−cell and inflammatory protein traits with statistically significant MR associations for RA and/or JIA are summarized in **Table 2**.

**Table 2:** Selected two−sample Mendelian randomization estimates for genetically proxied immune traits and the risk of RA and JIA.

Outcome	Exposure	Category	No. of SNPs	OR (95% CI), IVW	*P* value (IVW)	Q pval (IVW)	Global test *p*	Egger intercept *p*
RA	CD14+ CD16+ monocyte AC	Monocytes	52	0.92 (0.90–0.94)	<0.001	0.568	0.657	0.719
	CD14+ CD16+ monocyte %monocyte	Monocytes	58	0.94 (0.92–0.96)	<0.001	0.183	0.217	0.752
	DC AC	Dendritic cells	38	1.08 (1.05–1.10)	<0.001	0.095	0.137	0.177
	CD14+ CD16- monocyte %monocyte	Monocytes	30	1.09 (1.05–1.12)	<0.001	0.116	0.133	0.066
	CCR2 on myeloid DC	Dendritic cells	22	0.94 (0.91–0.97)	<0.001	0.231	0.257	0.374
	NK AC	NK cells	16	0.93 (0.88–0.98)	0.006	0.051	0.063	0.968
	Interferon gamma levels	Cytokine	18	1.13 (1.04–1.23)	0.004	0.134	0.163	0.092
JIA	CD4 on activated Treg	Regulatory T cells	4	0.37 (0.25–0.56)	<0.001	0.235	0.457	0.176
	CD25 on IgD- CD38-	B cells	5	1.64 (1.29–2.10)	<0.001	0.36	0.417	0.304
	CD8 on CD28- CD8br	CD8+ T cells	5	0.44 (0.28–0.71)	<0.001	0.865	0.863	0.837
	CD25 on IgD+ CD38br	B cells	4	1.72 (1.11–2.65)	0.015	0.083	0.23	0.618
	CD28- CD8br AC	CD8+ T cells	4	1.61 (1.08–2.40)	0.019	0.821	0.867	0.817
	C-C motif chemokine 4 levels	Chemokine	6	0.52 (0.34–0.80)	0.003	0.762	0.741	0.61
	Fms-related tyrosine kinase 3 ligand levels	Soluble factor	4	2.01 (1.04–3.88)	0.038	0.473	0.466	0.274

Odds ratios (ORs) and 95% confidence intervals (CIs) are derived from inverse−variance weighted (IVW) MR and represent the change in odds of RA or JIA per genetically predicted increase in each immune trait. “P value (IVW)” refers to the IVW association; “Q pval (IVW)”, “Global test p”, and “Egger intercept p” correspond to Cochran’s Q test, the MR−PRESSO global test, and the MR−Egger intercept test, respectively, and did not indicate substantial heterogeneity or directional horizontal pleiotropy. “No. of SNPs” indicates the number of genetic instruments per exposure. “Category” denotes the immunological class of each trait (monocytes, dendritic cells, NK cells, regulatory T cells, CD8^+^ T cells, B cells, cytokines, chemokines, and soluble factors). Abbreviations: RA, rheumatoid arthritis; JIA, juvenile idiopathic arthritis; SNP, single−nucleotide polymorphism; NK, natural killer; Treg, regulatory T cell.

## 4 Discussion

This study systematically assessed the global burden of GBD-defined RA among individuals aged 0–19 years from 1990 to 2021 and explored its potential immunological overlap with JIA. In current clinical practice, RA and JIA are classified as distinct entities: RA is a prototypical adult−onset autoimmune arthritis, whereas JIA encompasses several childhood−onset subtypes with heterogeneous clinical and genetic features. Only RF-positive polyarticular JIA closely resembles adult RA in terms of clinical course and HLA background, while other subtypes (e.g., oligoarticular, systemic, enthesitis-related, and psoriatic JIA) exhibit unique patterns of epidemiology, immunopathology, and treatment response. To contextualize our findings and address concerns about conflating these diseases, we summarized the key clinical, epidemiological, and immunogenetic characteristics of the ILAR JIA categories in **Table 3**, highlighting both RA-like and clearly non-RA-like phenotypes.

Within the GBD framework, however, ICD−10 codes for RA and JIA are combined together. The RA estimates in individuals aged 0–19 years are therefore best interpreted as the burden of chronic inflammatory arthritis coded as RA/JIA in childhood and adolescence, rather than “juvenile RA” in a strict nosological sense. Against this background, our GBD analysis showed that age−standardized incidence and prevalence in youth increased steadily over the past three decades, whereas age−standardized DALY rates declined slightly. The higher burden observed in high−SDI regions likely reflects better recognition and coding of pediatric inflammatory arthritis, while lower rates in low−SDI regions probably represent underdiagnosis rather than true protection. Notably, Andean and Tropical Latin America showed a greater burden than expected based on SDI alone, suggesting additional contributions from genetic, environmental, or lifestyle factors, and several high−SDI countries underperformed in the frontier analysis, indicating gaps in early screening and targeted interventions for adolescents (14, 15).

JIA remains the most common chronic inflammatory arthritis of childhood (16). Environmental exposures, such as infections or vaccinations, may trigger synovial inflammation on the background of genetic susceptibility and loss of immune tolerance (3). Although RA and the different JIA subtypes arise at different ages and follow distinct clinical trajectories, they share partially overlapping immunopathology, including T-cell imbalance, activated myeloid cells, and chronic cytokine−driven inflammation. Age-related immune remodeling (e.g., thymic involution, peripheral T-cell dysregulation) and pubertal hormonal changes may help explain concentration of disease burden in adolescents aged 10–19 years and the higher burden in females (5, 17, 18). Immunosenescence and inflammaging may represent crucial mechanisms for differentiating adult and pediatric autoimmune diseases in the future (19, 20). These considerations support the view that some immunological pathways are shared across adult and pediatric inflammatory arthritis, even though RA and most JIA categories are nosologically distinct.

To further explore these pathways, we employed two-sample Mendelian randomization to screen a broad panel of genetically proxied immune-cell, inflammatory protein, and metabolite traits for their associations with RA and JIA risk. Rather than identifying new susceptibility genes, this approach highlights immune traits that may lie on causal pathways. We identified overlapping genetically proxied immune-cell and inflammatory protein traits for RA and JIA, including CD28 expression on CD8^+^CD45RA^+^ T cells, CD25 on memory B cells, and circulating signaling lymphocytic activation molecule (SLAM) and Fms-like tyrosine kinase-3 ligand, indicating shared T-cell co-stimulation and antigen-presentation pathways. In contrast, higher genetically predicted levels of CD4 on activated regulatory T cells, HLA-DR on dendritic cells and myeloid dendritic cells, and CD4^+^ on HLA-DR^+^ CD4^+^ T cells were associated with a lower risk in both diseases, suggesting a common protective role of effective antigen presentation and regulatory T-cell function. Conversely, some traits, such as CD25^hi^ CD45RA^−^ CD4^+^ non−Tregs and the metabolite X−24544, showed discordant associations between RA and JIA, indicating disease−specific immunometabolic mechanisms that may underlie divergent clinical courses and treatment responses (16). The inverse association between genetically proxied ‘TNF−β’ (lymphotoxin−α) levels and JIA risk is counterintuitive, as lymphotoxin−α has been implicated as a pro−inflammatory mediator in RA and related arthritides; this estimate may therefore reflect limitations of the protein GWAS (trait labelling, measurement error, or linkage disequilibrium with neighboring loci) or residual pleiotropy rather than a truly protective effect, and should be interpreted with particular caution.

To better contextualize the relationship between RA and JIA, we also summarized current evidence on the seven ILAR/PRINTO JIA subtypes (**Table 3**). Oligoarticular and RF−negative polyarticular JIA cluster as early−onset, ANA−positive entities with overlapping HLA class II backgrounds and good responses to methotrexate and TNF inhibitors, whereas RF−positive polyarticular JIA most closely resembles adult RA in terms of “shared−epitope” HLA−DRB1 alleles, autoantibody profile and treat−to−target management. By contrast, systemic, enthesitis−related and psoriatic JIA align more with autoinflammatory or spondyloarthritis/psoriatic arthritis spectra and are typically managed with IL−1/IL−6 or TNF−targeted biologics. Taken together, these patterns reinforce that only a subset of JIA (RF−positive polyarticular disease) overlaps substantially with RA, while other subtypes follow distinct clinical and immunological trajectories.

**Table 3:** Summary of clinical, epidemiologic, immunogenetic and therapeutic features across ILAR juvenile idiopathic arthritis subtypes.

Category	Representative clinical features	Geographical variation	Key genetic features	Relationship to adult disease	Treatment strategy
Oligoarticular JIA	Most common JIA subtype; onset typically at 2–4 years, predominantly in girls; ≤4 joints involved in the first 6 months, mainly large lower−limb joints (knees, ankles); high ANA positivity and the greatest risk of chronic anterior uveitis (21, 22).	Accounts for over half of JIA in many cohorts; particularly frequent in Europe and parts of the Middle East (23–26).	Strong associations with HLA−DRB111:03/04, HLA−DRB108:01 and HLA−DPB102:01 (risk) and HLA−DRB115:01 (protection); non−HLA loci including PTPN22 C1858T, STAT4, IL2/IL2RA/IL2RB and IL6/IL6R also contribute (13, 27).	Clusters genetically with RF−negative polyarticular JIA as early−onset ANA−positive autoimmune arthritis, with an HLA pattern distinct from the classic shared−epitope profile of adult RA (21, 28).	Current ACR guidelines recommend intra−articular glucocorticoid injections (± NSAIDs) as initial therapy; csDMARDs (methotrexate preferred) are introduced for poor−prognosis features or IAGC failure, with escalation to TNF inhibitors or other bDMARDs in refractory or extended disease, following a treat−to−target approach and minimizing systemic glucocorticoids (29–31).
RF−negative polyarticular JIA	Onset within 6 months with involvement of ≥5 joints, most often school−age girls; symmetric small−joint and some large−joint arthritis, RF and ACPA negative; overall prognosis better than RF−positive disease (21).	Represents roughly 30% of JIA globally, with somewhat higher proportions reported in North America (23).	Shares an HLA risk profile with oligoarticular JIA (enrichment of HLA−DRB111:03/04, DRB108:01, DPB102:01 and protection by DRB115:01); PTPN22 C1858T confers risk across oligoarticular, RF−negative and RF−positive polyarticular JIA (27, 32).	Genetic and transcriptomic data indicate clustering with oligoarticular JIA as early−onset ANA−positive autoimmune arthritis; although sharing several non−HLA autoimmune loci with RA and SLE, its overall genetic architecture differs from adult RA (21, 28, 33).	For non−systemic polyarthritis, ACR guidelines recommend early csDMARD therapy (methotrexate preferred) rather than long−term NSAIDs alone; inadequate response to methotrexate prompts addition of a bDMARD (such as TNF inhibitors, abatacept or tocilizumab), usually in combination with csDMARDs, while avoiding prolonged systemic glucocorticoid use (30, 31).
RF−positive polyarticular JIA	Comprises ~2–7% of JIA; typically affects adolescent girls with symmetric small−joint arthritis, marked morning stiffness, RF and/or ACPA positivity, and a high risk of erosive joint damage and disability (21, 28).	Relatively uncommon worldwide but reported at higher prevalence in Latin America and parts of south−east Asia (23).	Shows a strongly RA−like genetic profile, with risk alleles at shared−epitope HLA−DRB1*01 and *04, and overlap at non−HLA loci such as PTPN22, STAT4 and TNFAIP3 (21, 27, 34, 35).	Clinical phenotype, serology and genetic architecture closely resemble adult RA, making RF−positive polyarticular JIA the closest pediatric counterpart of adult RA (21, 27, 28).	Managed along the non−systemic polyarticular pathway, but guidelines and treat−to−target recommendations emphasize early, aggressive use of methotrexate followed by prompt escalation to TNF inhibitors or other bDMARDs in non−responders, given the highly erosive course and lower remission rates; chronic systemic glucocorticoids are discouraged (30, 31).
Juvenile psoriatic arthritis (JPsA)	Accounts for ~1–7% of JIA; arthritis with psoriasis, or arthritis plus dactylitis, characteristic nail changes or a first−degree family history of psoriasis; often oligoarticular onset, may evolve to polyarticular disease; dactylitis and chronic uveitis are common; shows a bimodal age distribution (early childhood and later childhood/adolescence) (21, 28).	Usually represents ≤10% of JIA across international and single−country cohorts, with considerable variability related to differences in classification and inclusion criteria (23–26).	Genetic background is heterogeneous: some patients show associations with HLA class II alleles (e.g. HLA−DRB101:01, DQA101:01), while others resemble spondyloarthritis with HLA−B27−related risk; multi−omic analyses support the coexistence of an early−onset ANA−positive JIA−like subgroup and a SpA−like subgroup (27, 28, 33).	Historically grouped with seronegative spondyloarthritis and closely related to adult psoriatic arthritis, but current data indicate only partial overlap, with some patients aligning more with early−onset ANA−positive JIA and others with adult SpA/axial PsA (21, 28).	Current ACR recommendations manage JPsA according to predominant clinical features: peripheral oligo− or polyarthritis follows the oligo-/polyarticular JIA pathway (methotrexate then bDMARDs), whereas prominent enthesitis or sacroiliitis is treated as ERA (NSAIDs then TNF inhibitors); TNF inhibitors are the most commonly used first−line biologics when joint or skin disease is refractory, while evidence for IL−17 and IL−12/23 inhibition in children remains limited (31, 36).
Enthesitis−related arthritis/juvenile spondyloarthritis (ERA/JSpA)	Defined by peripheral arthritis with enthesitis, typically in older boys; asymmetric large−joint involvement of the lower limbs with heel and plantar enthesitis; many patients later develop sacroiliitis and spinal involvement; usually RF/ACPA negative (21, 28).	One of the most frequent subtypes in several Asian cohorts (e.g. parts of Taiwan and India), where ERA may account for more than 30% of JIA (37–39).	Strongly associated with HLA−B27 and shares multiple susceptibility loci with adult spondyloarthritis, particularly within the IL−23/IL−17 axis (27, 28, 34).	Widely considered the pediatric member of the spondyloarthritis family, closely resembling adult axial SpA/ankylosing spondylitis in clinical pattern (axial disease, enthesitis) and genetic background, while presenting additional growth− and development−related issues (21, 28).	ACR guidelines recommend NSAIDs as first−line therapy for active enthesitis or sacroiliitis; persistent axial disease despite adequate NSAIDs should be escalated to TNF inhibitors rather than additional NSAIDs or csDMARDs; methotrexate or sulfasalazine may be used for predominant peripheral arthritis, but have limited efficacy for purely axial disease (30, 31, 36).
Systemic JIA (sJIA)	Systemic inflammatory disease with quotidian high fevers, evanescent salmon−pink rash, lymphadenopathy, hepatosplenomegaly and/or serositis, with arthritis at or after onset; laboratory findings include markedly elevated inflammatory markers and hyperferritinaemia; macrophage activation syndrome is a life−threatening complication (21, 28, 40).	Represents a minority of JIA overall but a relatively larger proportion in several Asian countries and regions (23, 39).	Associated with HLA−DRB1*11 and other MHC class II alleles, but HLA contribution is smaller than in autoimmune JIA subtypes; pathobiology is dominated by innate immune activation with monocyte/macrophage and neutrophil involvement and high levels of IL−1β, IL−6 and IL−18, supporting classification as an autoinflammatory disease (21, 28, 40).	Systematic reviews and EULAR/PReS consensus view sJIA and adult−onset Still’s disease as age−dependent manifestations of a single Still’s disease continuum, distinct from classic adult RA in clinical features, serology and inflammatory pathways (41, 42).	The 2021 ACR guideline places IL−1 and IL−6 inhibitors as first−line therapy for active sJIA (with or without MAS), with glucocorticoids used as short−term adjuncts rather than maintenance therapy; the 2024 EULAR/PReS Still’s disease recommendations further emphasize a “biologics−first” strategy with early IL−1/IL−6 blockade and detailed algorithms for MAS management (29, 31, 42).
Undifferentiated JIA	A diagnosis of exclusion encompassing chronic arthritis that fulfils criteria for no single ILAR/PRINTO category or meets criteria for ≥2 subtypes; therefore clinically heterogeneous, ranging from oligo− and polyarthritis to enthesitis−predominant or mildly systemic phenotypes (21, 28, 43).	By definition a residual category; robust data on global or regional distribution are scarce, and most large international cohorts do not provide detailed analyses for this subgroup (23).	Current immunogenetic studies focus mainly on “classical” subtypes (oligoarticular, RF−negative/positive polyarticular, ERA, systemic JIA, JPsA); there is no consistent evidence for a distinct HLA or non−HLA signature of undifferentiated JIA, and most cases probably inherit risk alleles of the overlapping clinical subtypes (21, 27, 28).	Because this category reflects limitations of current classification rather than a defined biological entity, no clear adult counterpart has been established; undifferentiated JIA is better regarded as a heterogeneous group highlighting the need for biologically driven re−classification (21, 28, 44).	ACR guidelines base recommendations on dominant clinical phenotypes rather than ILAR labels; undifferentiated JIA is therefore treated according to its main manifestations—for example, as non−systemic polyarthritis (methotrexate then bDMARDs), ERA−like disease (NSAIDs then TNF inhibitors) or Still’s disease (early IL−1/IL−6 inhibition)—in line with treat−to−target principles (29–31).

The table summarizes, for each ILAR−defined JIA subtype (oligoarticular, RF−negative and RF−positive polyarticular JIA, JPsA, ERA/JSpA, sJIA and undifferentiated arthritis), the key clinical phenotype, typical geographic distribution, major HLA and non−HLA susceptibility loci, relationships to adult rheumatic diseases and main treatment pathways. Reported proportions are approximate ranges from large national and international cohorts and systematic reviews, intended to indicate relative subtype burden rather than precise prevalence. Abbreviations: JIA, juvenile idiopathic arthritis; JPsA, juvenile psoriatic arthritis; ERA, enthesitis−related arthritis; JSpA, juvenile spondyloarthritis; sJIA, systemic JIA; RF, rheumatoid factor; ACPA, anti−citrullinated peptide antibody; csDMARD, conventional synthetic disease−modifying antirheumatic drug; bDMARD, biologic disease−modifying antirheumatic drug.

This study has several strengths, including use of the most recent GBD 2021 data to characterize the global burden of RA in youth and integration of two−sample MR analyses of genetically proxied immune−cell, inflammatory protein, and metabolite traits for both RA and JIA. Limitations largely reflect constraints of existing data sources: within the GBD framework, RA and JIA are combined and ILAR/PRINTO JIA subtypes are not distinguished, so the burden cannot be disaggregated by specific JIA categories; and the MR analyses relied on GWAS summary statistics mainly from European−ancestry cohorts, including a relatively modest JIA sample, which may limit statistical power and generalizability. As with all MR studies, our causal estimates rest on standard instrumental−variable assumptions, and some residual pleiotropy or bias cannot be entirely excluded despite multiple sensitivity analyses.

## 5 Conclusions

This study systematically assessed the global burden of RA in individuals aged 0–19 years, as defined in the GBD 2021 framework, from 1990 to 2021, highlighting key temporal trends, regional disparities, and associations with sociodemographic development. These findings underscore the need to improve early recognition, timely referral, and preventive strategies in youth populations, particularly in high−burden settings. Our projections suggest that global age−standardized incidence and prevalence rates will rise modestly, whereas age−standardized DALY rates may decline slightly, potentially reflecting, among other factors, advances in diagnosis and treatment. Through two−sample Mendelian randomization analyses, we identified shared and distinct patterns of genetically proxied immune−cell, inflammatory protein, and metabolite traits associated with RA and JIA, highlighting areas of immunological overlap and divergence that may inform molecularly informed approaches to early detection, classification, and prevention across childhood− and adult−onset inflammatory arthritis.

## Data Availability

The original contributions presented in the study are included in the article/**Supplementary Material**. Further inquiries can be directed to the corresponding author/s.
