# Supplementary material for: Global burden and genetic insights of RA and JIA in ages 0–19 years: GBD 2021 and MR analysis
Source: Front Immunol. 2026 Jan 14;16:1661461. doi: 10.3389/fimmu.2025.1661461 (PMC12847325; doi:10.3389/fimmu.2025.1661461)
Supplement: Supplementary file 1 [file DataSheet1.pdf]

Supplementary Materials

1. Supplementary Figures

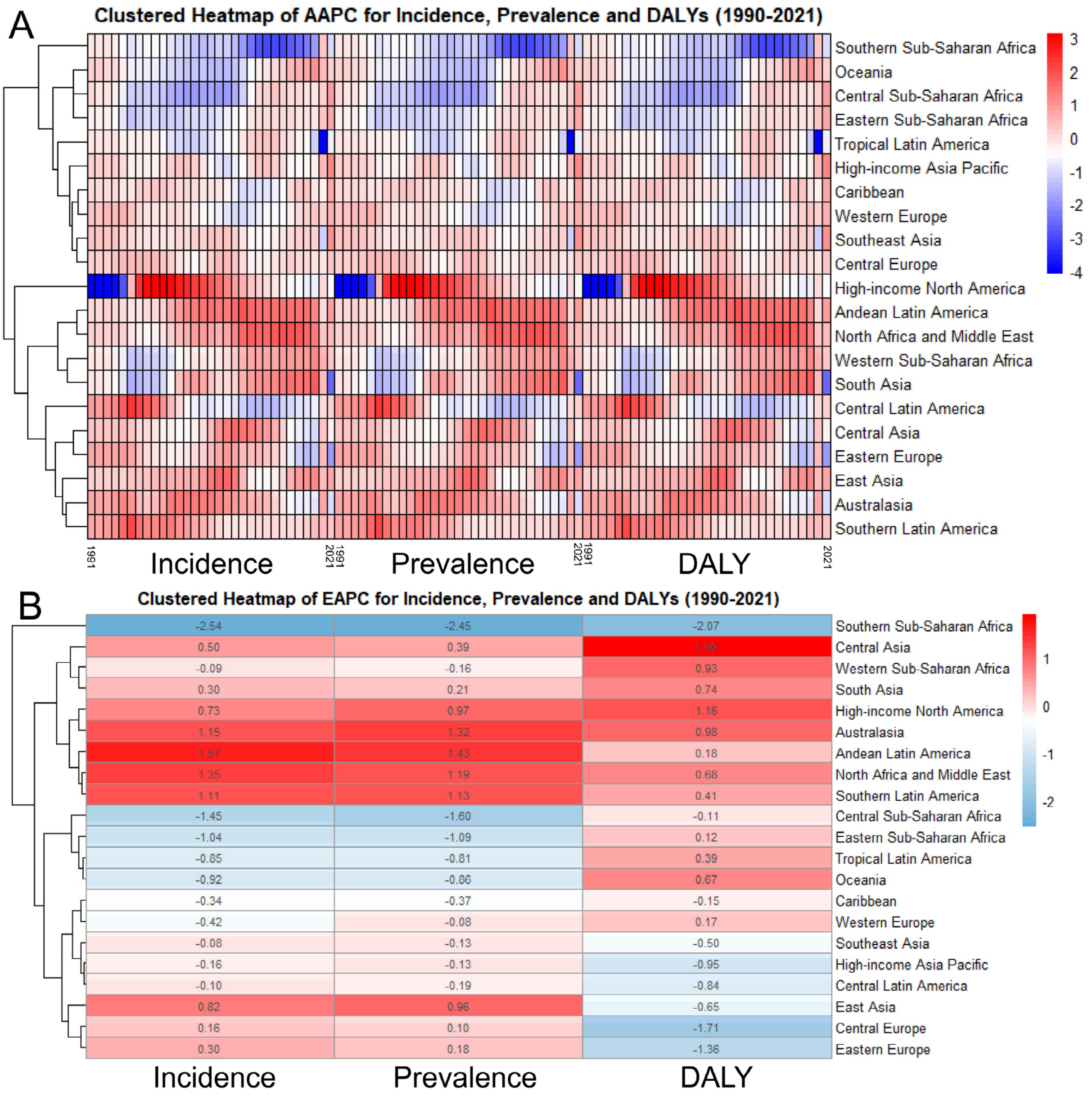

Figure S1. Hierarchical clustering of regional trends in RA burden among children and adolescents, 1990–2021. (A) Heatmap of AAPC in ASIR, ASPR, and ASDR. (B) Heatmap of EAPC for the same metrics. Colors and numbers represent Z-scores rather than raw AAPC or EAPC values, allowing standardized comparison across regions. Positive Z-scores indicate growth rates above the global average; negative Z-scores indicate rates below the global average. RA, rheumatoid arthritis; AAPC, average annual percent change; ASIR, age-standardized incidence rate; ASPR, age-standardized prevalence rate; ASDR, age-standardized disability-adjusted life years rate; EAPC, estimated annual percent change.

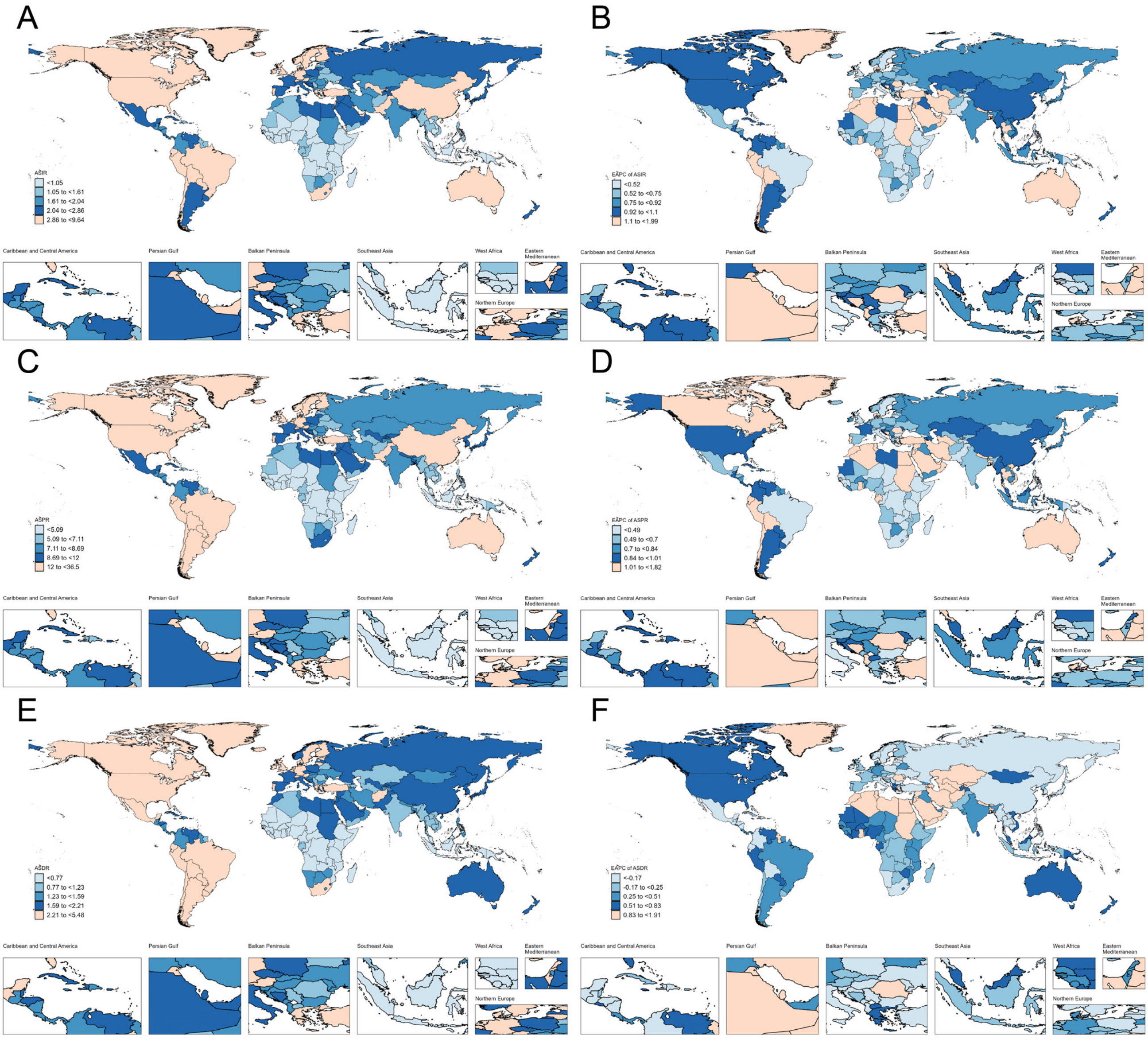

Figure S2. Global burden of disease for RA among children and adolescents in 204 countries and territories. (A) ASIR in 2021; (B) EAPC of ASIR from 1990 to 2021; (C) ASPR in 2021; (D) EAPC of ASPR from 1990 to 2021; (E) ASDR in 2021; (F) EAPC of ASDR from 1990 to 2021. RA, rheumatoid arthritis; EAPC, estimated annual percent change; ASIR, age-standardized incidence rate; ASPR, age-standardized prevalence rate; ASDR, age-standardized disability-adjusted life years rate.

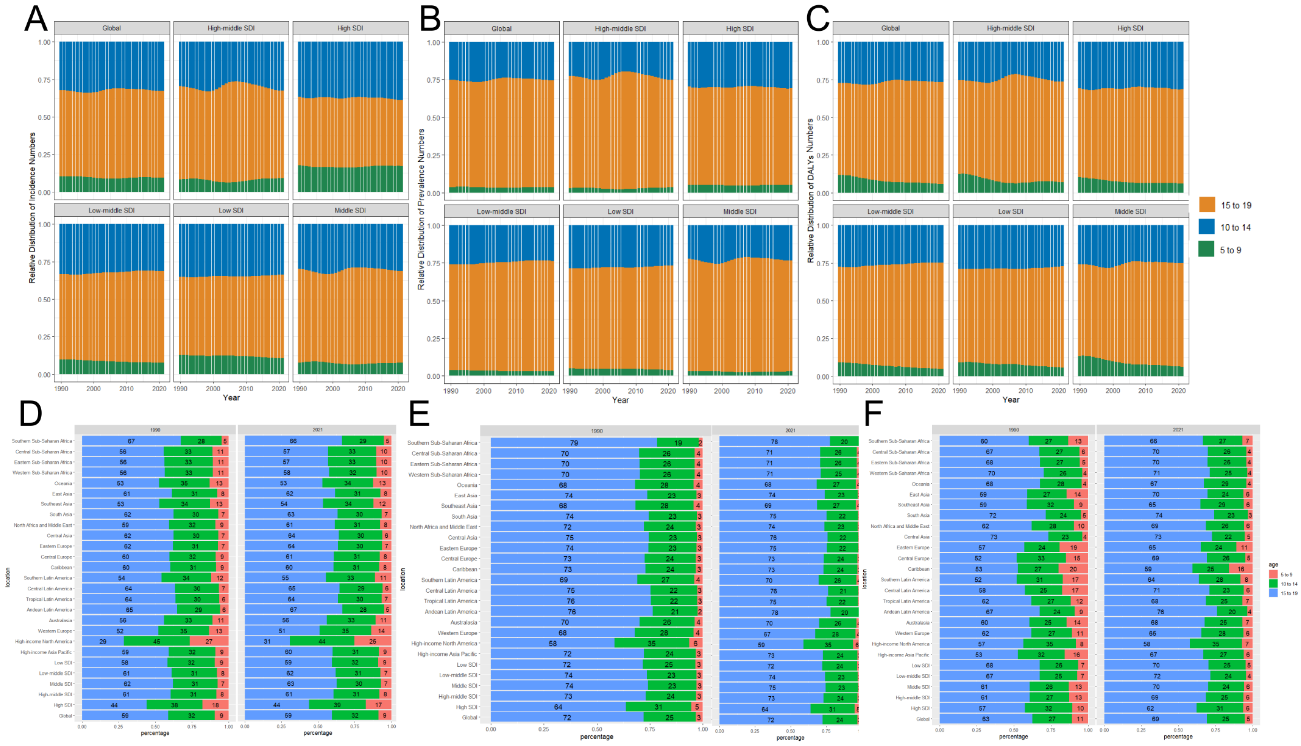

Figure S3. Age distribution of RA burden among children and adolescents by SDI and region, 1990–2021. (A–C) Relative age distribution of incident cases (A), prevalent cases (B), and DALYs (C) by year and SDI levels from 1990 to 2021. Colors represent 3 age groups: 5–9 years (green), 10–14 years (blue), and 15–19 years (orange). (D–F) Stacked bar plots of age group composition by GBD region and SDI in 1990 and 2021 for incident cases (D), prevalent cases (E), and DALYs (F). RA, rheumatoid arthritis; SDI, socio-demographic index; DALYs, disability-adjusted life years; GBD, Global Burden of Disease.

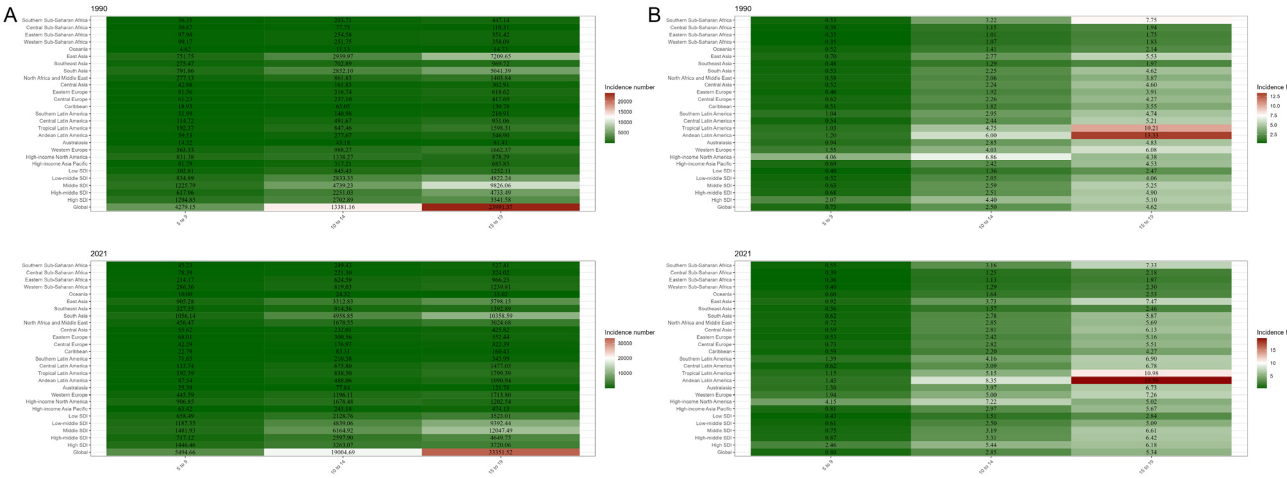

Figure S4. Incidence number and rate of RA in children and adolescents by region and age group in 1990 and 2021. (A) Absolute number of incident RA cases among children and adolescents across GBD regions and SDI quintiles in 1990 (top) and 2021 (bottom), stratified by age groups: 5–9, 10–14, and 15–19 years (B) Corresponding age-specific incidence rates per 100,000 population in 1990 (top) and 2021 (bottom). Heatmap shading represents intensity of burden, with darker red indicating higher incidence values. RA, rheumatoid arthritis; GBD, Global Burden of Disease; SDI, socio-demographic index.

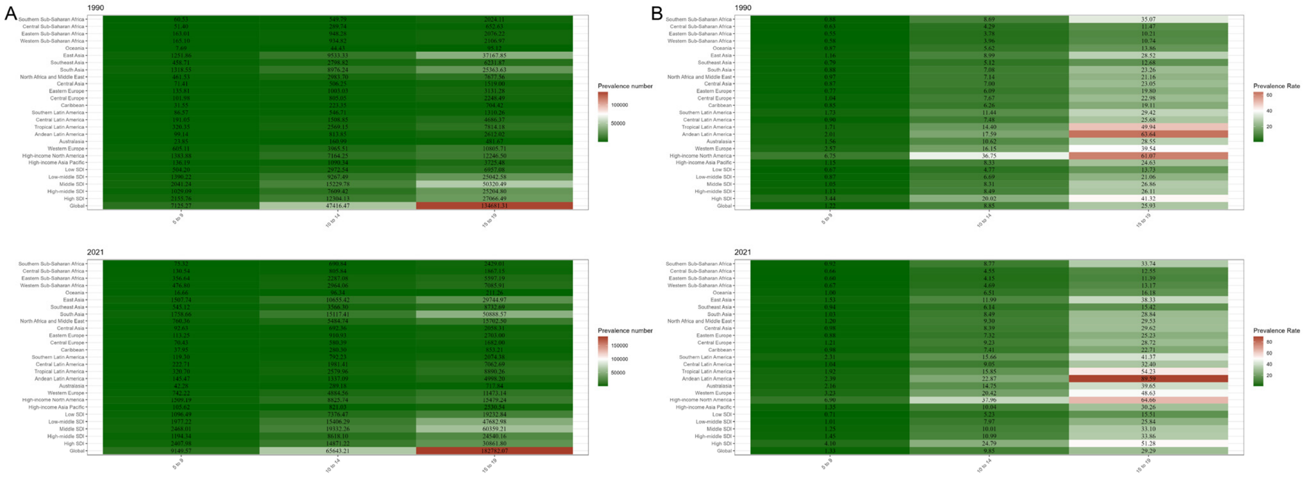

Figure S5. Prevalence number and rate of RA in children and adolescents by region and age group in 1990 and 2021. (A) Absolute number of prevalent RA cases among children and adolescents across GBD regions and SDI quintiles in 1990 (top) and 2021 (bottom), stratified by age groups: 5–9, 10–14, and 15–19 years (B) Corresponding age-specific prevalence rates per 100,000 population in 1990 (top) and 2021 (bottom). Heatmap shading represents intensity of burden, with darker red indicating higher prevalence values. RA, rheumatoid arthritis; GBD, Global Burden of Disease; SDI, socio-demographic index.

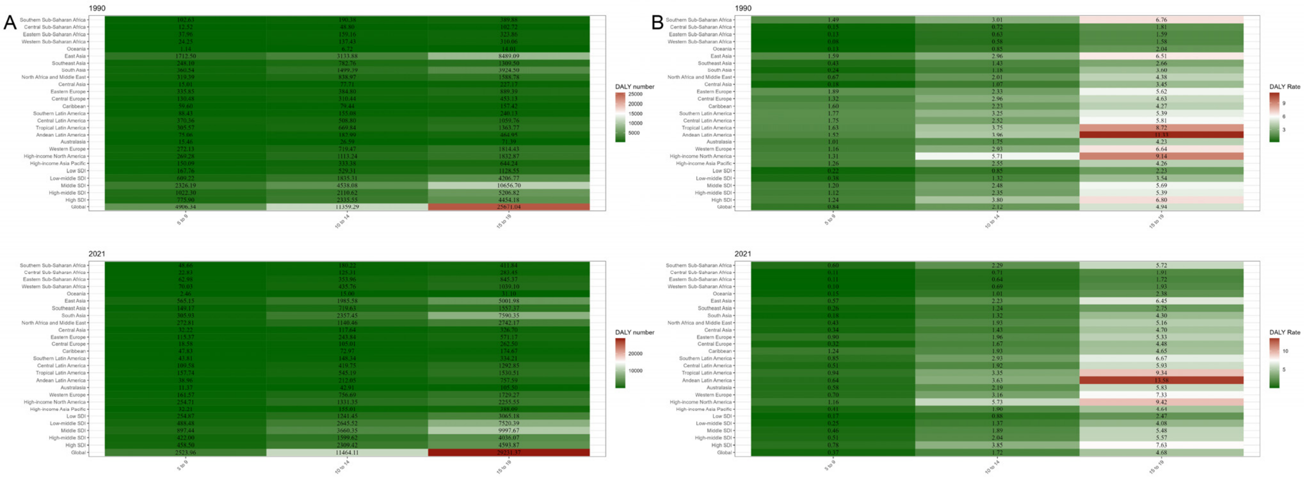

Figure S6. DALY number and rate of RA in children and adolescents by region and age group in 1990 and 2021. (A) Absolute number of DALY RA cases among children and adolescents across GBD regions and SDI quintiles in 1990 (top) and 2021 (bottom), stratified by age groups: 5–9, 10–14, and 15–19 years (B) Corresponding age-specific DALY rates per 100,000 population in 1990 (top) and 2021 (bottom). Heatmap shading represents intensity of burden, with darker red indicating higher DALY values. DALY, disability-adjusted life year; RA, rheumatoid arthritis; GBD, Global Burden of Disease; SDI, socio-demographic index.

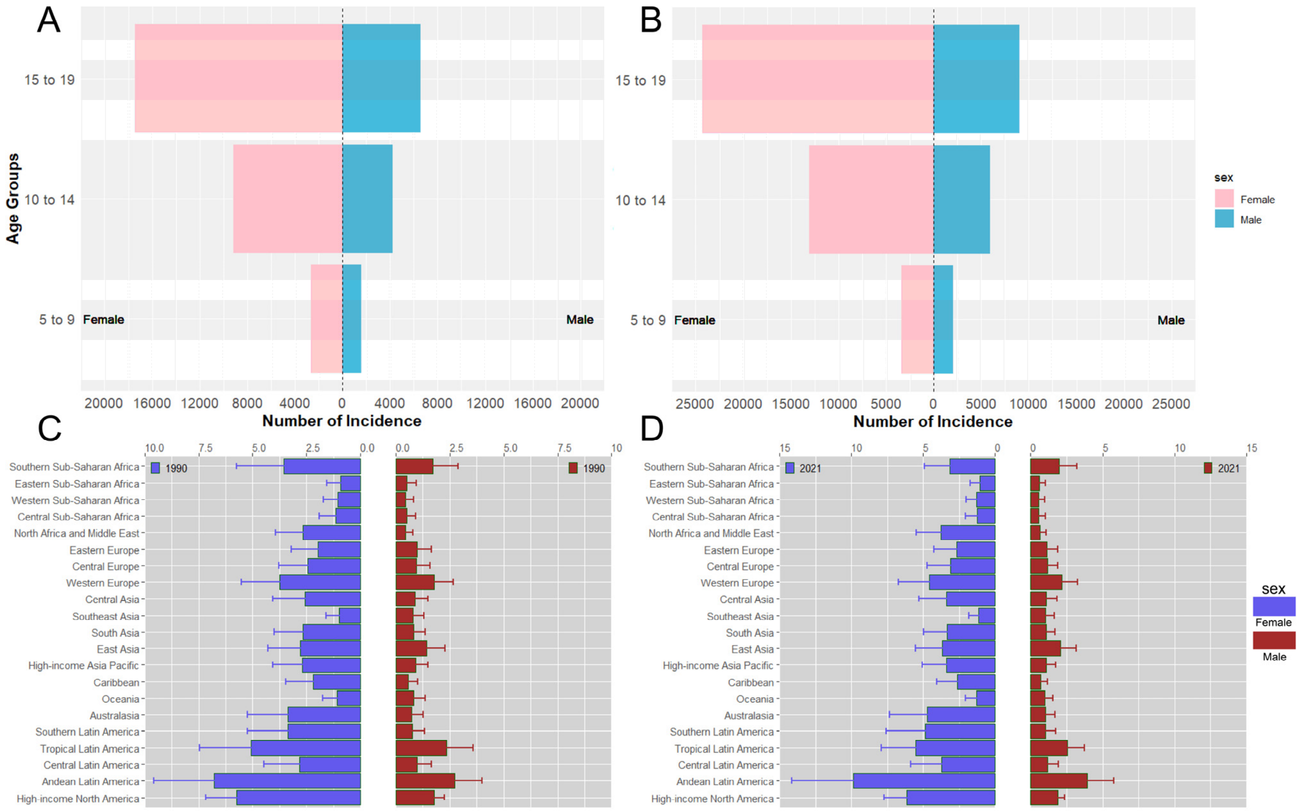

Figure S7. Sex- and age-specific incidence of RA in children and adolescents, 1990 and 2021. (A, B) Population pyramids display the absolute number of new RA cases by sex and age group (5–9, 10–14, and 15–19 years) globally in 1990 (A) and 2021 (B). (C, D) Regional ASIR per 100,000 population by sex in 1990 (C) and 2021 (D) across GBD regions. Error bars represent 95% uncertainty intervals. RA, rheumatoid arthritis; ASIR, age-standardized incidence rate; GBD, Global Burden of Disease.

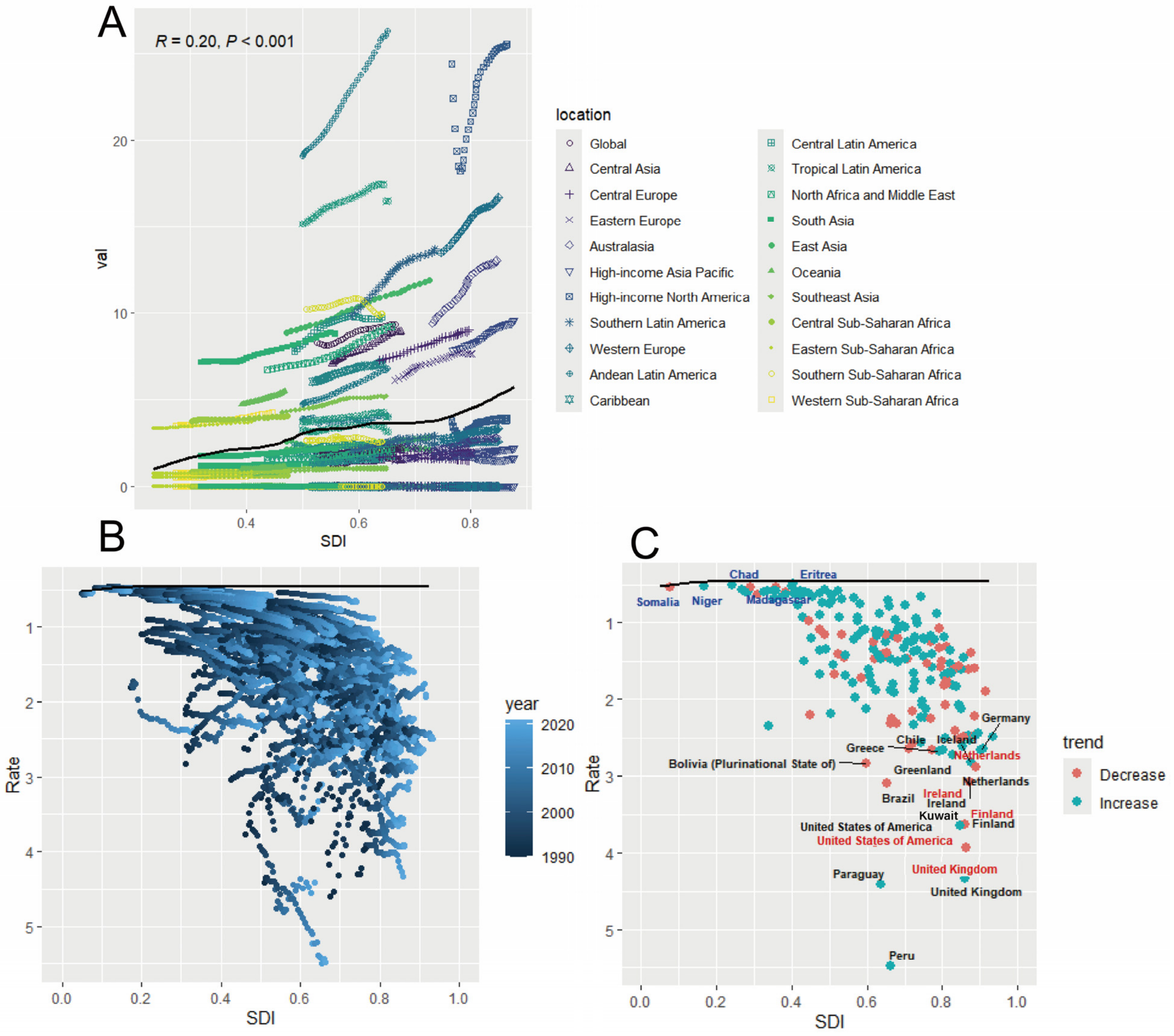

Figure S8. Frontier analysis of RA in children and adolescents based on SDI and ASDR. (A) Association between SDI and ASDR for RA across GBD regions from 1990 to 2021; (B) Temporal trends in ASDR relative to SDI from 1990 to 2021; (C) Scatterplot of individual countries showing the ASDR relative to SDI. The black frontier line represents the potentially achievable ASDR based on SDI. In B and C, the gap between observed points and this line, termed the 'effective difference', indicates the deviation from achievable rates. Red dots show a decrease in age-standardized RA DALY rates from 1990 to 2021, while green dots indicate an increase over the same period. The top 15 countries with the largest effective difference are labelled in black. Countries and territories with low SDI (<0.5) of the top five with the lowest effective difference are labelled in blue. Countries and territories with high SDI (>0.85) of the top five with the highest effective difference are labelled in red. RA, rheumatoid arthritis; SDI, socio-

demographic index; ASDR, age-standardized disability- adjusted life years rate; DALYs, disability-adjusted life years.

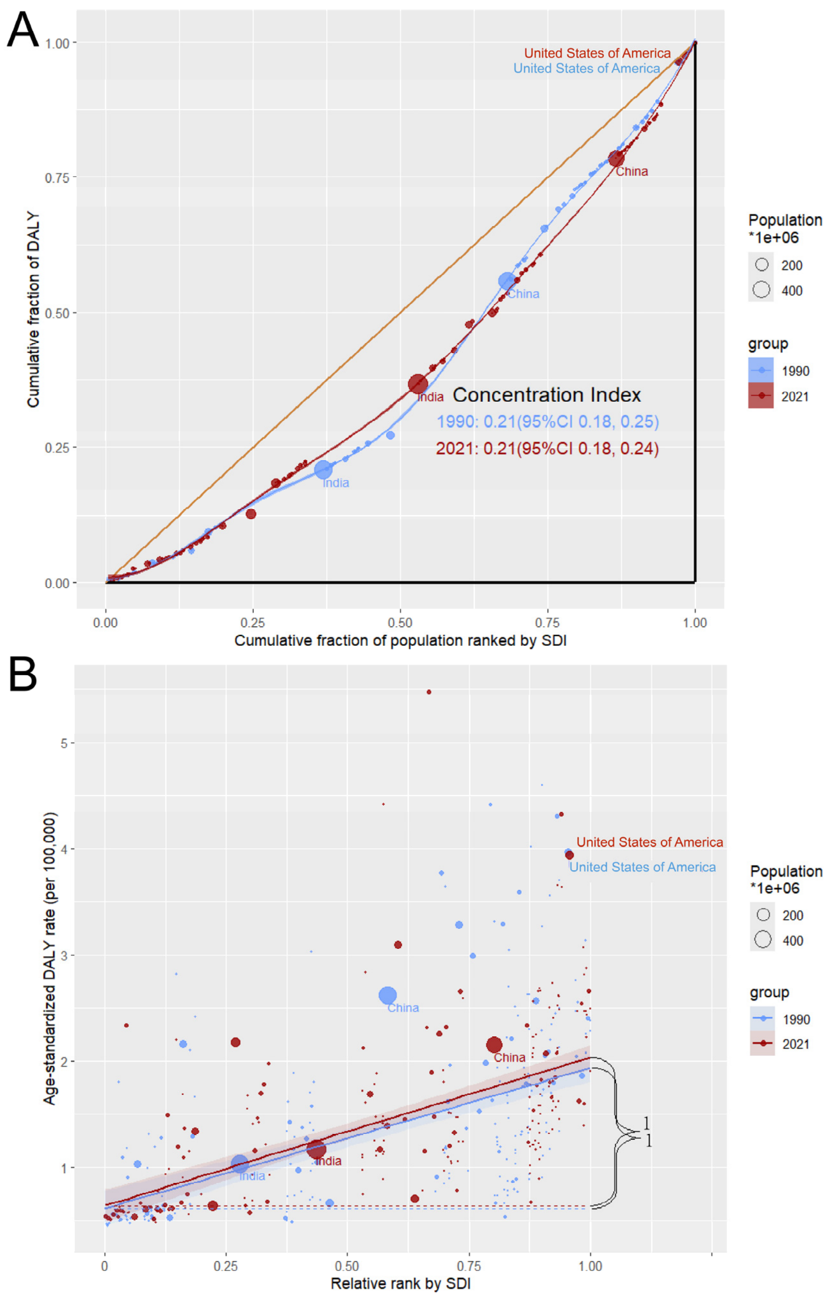

Figure S9. Concentration curves (A) and health inequality regression curves (B) for RA DALYs globally in 1990 and 2021. Larger circles indicate countries with higher populations. The red and blue lines represent linear trends for 1990 and 2021, respectively. The widening gap highlights increasing inequalities in RA burden over time. RA, rheumatoid arthritis; DALYs, disability-adjusted life years.

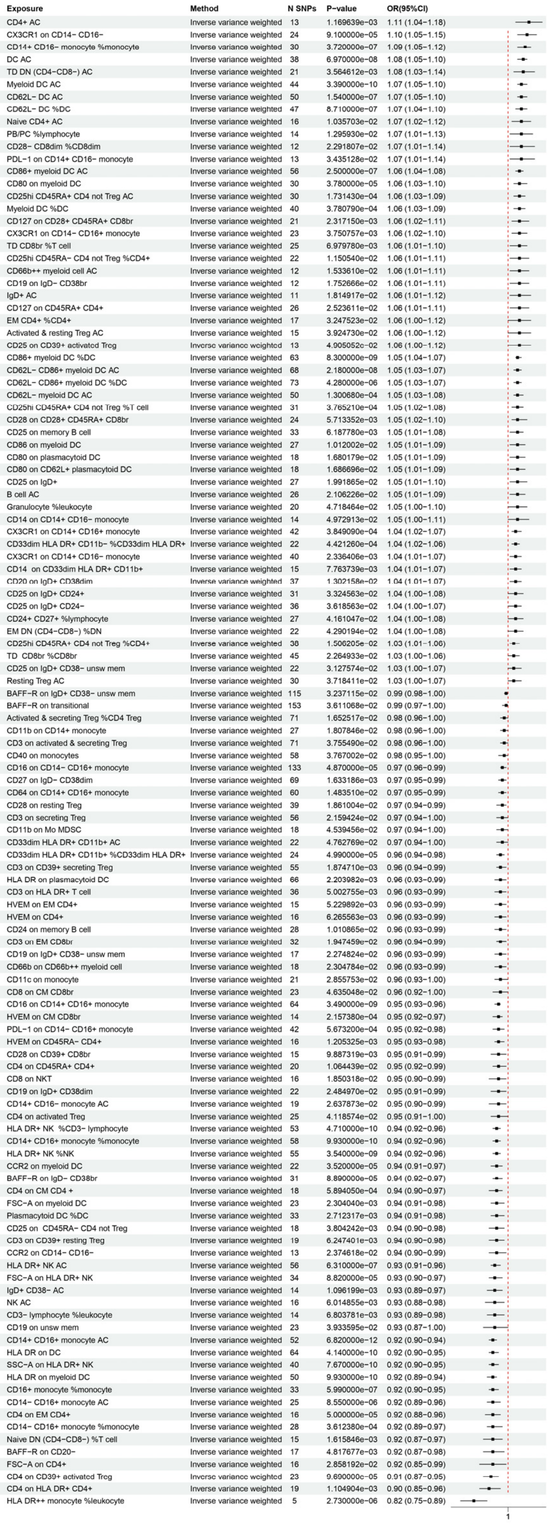

Exposure	Method	N SNPs	P−value	OR(95%CI)
CD4+ AC	Inverse variance weighted	13	1.169639e−03	1.11 (1.04−1.18)
CX3CR1 on CD14− CD16−	Inverse variance weighted	24	9.100000e−05	1.10 (1.05−1.15)
CD14+ CD16− monocyte %monocyte	Inverse variance weighted	30	3.720000e−07	1.09 (1.05−1.12)
DC AC	Inverse variance weighted	38	6.970000e−08	1.08 (1.05−1.10)
TD DN (CD4−CD8−) AC	Inverse variance weighted	21	3.564612e−03	1.08 (1.03−1.14)
Myeloid DC AC	Inverse variance weighted	44	3.390000e−10	1.07 (1.05−1.10)
CD62L− DC AC	Inverse variance weighted	50	1.540000e−07	1.07 (1.05−1.10)
CD62L− DC %DC	Inverse variance weighted	47	8.710000e−07	1.07 (1.04−1.10)
Naive CD4+ AC	Inverse variance weighted	16	1.035703e−02	1.07 (1.02−1.12)
PB/PC %lymphocyte	Inverse variance weighted	14	1.295930e−02	1.07 (1.01−1.13)
CD28− CD8dim %CD8dim	Inverse variance weighted	12	2.291807e−02	1.07 (1.01−1.14)
PDL−1 on CD14+ CD16− monocyte	Inverse variance weighted	13	3.435128e−02	1.07 (1.01−1.14)
CD86+ myeloid DC AC	Inverse variance weighted	56	2.500000e−07	1.06 (1.04−1.08)
CD80 on myeloid DC	Inverse variance weighted	30	3.780000e−05	1.06 (1.03−1.10)
CD25hi CD45RA+ CD4 not Treg AC	Inverse variance weighted	30	1.731430e−04	1.06 (1.03−1.09)
Myeloid DC %DC	Inverse variance weighted	40	3.780790e−04	1.06 (1.03−1.09)
CD127 on CD28+ CD45RA+ CD8br	Inverse variance weighted	21	2.317150e−03	1.06 (1.02−1.11)
CX3CR1 on CD14− CD16+ monocyte	Inverse variance weighted	23	3.750757e−03	1.06 (1.02−1.10)
TD CD8br %T cell	Inverse variance weighted	25	6.979780e−03	1.06 (1.01−1.10)
CD25hi CD45RA− CD4 not Treg %CD4+	Inverse variance weighted	22	1.150540e−02	1.06 (1.01−1.11)
CD66b++ myeloid cell AC	Inverse variance weighted	12	1.533610e−02	1.06 (1.01−1.11)
CD19 on IgD− CD38br	Inverse variance weighted	12	1.752666e−02	1.06 (1.01−1.11)
IgD+ AC	Inverse variance weighted	11	1.814917e−02	1.06 (1.01−1.12)
CD127 on CD45RA+ CD4+	Inverse variance weighted	26	2.523611e−02	1.06 (1.01−1.11)
EM CD4+ %CD4+	Inverse variance weighted	17	3.247523e−02	1.06 (1.00−1.12)
Activated & resting Treg AC	Inverse variance weighted	15	3.924730e−02	1.06 (1.00−1.12)
CD25 on CD39+ activated Treg	Inverse variance weighted	13	4.905052e−02	1.06 (1.00−1.12)
CD86+ myeloid DC %DC	Inverse variance weighted	63	8.300000e−09	1.05 (1.04−1.07)
CD62L− CD86+ myeloid DC AC	Inverse variance weighted	68	2.180000e−08	1.05 (1.03−1.07)
CD62L− CD86+ myeloid DC %DC	Inverse variance weighted	73	4.280000e−06	1.05 (1.03−1.07)
CD62L− myeloid DC AC	Inverse variance weighted	50	1.300680e−04	1.05 (1.03−1.08)
CD25hi CD45RA+ CD4 not Treg %T cell	Inverse variance weighted	31	3.765210e−04	1.05 (1.02−1.08)
CD28 on CD28+ CD45RA+ CD8br	Inverse variance weighted	24	5.713352e−03	1.05 (1.02−1.10)
CD25 on memory B cell	Inverse variance weighted	33	6.187780e−03	1.05 (1.01−1.08)
CD86 on myeloid DC	Inverse variance weighted	27	1.012002e−02	1.05 (1.01−1.09)
CD80 on plasmacytoid DC	Inverse variance weighted	18	1.680179e−02	1.05 (1.01−1.09)
CD80 on CD62L+ plasmacytoid DC	Inverse variance weighted	18	1.686696e−02	1.05 (1.01−1.09)
CD25 on IgD+	Inverse variance weighted	27	1.991865e−02	1.05 (1.01−1.10)
B cell AC	Inverse variance weighted	26	2.106226e−02	1.05 (1.01−1.09)
Granulocyte %leukocyte	Inverse variance weighted	20	4.718464e−02	1.05 (1.00−1.10)
CD14 on CD14+ CD16− monocyte	Inverse variance weighted	14	4.972913e−02	1.05 (1.00−1.11)
CX3CR1 on CD14+ CD16+ monocyte	Inverse variance weighted	42	3.849090e−04	1.04 (1.02−1.07)
CD33dim HLA DR+ CD11b− %CD33dim HLA DR+	Inverse variance weighted	22	4.421260e−04	1.04 (1.02−1.06)
CX3CR1 on CD14+ CD16− monocyte	Inverse variance weighted	40	2.336406e−03	1.04 (1.01−1.07)
CD14 on CD33dim HLA DR+ CD11b+	Inverse variance weighted	15	7.763739e−03	1.04 (1.01−1.07)
CD20 on IgD+ CD38dim	Inverse variance weighted	37	1.302158e−02	1.04 (1.01−1.07)
CD25 on IgD+ CD24+	Inverse variance weighted	31	3.324563e−02	1.04 (1.00−1.08)
CD25 on IgD+ CD24−	Inverse variance weighted	36	3.618563e−02	1.04 (1.00−1.07)
CD24+ CD27+ %lymphocyte	Inverse variance weighted	27	4.161047e−02	1.04 (1.00−1.08)
EM DN (CD4−CD8−) %DN	Inverse variance weighted	22	4.290194e−02	1.04 (1.00−1.08)
CD25hi CD45RA+ CD4 not Treg %CD4+	Inverse variance weighted	38	1.506205e−02	1.03 (1.01−1.06)
TD CD8br %CD8br	Inverse variance weighted	45	2.264933e−02	1.03 (1.00−1.06)
CD25 on IgD+ CD38− unsw mem	Inverse variance weighted	22	3.127574e−02	1.03 (1.00−1.07)
Resting Treg AC	Inverse variance weighted	30	3.718411e−02	1.03 (1.00−1.07)
BAFF−R on IgD+ CD38− unsw mem	Inverse variance weighted	115	3.237115e−02	0.99 (0.98−1.00)
BAFF−R on transitional	Inverse variance weighted	153	3.611068e−02	0.99 (0.97−1.00)
Activated & secreting Treg %CD4 Treg	Inverse variance weighted	71	1.652517e−02	0.98 (0.96−1.00)
CD11b on CD14+ monocyte	Inverse variance weighted	27	1.807846e−02	0.98 (0.96−1.00)
CD3 on activated & secreting Treg	Inverse variance weighted	71	3.755490e−02	0.98 (0.96−1.00)
CD40 on monocytes	Inverse variance weighted	58	3.767002e−02	0.98 (0.95−1.00)
CD16 on CD14− CD16+ monocyte	Inverse variance weighted	133	4.870000e−05	0.97 (0.96−0.99)
CD27 on IgD− CD38dim	Inverse variance weighted	69	1.633186e−03	0.97 (0.95−0.99)
CD64 on CD14+ CD16+ monocyte	Inverse variance weighted	60	1.483510e−02	0.97 (0.95−0.99)
CD28 on resting Treg	Inverse variance weighted	39	1.861004e−02	0.97 (0.94−0.99)
CD3 on secreting Treg	Inverse variance weighted	56	2.159424e−02	0.97 (0.94−1.00)
CD11b on Mo MDSC	Inverse variance weighted	18	4.539456e−02	0.97 (0.94−1.00)
CD33dim HLA DR+ CD11b+ AC	Inverse variance weighted	22	4.762769e−02	0.97 (0.94−1.00)
CD33dim HLA DR+ CD11b+ %CD33dim HLA DR+	Inverse variance weighted	24	4.990000e−05	0.96 (0.94−0.98)
CD3 on CD39+ secreting Treg	Inverse variance weighted	55	1.874710e−03	0.96 (0.94−0.99)
HLA DR on plasmacytoid DC	Inverse variance weighted	66	2.203982e−03	0.96 (0.93−0.99)
CD3 on HLA DR+ T cell	Inverse variance weighted	36	5.002755e−03	0.96 (0.93−0.99)
HVEM on EM CD4+	Inverse variance weighted	15	5.229892e−03	0.96 (0.93−0.99)
HVEM on CD4+	Inverse variance weighted	16	6.265563e−03	0.96 (0.93−0.99)
CD24 on memory B cell	Inverse variance weighted	28	1.010865e−02	0.96 (0.93−0.99)
CD3 on EM CD8br	Inverse variance weighted	32	1.947459e−02	0.96 (0.94−0.99)
CD19 on IgD+ CD38− unsw mem	Inverse variance weighted	17	2.274824e−02	0.96 (0.93−0.99)
CD66b on CD66b++ myeloid cell	Inverse variance weighted	18	2.304784e−02	0.96 (0.93−0.99)
CD11c on monocyte	Inverse variance weighted	21	2.855753e−02	0.96 (0.93−1.00)
CD8 on CM CD8br	Inverse variance weighted	23	4.635048e−02	0.96 (0.92−1.00)
CD16 on CD14+ CD16+ monocyte	Inverse variance weighted	64	3.490000e−09	0.95 (0.93−0.96)
HVEM on CM CD8br	Inverse variance weighted	14	2.157380e−04	0.95 (0.92−0.97)
PDL−1 on CD14− CD16+ monocyte	Inverse variance weighted	42	5.673200e−04	0.95 (0.92−0.98)
HVEM on CD45RA− CD4+	Inverse variance weighted	16	1.205325e−03	0.95 (0.93−0.98)
CD28 on CD39+ CD8br	Inverse variance weighted	15	9.887319e−03	0.95 (0.91−0.99)
CD4 on CD45RA+ CD4+	Inverse variance weighted	20	1.064439e−02	0.95 (0.92−0.99)
CD8 on NKT	Inverse variance weighted	16	1.850318e−02	0.95 (0.90−0.99)
CD19 on IgD+ CD38dim	Inverse variance weighted	22	2.484970e−02	0.95 (0.91−0.99)
CD14+ CD16− monocyte AC	Inverse variance weighted	19	2.637873e−02	0.95 (0.90−0.99)
CD4 on activated Treg	Inverse variance weighted	25	4.118574e−02	0.95 (0.91−1.00)
HLA DR+ NK %CD3− lymphocyte	Inverse variance weighted	53	4.710000e−10	0.94 (0.92−0.96)
CD14+ CD16+ monocyte %monocyte	Inverse variance weighted	58	9.930000e−10	0.94 (0.92−0.96)
HLA DR+ NK %NK	Inverse variance weighted	55	3.540000e−09	0.94 (0.92−0.96)
CCR2 on myeloid DC	Inverse variance weighted	22	3.520000e−05	0.94 (0.91−0.97)
BAFF−R on IgD− CD38br	Inverse variance weighted	31	8.890000e−05	0.94 (0.92−0.97)
CD4 on CM CD4 +	Inverse variance weighted	18	5.894050e−04	0.94 (0.90−0.97)
FSC−A on myeloid DC	Inverse variance weighted	23	2.304040e−03	0.94 (0.91−0.98)
Plasmacytoid DC %DC	Inverse variance weighted	33	2.712317e−03	0.94 (0.91−0.98)
CD25 on CD45RA− CD4 not Treg	Inverse variance weighted	18	3.804242e−03	0.94 (0.90−0.98)
CD3 on CD39+ resting Treg	Inverse variance weighted	19	6.247401e−03	0.94 (0.90−0.98)
CCR2 on CD14− CD16−	Inverse variance weighted	13	2.374618e−02	0.94 (0.90−0.99)
HLA DR+ NK AC	Inverse variance weighted	56	6.310000e−07	0.93 (0.91−0.96)
FSC−A on HLA DR+ NK	Inverse variance weighted	34	8.820000e−05	0.93 (0.90−0.97)
IgD+ CD38− AC	Inverse variance weighted	14	1.096199e−03	0.93 (0.89−0.97)
NK AC	Inverse variance weighted	16	6.014855e−03	0.93 (0.88−0.98)
CD3− lymphocyte %leukocyte	Inverse variance weighted	14	6.803781e−03	0.93 (0.89−0.98)
CD19 on unsw mem	Inverse variance weighted	23	3.933595e−02	0.93 (0.87−1.00)
CD14+ CD16+ monocyte AC	Inverse variance weighted	52	6.820000e−12	0.92 (0.90−0.94)
HLA DR on DC	Inverse variance weighted	64	4.140000e−10	0.92 (0.90−0.95)
SSC−A on HLA DR+ NK	Inverse variance weighted	40	7.670000e−10	0.92 (0.90−0.95)
HLA DR on myeloid DC	Inverse variance weighted	50	9.930000e−10	0.92 (0.89−0.94)
CD16+ monocyte %monocyte	Inverse variance weighted	33	5.990000e−07	0.92 (0.90−0.95)
CD14− CD16+ monocyte AC	Inverse variance weighted	25	8.550000e−06	0.92 (0.89−0.96)
CD4 on EM CD4+	Inverse variance weighted	16	5.000000e−05	0.92 (0.88−0.96)
CD14− CD16+ monocyte %monocyte	Inverse variance weighted	28	3.612380e−04	0.92 (0.89−0.97)
Naive DN (CD4−CD8−) %T cell	Inverse variance weighted	15	1.615846e−03	0.92 (0.87−0.97)
BAFF−R on CD20−	Inverse variance weighted	17	4.817677e−03	0.92 (0.87−0.98)
FSC−A on CD4+	Inverse variance weighted	16	2.858192e−02	0.92 (0.85−0.99)
CD4 on CD39+ activated Treg	Inverse variance weighted	23	9.690000e−05	0.91 (0.87−0.95)
CD4 on HLA DR+ CD4+	Inverse variance weighted	19	1.104904e−03	0.90 (0.85−0.96)
HLA DR++ monocyte %leukocyte	Inverse variance weighted	5	2.730000e−06	0.82 (0.75−0.89)

Figure S10. Mendelian randomization estimates of the causal associations between immune cells and RA. The forest plot shows the statistically significant results of IVW Mendelian randomization analyses evaluating the associations between 731 immune cells and the risk of RA. Each row represents a specific immune cell, with ORs and 95% CIs plotted. ORs greater than 1 indicate increased risk, and ORs less than 1 indicate a protective effect. RA, rheumatoid arthritis; IVW, inverse-variance weighted; OR, odds ratio; CI, confidence intervals.

Exposure	Method	N SNPs	P−value	OR(95%CI)
Interferon gamma levels	Inverse variance weighted	18	0.003908355	1.13 (1.04−1.23)
CD40L receptor levels	Inverse variance weighted	46	0.000002440	1.12 (1.07−1.18)
C−X−C motif chemokine 10 levels	Inverse variance weighted	31	0.012361037	1.09 (1.02−1.16)
Tumor necrosis factor levels	Inverse variance weighted	25	0.021107203	1.09 (1.01−1.18)
Interleukin−17A levels	Inverse variance weighted	19	0.044023253	1.09 (1.00−1.18)
Fms−related tyrosine kinase 3 ligand levels	Inverse variance weighted	54	0.006263796	1.08 (1.02−1.15)
Fibroblast growth factor 21 levels	Inverse variance weighted	34	0.006317857	1.08 (1.02−1.15)
Natural killer cell receptor 2B4 levels	Inverse variance weighted	41	0.004152972	1.07 (1.02−1.13)
Monocyte chemoattractant protein−4 levels	Inverse variance weighted	46	0.006100844	1.07 (1.02−1.13)
T−cell surface glycoprotein CD6 isoform levels	Inverse variance weighted	60	0.000050600	1.06 (1.03−1.09)
Interleukin−10 receptor subunit beta levels	Inverse variance weighted	54	0.000786742	1.06 (1.02−1.09)
Interleukin−18 levels	Inverse variance weighted	46	0.006734549	1.06 (1.02−1.11)
Signaling lymphocytic activation molecule levels	Inverse variance weighted	43	0.037778866	1.06 (1.00−1.11)
Interleukin−10 levels	Inverse variance weighted	33	0.042559937	1.06 (1.00−1.13)
C−C motif chemokine 25 levels	Inverse variance weighted	108	0.001776546	0.97 (0.95−0.99)
C−C motif chemokine 23 levels	Inverse variance weighted	44	0.000854939	0.95 (0.92−0.98)
Leukemia inhibitory factor receptor levels	Inverse variance weighted	40	0.001734164	0.93 (0.89−0.97)
Fibroblast growth factor 19 levels	Inverse variance weighted	34	0.004992198	0.92 (0.87−0.97)
Interleukin−6 levels	Inverse variance weighted	15	0.020478960	0.90 (0.83−0.98)

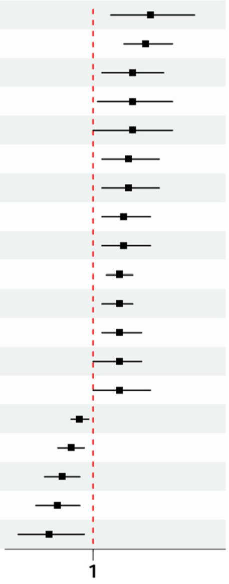

Figure S11. Mendelian randomization estimates of the causal associations between circulating inflammatory proteins and RA. The forest plot shows the statistically significant results of IVW Mendelian randomization analyses evaluating the associations between 91 circulating inflammatory proteins and the risk of RA. Each row represents a specific circulating inflammatory protein, with ORs and 95% CIs plotted. ORs greater than 1 indicate increased risk, and ORs less than 1 indicate a protective effect. RA, rheumatoid arthritis; IVW, inverse-variance weighted; OR, odds ratio; CI, confidence intervals

Exposure	Method	N SNPs	P−value	OR(95%CI)
C−X−C motif chemokine 9 levels	Inverse variance weighted	3	2.470000e−10	10.97 (5.23−23.05)
Signaling lymphocytic activation molecule levels	Inverse variance weighted	4	1.235992e−02	7.53 (1.55−36.60)
T−cell surface glycoprotein CD5 levels	Inverse variance weighted	3	1.401560e−04	4.22 (2.01− 8.85)
Fms−related tyrosine kinase 3 ligand levels	Inverse variance weighted	4	3.799495e−02	2.01 (1.04− 3.88)
C−C motif chemokine 4 levels	Inverse variance weighted	6	3.219076e−03	0.52 (0.34− 0.80)
TNF−beta levels	Inverse variance weighted	12	4.160000e−11	0.42 (0.33− 0.54)

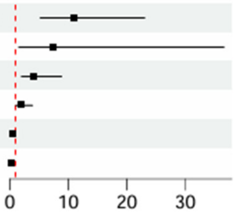

Figure S12. Mendelian randomization estimates of the causal associations between circulating inflammatory proteins and JIA. The forest plot shows the statistically significant results of IVW Mendelian randomization analyses evaluating the associations between 91 circulating inflammatory proteins and the risk of JIA. Each row represents a specific circulating inflammatory protein, with ORs and 95% CIs plotted. ORs greater than 1 indicate increased risk, and ORs less than 1 indicate a protective effect. JIA, juvenile idiopathic arthritis; IVW, inverse-variance weighted; OR, odds ratio; CI, confidence intervals.

Exposure	Method	N SNPs	P−value	OR(95%CI)
X−12714 levels	Inverse variance weighted	20	2.919440e−04	1.15 (1.07−1.24)
X−25828 levels	Inverse variance weighted	14	1.567641e−02	1.14 (1.03−1.27)
3−hydroxylaurate levels	Inverse variance weighted	9	2.489294e−02	1.13 (1.02−1.25)
1−(1−enyl−palmitoyl)−2−arachidonoyl−GPE (p−16:0/20:4) levels	Inverse variance weighted	23	1.660400e−04	1.12 (1.06−1.19)
X−17335 levels	Inverse variance weighted	12	1.261090e−02	1.12 (1.03−1.23)
Adenosine 5'−monophosphate (AMP) to flavin adenine dinucleotide (FAD) ratio	Inverse variance weighted	12	5.081310e−03	1.11 (1.03−1.20)
3−methyl−2−oxovalerate to 3−methyl−2−oxobutyrate ratio	Inverse variance weighted	21	8.804203e−03	1.11 (1.03−1.21)
Adenosine 5'−monophosphate (AMP) to serine ratio	Inverse variance weighted	13	1.016049e−02	1.11 (1.03−1.20)
3−hydroxyoctanoylcarnitine (2) levels	Inverse variance weighted	13	1.411405e−02	1.11 (1.02−1.20)
Picolinoylglycine levels	Inverse variance weighted	24	4.698156e−03	1.10 (1.03−1.17)
Adenosine 5'−diphosphate (ADP) to glycerate ratio	Inverse variance weighted	13	7.886283e−03	1.10 (1.03−1.18)
Arginine to citrulline ratio	Inverse variance weighted	36	7.886288e−03	1.10 (1.02−1.17)
X−21442 levels	Inverse variance weighted	16	8.181042e−03	1.10 (1.03−1.18)
Leucine to N−palmitoyl−sphingosine (d18:1 to 16:0) ratio	Inverse variance weighted	20	2.187426e−02	1.10 (1.01−1.19)
Glucose to N−palmitoyl−sphingosine (d18:1 to 16:0) ratio	Inverse variance weighted	22	1.352021e−02	1.09 (1.02−1.17)
Palmitoleoylcarnitine (C16:1) levels	Inverse variance weighted	16	3.707219e−02	1.09 (1.01−1.18)
Glycolithocholate to glycolithocholate sulfate ratio	Inverse variance weighted	30	1.181528e−03	1.08 (1.03−1.13)
1−palmitoyl−2−linoleoyl−GPI (16:0/18:2) levels	Inverse variance weighted	22	4.819346e−03	1.08 (1.02−1.15)
1−(1−enyl−stearoyl)−2−oleoyl−GPE (p−18:0/18:1) levels	Inverse variance weighted	19	1.202443e−02	1.08 (1.02−1.16)
Stearoylcarnitine levels	Inverse variance weighted	23	1.301050e−02	1.08 (1.02−1.14)
Succinate to acetoacetate ratio	Inverse variance weighted	13	2.207590e−02	1.08 (1.01−1.16)
5−hydroxymethyl−2−furoylcarnitine levels	Inverse variance weighted	21	2.235327e−02	1.08 (1.01−1.16)
X−24334 levels	Inverse variance weighted	17	3.103452e−02	1.08 (1.01−1.15)
Formiminoglutamate levels	Inverse variance weighted	24	3.209554e−02	1.08 (1.01−1.15)
1−(1−enyl−stearoyl)−2−arachidonoyl−GPE (p−18:0/20:4) levels	Inverse variance weighted	32	3.135217e−03	1.07 (1.02−1.11)
Adenosine 5'−diphosphate (ADP) to mannose ratio	Inverse variance weighted	18	1.099763e−02	1.07 (1.01−1.12)
Thioproline levels	Inverse variance weighted	23	2.481114e−02	1.07 (1.01−1.14)
Ursodeoxycholate levels	Inverse variance weighted	18	3.312845e−02	1.07 (1.01−1.15)
Gamma−glutamylglutamate levels	Inverse variance weighted	30	4.534792e−02	1.07 (1.00−1.14)
Cortisol levels (plasma)	Inverse variance weighted	19	4.886221e−02	1.07 (1.00−1.15)
X−24544 levels	Inverse variance weighted	60	4.180000e−05	1.06 (1.03−1.09)
Ximenoylcarnitine (C26:1) levels	Inverse variance weighted	37	2.231730e−03	1.06 (1.02−1.11)
Adenosine 5'−diphosphate (ADP) to flavin adenine dinucleotide (FAD) ratio	Inverse variance weighted	22	4.248732e−03	1.06 (1.02−1.11)
Nervonoylcarnitine (C24:1) levels	Inverse variance weighted	30	7.411309e−03	1.06 (1.02−1.11)
Adenosine 5'−monophosphate (AMP) to glycine ratio	Inverse variance weighted	24	1.663419e−02	1.06 (1.01−1.12)
Palmitoyl dihydrosphingomyelin (d18:0/16:0) levels	Inverse variance weighted	35	1.723876e−02	1.06 (1.01−1.12)
Alpha−ketoglutaramate levels	Inverse variance weighted	25	2.356109e−02	1.06 (1.01−1.12)
Hydantoin−5−propionate levels	Inverse variance weighted	19	4.199443e−02	1.06 (1.00−1.13)
N−acetylputrescine levels	Inverse variance weighted	40	1.930071e−03	1.05 (1.02−1.08)
Malate levels	Inverse variance weighted	18	4.077921e−03	1.05 (1.02−1.09)
Succinimide levels	Inverse variance weighted	19	2.105272e−02	1.05 (1.01−1.09)
X−16087 levels	Inverse variance weighted	29	3.116308e−02	1.05 (1.00−1.10)
X−17653 levels	Inverse variance weighted	28	3.117689e−02	1.05 (1.00−1.10)
Carnitine to ergothioneine ratio	Inverse variance weighted	27	4.904901e−02	1.05 (1.00−1.11)
Isoleucine levels	Inverse variance weighted	16	4.976726e−02	1.05 (1.00−1.10)
X−12798 levels	Inverse variance weighted	70	7.828190e−04	1.04 (1.02−1.06)
Arachidonoylcarnitine (C20:4) levels	Inverse variance weighted	79	4.932147e−03	1.04 (1.01−1.06)
X−21355 levels	Inverse variance weighted	15	1.069200e−02	1.04 (1.01−1.07)
1−linoleoyl−2−arachidonoyl−GPC (18:2/20:4n6) levels	Inverse variance weighted	37	1.866324e−02	1.04 (1.01−1.08)
Carnitine C18:2 levels	Inverse variance weighted	40	3.350010e−02	1.04 (1.00−1.07)
Urea levels	Inverse variance weighted	23	3.428780e−02	1.04 (1.00−1.08)
Lignoceroylcarnitine (C24) levels	Inverse variance weighted	33	3.880735e−02	1.04 (1.00−1.08)
X−12830 levels	Inverse variance weighted	25	4.908729e−02	1.04 (1.00−1.09)
5−acetylamino−6−formylamino−3−methyluracil levels	Inverse variance weighted	50	1.043017e−02	1.03 (1.01−1.05)
Biliverdin levels	Inverse variance weighted	49	2.353526e−02	1.03 (1.00−1.06)
Dodecadienoate (12:2) levels	Inverse variance weighted	29	3.368695e−02	1.03 (1.00−1.05)
4−guanidinobutanoate levels	Inverse variance weighted	48	3.685297e−02	1.03 (1.00−1.06)
1−(1−enyl−palmitoyl)−2−arachidonoyl−gpc (p−16:0/20:4) levels	Inverse variance weighted	59	4.354232e−02	1.03 (1.00−1.06)
X−12112 levels	Inverse variance weighted	98	1.880730e−02	0.98 (0.96−1.00)
Paraxanthine to 5−acetylamino−6−formylamino−3−methyluracil ratio	Inverse variance weighted	64	1.443961e−03	0.97 (0.96−0.99)
Butyrylglycine levels	Inverse variance weighted	47	1.047078e−02	0.97 (0.95−0.99)
Decadienedioic acid (C10:2−DC) levels	Inverse variance weighted	67	1.589002e−02	0.97 (0.95−0.99)
Ferulic acid 4−sulfate levels	Inverse variance weighted	36	2.223213e−02	0.97 (0.94−1.00)
1−palmitoyl−2−linoleoyl−GPE (16:0/18:2) levels	Inverse variance weighted	53	3.207547e−02	0.97 (0.95−1.00)
Glycine to alanine ratio	Inverse variance weighted	36	4.206545e−02	0.97 (0.93−1.00)
Glutarylcarnitine (c5−dc) levels	Inverse variance weighted	66	4.497740e−02	0.97 (0.94−1.00)
5−hydroxylysine levels	Inverse variance weighted	47	4.566045e−02	0.97 (0.94−1.00)
N−acetyl−aspartyl−glutamate (naag) levels	Inverse variance weighted	139	4.860000e−10	0.96 (0.94−0.97)
Metabolonic lactone sulfate levels	Inverse variance weighted	72	3.125110e−04	0.96 (0.94−0.98)
X−26109 levels	Inverse variance weighted	51	6.403940e−04	0.96 (0.94−0.98)
Glucuronide of piperine metabolite C17H21NO3 (3) levels	Inverse variance weighted	16	3.680059e−03	0.96 (0.93−0.99)
Octanoylcarnitine (c8) levels	Inverse variance weighted	48	2.257177e−02	0.96 (0.93−0.99)
Tryptophan betaine levels	Inverse variance weighted	44	3.063876e−02	0.96 (0.93−1.00)
Succinylcarnitine levels	Inverse variance weighted	45	3.244435e−02	0.96 (0.93−1.00)
Glucuronide of piperine metabolite C17H21NO3 (4) levels	Inverse variance weighted	24	4.904164e−02	0.96 (0.92−1.00)
2−hydroxy−4−(methylthio)butanoic acid levels	Inverse variance weighted	48	1.141838e−02	0.95 (0.92−0.99)
Serine to threonine ratio	Inverse variance weighted	33	1.471095e−02	0.95 (0.91−0.99)
Hexanoylglutamine levels	Inverse variance weighted	47	1.640434e−02	0.95 (0.91−0.99)
1−stearoyl−2−linoleoyl−GPI (18:0/18:2) levels	Inverse variance weighted	35	2.342685e−02	0.95 (0.91−0.99)
Dihydrocaffeate sulfate (2) levels	Inverse variance weighted	29	2.757668e−02	0.95 (0.90−0.99)
Allantoin levels	Inverse variance weighted	24	3.165203e−02	0.95 (0.91−1.00)
Hydroxypalmitoyl sphingomyelin (d18:1/16:0(OH)) levels	Inverse variance weighted	38	3.710712e−02	0.95 (0.90−1.00)
Phosphate to urate ratio	Inverse variance weighted	43	3.797429e−02	0.95 (0.91−1.00)
Adenosine 5'−diphosphate (ADP) to glycerol ratio	Inverse variance weighted	20	4.048336e−02	0.95 (0.91−1.00)
4−cholesten−3−one levels	Inverse variance weighted	21	4.625657e−02	0.95 (0.90−1.00)
N−formylanthranilic acid levels	Inverse variance weighted	39	2.980000e−05	0.94 (0.91−0.97)
Propionylglycine levels	Inverse variance weighted	25	1.519743e−03	0.94 (0.90−0.98)
Cysteinylglycine disulfide levels	Inverse variance weighted	42	1.852618e−03	0.94 (0.90−0.98)
X−24306 levels	Inverse variance weighted	25	3.578389e−03	0.94 (0.90−0.98)
S−1−pyrroline−5−carboxylate levels	Inverse variance weighted	34	7.658483e−03	0.94 (0.91−0.98)
N−acetylglucosamine/n−acetylgalactosamine levels	Inverse variance weighted	23	1.163482e−02	0.94 (0.89−0.99)
X−12127 levels	Inverse variance weighted	34	1.749098e−02	0.94 (0.90−0.99)
N−acetylalliin levels	Inverse variance weighted	25	1.922345e−02	0.94 (0.90−0.99)
Cysteinylglycine to taurine ratio	Inverse variance weighted	33	1.948833e−02	0.94 (0.90−0.99)
Alpha−hydroxycaproate levels	Inverse variance weighted	16	2.391467e−02	0.94 (0.90−0.99)
X−11372 levels	Inverse variance weighted	27	2.547203e−02	0.94 (0.89−0.99)
Carotene diol (2) levels	Inverse variance weighted	23	2.749782e−02	0.94 (0.88−0.99)
Glucuronide of C10H18O2 (7) levels	Inverse variance weighted	21	3.262135e−02	0.94 (0.88−0.99)
Uridine to 2'−deoxyuridine ratio	Inverse variance weighted	26	3.592524e−02	0.94 (0.89−1.00)
1−stearoyl−2−linoleoyl−gpc (18:0/18:2) levels	Inverse variance weighted	25	3.726940e−02	0.94 (0.89−1.00)
Hypotaurine to cysteine ratio	Inverse variance weighted	31	4.157524e−02	0.94 (0.89−1.00)
X−12849 levels	Inverse variance weighted	24	4.624094e−02	0.94 (0.89−1.00)
Trans−2−hexenoylglycine levels	Inverse variance weighted	28	4.727352e−02	0.94 (0.89−1.00)
N−acetylvaline levels	Inverse variance weighted	19	4.774809e−02	0.94 (0.88−1.00)
4−allylphenol sulfate levels	Inverse variance weighted	23	4.952844e−02	0.94 (0.89−1.00)
Mannose to glycerol ratio	Inverse variance weighted	19	1.022456e−02	0.93 (0.88−0.98)
X−11880 levels	Inverse variance weighted	17	2.259813e−02	0.93 (0.87−0.99)
2−linoleoylglycerol (18:2) levels	Inverse variance weighted	21	2.383499e−02	0.93 (0.87−0.99)
1−palmitoyl−GPG (16:0) levels	Inverse variance weighted	28	2.407953e−02	0.93 (0.87−0.99)
5−hydroxyindole sulfate levels	Inverse variance weighted	20	2.615751e−02	0.93 (0.87−0.99)
Thymol sulfate levels	Inverse variance weighted	20	3.124574e−02	0.93 (0.87−0.99)
Benzoate to linoleoyl−arachidonoyl−glycerol (18:2 to 20:4) [1] ratio	Inverse variance weighted	20	3.955971e−02	0.93 (0.86−1.00)
Dihomo−linolenate (20:3n3 or n6) levels	Inverse variance weighted	21	4.082183e−02	0.93 (0.88−1.00)
X−21821 levels	Inverse variance weighted	18	4.462825e−02	0.93 (0.87−1.00)
Cysteinylglycine levels	Inverse variance weighted	23	4.126105e−03	0.92 (0.86−0.97)
X−17690 levels	Inverse variance weighted	35	7.043086e−03	0.92 (0.87−0.98)
4−hydroxy−2−oxoglutaric acid levels	Inverse variance weighted	27	1.043929e−02	0.92 (0.87−0.98)
Alpha−ketobutyrate to 3−methyl−2−oxobutyrate ratio	Inverse variance weighted	22	1.143351e−02	0.92 (0.86−0.98)
Taurine to cysteine ratio	Inverse variance weighted	20	2.991660e−02	0.92 (0.85−0.99)
1−palmitoyl−2−dihomo−linolenoyl−GPC (16:0/20:3n3 or 6) levels	Inverse variance weighted	34	3.446250e−04	0.91 (0.87−0.96)
Stachydrine levels	Inverse variance weighted	21	1.312748e−02	0.91 (0.84−0.98)
4−methyl−2−oxopentanoate to 3−methyl−2−oxobutyrate ratio	Inverse variance weighted	20	2.051319e−02	0.91 (0.84−0.99)
Phosphate to N−palmitoyl−sphingosine (d18:1 to 16:0) ratio	Inverse variance weighted	25	2.447439e−02	0.91 (0.83−0.99)
X−12680 levels	Inverse variance weighted	15	2.823669e−02	0.91 (0.84−0.99)
1−linoleoylglycerol (18:2) levels	Inverse variance weighted	17	4.170736e−02	0.91 (0.84−1.00)
Docosatrienoate (22:3n3) levels	Inverse variance weighted	27	2.920000e−06	0.90 (0.86−0.94)
X−11632 levels	Inverse variance weighted	22	1.474040e−03	0.90 (0.85−0.96)
X−25790 levels	Inverse variance weighted	14	2.293442e−02	0.90 (0.82−0.99)
3−hydroxyhexanoate levels	Inverse variance weighted	20	1.825751e−03	0.89 (0.83−0.96)

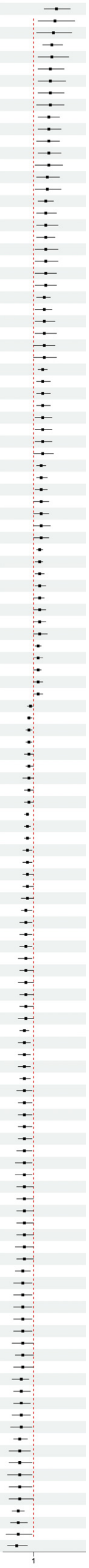

Figure S13. Mendelian randomization estimates of the causal associations between blood metabolites and RA. The forest plot shows the statistically significant results of IVW

Mendelian randomization analyses evaluating the associations between 1400 blood metabolites and the risk of RA. Each row represents a specific blood metabolite, with ORs and 95% CIs plotted. ORs greater than 1 indicate increased risk, and ORs less than 1 indicate a protective effect. RA, rheumatoid arthritis; IVW, inverse-variance weighted; OR, odds ratio; CI, confidence intervals.

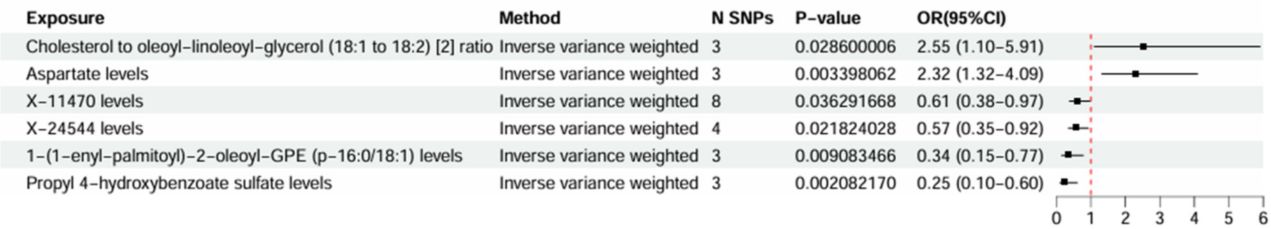

Figure S14. Mendelian randomization estimates of the causal associations between blood metabolites and JIA. The forest plot shows the statistically significant results of IVW Mendelian randomization analyses evaluating the associations between 1400 blood metabolites and the risk of JIA. Each row represents a specific blood metabolite, with ORs and 95% CIs plotted. ORs greater than 1 indicate increased risk, and ORs less than 1 indicate a protective effect. JIA, juvenile idiopathic arthritis; IVW, inverse-variance weighted; OR, odds ratio; CI, confidence intervals.

2. Supplementary Tables

Table S1. Age-standardized rates of RA across 204 countries and territories in 2021, including EAPCs of age-standardized rates from 1990 to 2021. RA, rheumatoid arthritis; EAPC, estimated annual percentage change.

Table S2. Frontier analysis results of RA based on SDI and age-standardized DALY rate per 100,000 population. RA, rheumatoid arthritis; SDI, socio-demographic index; DALYs, disability-adjusted life years.

Table S3. MR estimates of the causal effects of immune cells, circulating inflammatory proteins, and blood metabolites on RA. The table presents statistically significant associations between genetically predicted immune cells, circulating inflammatory proteins, blood metabolites, and the risk of RA based on two-sample MR analyses. Results are shown across five MR methods: IVW, MR Egger, weighted median, weighted mode, and simple mode. The number of instrumental SNPs, OR, 95% CI, and associated P values are provided for each trait. Heterogeneity and Egger intercept P values are included to evaluate instrument validity and potential directional pleiotropy.MR, Mendelian randomization; RA, rheumatoid arthritis; SNP, single nucleotide polymorphism; IVW, inverse-variance weighted; OR, odds ratio; CI, confidence intervals.

Table S4. MR estimates of the causal effects of immune cells, circulating inflammatory proteins, and blood metabolites on JIA. The table presents statistically significant associations between genetically predicted immune cells, circulating inflammatory proteins, blood metabolites, and the risk of JIA based on two-sample MR analyses. Results are shown across five MR methods: IVW, MR Egger, weighted median, weighted mode, and simple mode. The number of instrumental SNPs, OR, 95% CI, and associated P values are provided for each trait. Heterogeneity and Egger intercept P values are included to evaluate instrument validity and potential directional pleiotropy.MR, Mendelian randomization; JIA, juvenile idiopathic arthritis; SNP, single nucleotide polymorphism; IVW, inverse-variance weighted; OR, odds ratio; CI, confidence intervals.
