# Supplementary material for: Global burden and genetic insights of RA and JIA in ages 0–19 years: GBD 2021 and MR analysis
Source: Front Immunol. 2026 Jan 14;16:1661461. doi: 10.3389/fimmu.2025.1661461 (PMC12847325; doi:10.3389/fimmu.2025.1661461)
Supplement: Supplementary file 2 [file DataSheet2.pdf]

Table S1

Location	ASIR	EAPC of ASIR	ASPR	EAPC of ASPR	ASDR	EAPC of ASDR
Afghanistan	1.491624928	0.730517597	6.57203	0.640778432	2.33719079	1.336828775
Albania	1.905341728	1.239462683	8.3092	1.100548565	1.470411435	-1.268769303
Algeria	1.401880964	1.42205939	6.05893	1.2781333	1.15476679	1.106099284
American Samoa	2.009820339	0.575912047	9.33374	0.534201833	1.370110093	0.567344587
Andorra	3.289711258	0.516147511	16.7552	0.546960962	2.465034281	0.472421768
Angola	0.950955209	0.587322289	4.33657	0.50282054	0.665976441	0.2179798
Antigua and Barbuda	1.691849498	0.845337803	7.28885	0.757716055	1.43673558	0.410003495
Argentina	2.792743091	1.062512142	13.1414	1.005743018	2.336241102	0.504400247
Armenia	1.634089594	1.097622643	6.97556	0.990559446	1.051411277	1.165662263
Australia	2.86704522	1.145701128	13.5133	1.131684514	2.068752544	0.672726246
Austria	3.464878036	0.672371585	17.2425	0.683407577	2.622276807	0.244161111
Azerbaijan	1.568422428	1.020126678	6.83773	0.938988057	1.042802948	0.961403834
Bahamas	1.974199392	0.836370406	8.39261	0.762446031	1.688615132	0.276493726
Bahrain	3.050130006	1.118056086	12.3915	0.988151964	2.227281209	1.905954104
Bangladesh	2.110781099	1.431544394	8.70458	1.309446864	1.335812659	0.990834034
Barbados	2.184845174	0.681910212	9.19965	0.59980239	2.069627502	0.585216824
Belarus	1.347060083	0.819587576	5.81537	0.715593295	0.889886142	0.448506631
Belgium	3.342553511	0.649453624	16.6818	0.657402494	2.616150169	0.206238532
Belize	2.114666213	1.09922995	8.96797	1.005472741	1.782628873	0.059181207
Benin	0.976229992	0.904777861	4.49065	0.815182125	0.660420166	0.814819307
Bermuda	2.188658354	0.917297441	9.26283	0.837243357	1.530401435	0.372241878
Bhutan	2.663450482	1.497084111	11.0276	1.374454195	1.681895106	1.190972817
Bolivia (Plurinational State of)	3.904366006	1.193584332	16.113	1.064079506	2.838427637	-0.191510553
Bosnia and Herzegovina	2.700908347	1.138390719	11.414	1.019120033	1.873219769	0.104167044
Botswana	1.765797896	0.901779208	7.28262	0.83395519	1.41155387	0.004229465
Brazil	4.023910618	0.45047971	16.554	0.433469638	3.099403524	0.259141494
Brunei Darussalam	2.169074158	0.793453193	9.99646	0.823443197	1.760037383	-0.033151208
Bulgaria	1.81374319	0.492457796	8.02158	0.441454449	1.250747145	-0.31265418
Burkina Faso	0.876918766	0.875561385	4.06682	0.800655749	0.598288521	0.800408656
Burundi	0.732033163	0.210346638	3.42133	0.161297305	0.529587428	-0.128741908
Cabo Verde	1.034992459	1.106728042	4.69515	0.997038805	0.687524951	0.976560531
Cambodia	0.993583522	0.794718739	4.97467	0.734287878	1.08589943	-0.490344116
Cameroon	1.116715389	0.593011852	5.11931	0.529410772	0.746664286	0.491163311
Canada	3.286541242	0.975273125	16.458	1.016674993	2.543018518	0.703849503
Central African Republic	0.859409562	0.256287305	3.96752	0.206738947	0.625362198	-0.037530169
Chad	0.735575596	0.479661948	3.45655	0.454832311	0.508565097	0.454134286
Chile	3.338740542	1.260135556	15.455	1.164624561	2.660473957	-0.048637527
China	2.861508709	1.026960091	12.0352	1.00322466	2.152580576	-0.373059017
Colombia	1.977798617	0.9536057	8.2164	0.847821845	1.453076953	-0.264664955
Comoros	0.87130228	0.41521403	4.00276	0.359643394	0.61230684	0.145991935
Congo	1.104866516	0.558104072	4.95637	0.476341244	0.752301222	0.238435611
Cook Islands	1.788548183	1.129809025	8.37555	1.051342652	1.223768416	1.026275845
Costa Rica	2.104595132	1.036344512	8.69564	0.940427728	1.466848862	0.497005705
Coted'Ivoire	0.954802838	0.691548937	4.38161	0.607856419	0.644586626	0.60798425
Croatia	2.183275639	1.048441768	9.36295	0.941087652	1.501201435	-0.586925404
Cuba	2.26015983	1.067326385	9.38797	0.976188526	1.805767445	0.171266857
Cyprus	3.128899682	0.661809461	15.4523	0.65839534	2.405067063	-0.160908622
Czechia	2.793784516	0.860200246	11.9774	0.783416899	1.815278836	0.103883394
Democratic People's Republic of Korea	1.873098426	0.785018527	8.70908	0.77545526	1.977412987	0.122697948
Democratic Republic of the Congo	0.852510233	0.092647929	3.90859	0.033579294	0.601805338	-0.151874844
Denmark	3.008605449	1.259351432	15.5019	1.198787848	2.437717606	0.508620058
Djibouti	0.797019226	0.586461492	3.68495	0.528088567	0.558122934	0.352798138
Dominica	1.997887078	0.971337061	8.61656	0.909443241	2.029703511	0.662425243
Dominican Republic	1.468147548	0.975065322	6.4962	0.889883139	1.471330509	-0.238470363
Ecuador	3.379776352	1.12772525	13.7572	1.016388119	2.316339632	-1.407385457
Egypt	2.462083235	1.360496693	10.4125	1.202922653	1.687715159	1.002798359

El Salvador	1.796697065	1.053328771	7.63264	0.935223738	1.215813061	0.581117866
Equatorial Guinea	1.162410344	1.508140087	5.22336	1.36499916	0.783527703	0.874109211
Eritrea	0.674033691	0.322849946	3.13363	0.282301438	0.484881331	0.081631853
Estonia	1.90048999	0.845477497	7.84049	0.753655083	2.52113545	-2.026630572
Eswatini	1.950492397	0.291365159	8.02998	0.272290626	1.717312859	-0.367506707
Ethiopia	0.764440837	0.211791399	3.47239	0.172044921	0.530590016	-0.15507307
Fiji	1.357845963	0.880174417	6.58263	0.867501596	0.964708727	0.846891922
Finland	3.856447108	0.798593203	19.2823	0.776685333	3.63405177	-0.13706987
France	2.338777264	0.896678354	11.6179	0.882749501	1.844010945	0.204556129
Gabon	1.326862066	1.120837045	5.90906	1.009488297	0.883287079	0.769517825
Gambia	0.924450482	0.798117429	4.24215	0.728723914	0.623985457	0.728718113
Georgia	1.589256353	0.415143781	6.82106	0.372548047	1.011539459	0.429588082
Germany	3.490478905	0.666461065	17.5158	0.684979154	2.66102232	0.395363006
Ghana	1.008444965	1.235168295	4.61043	1.144877458	0.674748399	1.141526596
Greece	3.639398257	0.593732893	17.9773	0.623407486	2.685959455	0.519211334
Greenland	3.193554124	1.281696172	18.7788	1.308399613	2.732384947	1.307608302
Grenada	1.605601417	1.086094413	6.96786	0.977641581	1.161937963	0.468014015
Guam	1.737872386	1.022767477	8.11395	0.958060896	1.186833621	0.93963475
Guatemala	1.738256077	1.050612709	7.32041	0.917405334	1.456423911	-0.909827625
Guinea	0.838081817	0.492452799	3.91229	0.448785388	0.575435371	0.44909396
Guinea-Bissau	0.834231156	0.464134382	3.8938	0.39592112	0.572754747	0.395353145
Guyana	1.50810091	0.896350898	6.67719	0.817651764	1.252822266	1.453459759
Haiti	1.290014437	0.353929249	5.78415	0.307368573	2.200890668	-0.59576732
Honduras	1.980548933	0.747962273	8.14296	0.661390231	1.675024391	-1.178562746
Hungary	1.902913999	0.836961268	8.17205	0.782832206	1.324885845	-0.914674641
Iceland	3.773034172	0.693721525	18.7025	0.687845966	2.818077861	0.238516467
India	1.882134183	0.782454465	7.75422	0.699938014	1.17199283	0.395746902
Indonesia	0.768885979	0.775881243	3.73941	0.753847262	0.699719053	0.200202006
Iran (Islamic Republic of)	1.800622104	1.202015565	7.62783	1.083041354	1.321391816	0.881629096
Iraq	2.233914736	0.937137288	9.49367	0.797567474	1.474540969	0.348549528
Ireland	3.824089545	0.745097863	18.8361	0.733955765	3.074309497	-0.327816427
Israel	3.295529726	0.678290285	16.632	0.674562936	2.511728804	0.438392391
Italy	2.36801959	0.126023503	10.4433	0.147294588	1.825346128	-0.621901612
Jamaica	1.730053169	0.93225499	7.47273	0.8422077	1.50664393	0.517277161
Japan	2.189631733	0.782402066	9.29598	0.710727658	1.621502975	-0.461782448
Jordan	2.554195292	1.599215037	10.7833	1.438115581	1.662582474	1.310169627
Kazakhstan	1.721825027	0.962973798	7.3422	0.863907266	1.231845709	1.112583019
Kenya	0.845692596	0.461174045	3.7957	0.425024799	0.574494898	0.335612734
Kiribati	1.282979314	0.473589289	6.27724	0.442475957	0.925452556	0.435638477
Kuwait	6.362721092	1.456071634	24.036	1.349155502	3.655428557	1.28670216
Kyrgyzstan	2.254478289	0.597641826	8.92341	0.532762885	1.608450335	0.832145994
Lao People's Democratic Republic	1.05193219	0.873754216	5.27355	0.799843423	1.156071236	-0.537948285
Latvia	1.573131627	0.722091223	6.64995	0.635573499	1.620553973	-1.932483178
Lebanon	3.687920649	1.114606792	14.6794	0.998246776	2.229081877	0.509612097
Lesotho	1.67370038	0.590974685	6.97005	0.554558176	1.546712603	0.659383685
Liberia	1.034474497	0.913442999	4.76161	0.80112805	0.689003146	0.760468199
Libya	2.25919454	1.041618419	9.707	0.925425033	1.771481579	1.26209554
Lithuania	2.132246282	0.537417266	8.73762	0.453474028	2.486095906	-1.296096502
Luxembourg	3.251626637	0.504334433	16.3322	0.516087272	2.467083309	0.079086879
Madagascar	0.722521442	0.275368258	3.34196	0.216082178	0.512547389	0.036401738
Malawi	0.881455447	0.504819081	4.06013	0.431617783	0.621268714	0.19429535
Malaysia	0.849325191	1.008970171	4.30176	1.00057798	0.663918013	0.699050919
Maldives	1.30965834	0.748363841	6.37406	0.700312024	1.145920884	-1.105624937
Mali	0.81740461	0.720850442	3.79101	0.667261385	0.557910544	0.667430099
Malta	3.340771697	0.689579825	16.7283	0.703853023	2.658482564	0.433964549
Marshall Islands	1.321345746	0.842416258	6.2541	0.786007647	0.9294356	0.804313697
Mauritania	1.129792561	0.941917457	5.1201	0.839515179	0.749495613	0.80217863
Mauritius	1.304352854	1.279138916	6.47863	1.236744769	1.241347167	1.400931923

Mexico	2.851481631	0.64856416	11.1413	0.58106334	2.255523354	-0.650293237
Micronesia (Federated States of)	1.604319602	0.904619969	7.49131	0.811539405	1.110303716	0.853113287
Monaco	3.537504905	0.515551986	18.1779	0.537239848	2.644667024	0.511603378
Mongolia	1.821839579	0.79410083	7.64132	0.640739014	1.350981914	0.662575656
Montenegro	2.043438047	0.773226477	8.91095	0.697765859	1.578049665	-0.217847253
Morocco	1.999131812	1.219177379	8.64432	1.098242154	1.782914952	0.94731753
Mozambique	0.8204186	0.541123561	3.81138	0.496951804	0.592490405	0.28706442
Myanmar	1.079868855	1.017245149	5.38203	0.954746726	1.151774652	-0.558354276
Namibia	1.484397275	0.434401029	6.18672	0.387219171	1.247849875	-0.333223117
Nauru	1.579608742	0.519856535	7.42715	0.485328238	1.103218052	0.531151067
Nepal	2.343155402	1.536413595	9.82591	1.416875951	1.494957329	1.195455729
Netherlands	3.527297028	0.653819847	17.515	0.652767033	2.880653914	-0.12618494
New Zealand	2.547242924	0.982650646	10.7949	0.938167339	1.61881735	0.633890278
Nicaragua	1.960364294	0.803403275	8.13343	0.717129384	1.41331143	-0.368205326
Niger	0.75894732	0.448465312	3.55471	0.392081725	0.523126152	0.391172044
Nigeria	0.959276545	0.707459828	4.31342	0.643182633	0.633017198	0.635598934
Niue	1.683277219	1.060535453	7.9215	0.993050956	1.19165871	0.99964547
North Macedonia	1.80529921	1.01291803	7.98613	0.91813437	1.205011878	0.57522651
Northern Mariana Islands	1.858337122	0.258485138	8.59745	0.266357103	1.412595642	0.518489264
Norway	2.876819453	0.665586045	12.5189	0.640111018	1.906097452	-0.423162089
Oman	2.38755027	1.991786396	10.1846	1.822244794	1.577518548	1.743206906
Pakistan	3.608735882	0.274928118	14.391	0.241411303	2.178838569	0.224317716
Palau	1.798465064	0.875477291	8.32981	0.821100309	1.212073819	0.781094166
Palestine	2.053872333	0.885400408	8.72253	0.785561504	1.418089223	0.341718672
Panama	1.749886138	0.973123236	7.34843	0.890414846	1.243187961	0.462449835
Papua New Guinea	1.045318483	0.482351897	5.12955	0.467850905	0.771536201	0.515694341
Paraguay	3.775421078	1.049980401	15.4728	0.92972295	4.418857877	0.776756828
Peru	9.636175766	1.475497962	36.5046	1.34298084	5.479007796	0.82369765
Philippines	1.461890918	-0.154126319	6.77676	-0.285823601	1.386467747	-1.075457204
Poland	2.363117095	0.550803457	9.75491	0.495532677	1.799128604	-1.987277927
Portugal	3.462557356	1.12718227	16.5567	1.066330639	2.561851258	0.053849471
Puerto Rico	2.465362977	1.156082846	10.2555	1.036095445	1.667960274	0.095511825
Qatar	3.370414498	1.504820088	13.8068	1.315025154	2.086700939	1.164394398
Republic of Korea	2.255259166	0.503367688	10.2394	0.52049101	1.594771378	-0.818553808
Republic of Moldova	1.449503715	1.02021623	6.26022	0.90996543	1.354766826	-0.25365791
Romania	1.804030343	1.11371078	7.98213	1.01198028	1.164784047	1.004953604
Russian Federation	2.080777787	0.825982764	8.38152	0.740138028	2.068423718	-0.884650806
Rwanda	0.863037959	0.49998188	3.97467	0.431710942	0.603961234	-0.029196355
Saint Kitts and Nevis	1.946930752	0.916406057	8.28969	0.82460562	2.120917917	-0.02825664
Saint Lucia	1.907904679	0.948161711	8.1486	0.850757564	1.914367945	0.374426029
Saint Vincent and the Grenadines	1.614351996	1.102529743	7.06158	1.017537992	1.477430528	0.762231101
Samoa	1.589845715	0.48621513	7.44113	0.423737377	1.09124153	0.422462895
San Marino	3.255313416	0.469387688	16.7137	0.509789341	2.470737565	0.400435582
Sao Tome and Principe	1.052533734	0.849551773	4.79521	0.763147281	0.704390561	0.760056334
Saudi Arabia	2.474842103	1.672258155	10.5389	1.501793903	1.656206872	1.189228956
Senegal	0.895783431	0.447312738	4.12628	0.385913084	0.607068681	0.383659331
Serbia	1.526610139	0.921390005	6.70816	0.819819476	1.06943844	-0.627642418
Seychelles	1.277277451	0.91285005	6.37323	0.881640253	0.940730027	0.836127713
Sierra Leone	0.845715659	0.448068897	3.93251	0.402873229	0.578205877	0.404635386
Singapore	2.081797657	1.024064808	9.7428	1.059462629	1.446928958	0.868796759
Slovakia	1.846862798	0.856706674	8.14184	0.770452944	1.312175516	-0.013486095
Slovenia	2.127817637	1.041304897	9.22804	0.9309593	1.560447834	-1.500632983
Solomon Islands	1.332470453	0.817239479	6.30469	0.764115257	0.93546876	0.785266552
Somalia	0.729002118	0.128723747	3.42077	0.104033792	0.538590566	-0.10469639
South Africa	3.138928346	-0.071365239	11.942	-0.045353444	2.318994996	-1.580621064
South Sudan	0.844197362	0.544560017	3.903	0.487642755	0.603866358	0.372742861
Spain	2.197825494	0.532490571	11.1818	0.6553276	1.761332423	0.012891827
Sri Lanka	1.009222871	0.867521203	5.05643	0.810578458	0.777098037	0.555663187

Sudan	1.736695831	1.388922068	7.63319	1.24378608	1.690255083	1.2041656
Suriname	1.479426667	0.871806604	6.57728	0.798102621	1.118221595	0.398318243
Sweden	3.111708684	-0.271649559	14.7576	-0.158686447	2.222031429	-0.479003461
Switzerland	3.234388651	0.537914621	16.071	0.550318236	2.493108759	0.11669356
Syrian Arab Republic	2.176040044	1.316321663	9.27353	1.190632296	1.884947913	0.921086863
Taiwan (Province of China)	1.529850152	0.618037018	8.33326	0.831175923	1.396565675	-0.122674917
Tajikistan	2.216861765	0.389524928	8.63463	0.353534299	1.422544119	0.585729278
Thailand	1.349914207	1.300556206	6.58677	1.181965166	1.201857418	-1.120057698
Timor-Leste	0.894233971	0.695220271	4.50429	0.628315158	0.981511391	-0.086627388
Togo	0.8599899	0.578251925	3.97023	0.500891265	0.584199167	0.503082914
Tokelau	1.475398289	1.153329565	6.94444	1.09345394	1.05680998	1.117221388
Tonga	1.651883228	0.7458999	7.76973	0.692324849	1.154508854	0.702093828
Trinidad and Tobago	1.986774983	1.038715305	8.4228	0.960508155	2.245579687	-0.300871225
Tunisia	2.129895047	1.254751812	9.13636	1.137182883	1.512551344	1.021355498
Turkey	3.107601746	1.151170921	13.0602	1.029992111	2.649591515	-0.872388882
Turkmenistan	1.607218972	1.132172542	6.93409	1.032174177	1.09348103	1.294799556
Tuvalu	1.392863379	0.970712867	6.60321	0.905580027	0.977456618	0.887361006
Uganda	0.828553357	0.632331511	3.83223	0.577900364	0.585348351	0.425663922
Ukraine	1.295068167	0.631190222	5.51816	0.56028543	1.529152382	-0.644848231
United Arab Emirates	3.250927917	1.775421284	13.4461	1.638935313	2.122514319	0.415952815
United Kingdom	5.561713461	0.348119751	29.084	0.394610609	4.326235113	0.097150365
United Republic of Tanzania	0.868860883	0.532529031	4.01622	0.48223847	0.612699941	0.299619913
United States of America	4.040338885	0.98001718	26.4302	0.978389273	3.935065885	0.773991245
United States Virgin Islands	2.061311614	0.983554708	8.88499	0.911347688	1.354238386	0.348090305
Uruguay	2.614148009	1.041416783	12.4467	0.989614113	2.587495235	0.210074065
Uzbekistan	2.863814118	0.753418432	11.2156	0.688407865	1.891039073	1.359387248
Vanuatu	1.274069941	0.768974308	6.10728	0.742185592	0.902239577	0.750813676
Venezuela (Bolivarian Republic of)	2.317947923	0.970791201	9.47389	0.853321203	2.118566326	0.743376623
Viet Nam	1.464493022	1.051108748	6.93998	1.013520929	1.168526319	0.61670517
Yemen	1.303978875	0.887312062	5.81355	0.798992829	1.196007508	1.194270058
Zambia	0.914287286	0.424514924	4.19481	0.366710192	0.638946881	0.118234302
Zimbabwe	1.372522752	-0.035417489	5.7944	-0.028384328	1.364657173	0.687652116
