# Supplementary material for: Global burden and genetic insights of RA and JIA in ages 0–19 years: GBD 2021 and MR analysis
Source: Front Immunol. 2026 Jan 14;16:1661461. doi: 10.3389/fimmu.2025.1661461 (PMC12847325; doi:10.3389/fimmu.2025.1661461)
Supplement: Supplementary file 3 [file DataSheet3.pdf]

Table S2

Location	SDI	Rate of DALYs	Frontier DALYs	Effective difference	Effective difference rank (Age-standardized DALYs rank)
Somalia	0.07768811	0.54(0.25 to 1.05)	0.54	0	1 (7)
Eritrea	0.40386394	0.48(0.23 to 0.95)	0.45	0.04	2 (1)
Chad	0.24043602	0.51(0.23 to 0.91)	0.45	0.06	3 (2)
Madagascar	0.40024694	0.51(0.23 to 0.97)	0.45	0.07	4 (3)
Burundi	0.28937437	0.53(0.24 to 1.01)	0.45	0.08	6 (5)
Ethiopia	0.3588233	0.53(0.25 to 0.99)	0.45	0.08	7 (6)
Niger	0.16807277	0.52(0.25 to 0.94)	0.45	0.08	5 (4)
Djibouti	0.48795837	0.56(0.26 to 1.03)	0.45	0.11	8 (9)
Mali	0.26857994	0.56(0.26 to 0.99)	0.45	0.11	9 (8)
Guinea	0.33640129	0.58(0.27 to 1.01)	0.45	0.13	12 (12)
Guinea-Bissau	0.35310962	0.57(0.28 to 1.03)	0.45	0.13	10 (10)
Kenya	0.52376808	0.57(0.27 to 1.05)	0.45	0.13	11 (11)
Sierra Leone	0.35866588	0.58(0.27 to 1.03)	0.45	0.13	13 (13)
Mozambique	0.32646261	0.59(0.28 to 1.14)	0.45	0.14	16 (16)
Togo	0.4085337	0.58(0.27 to 1.03)	0.45	0.14	14 (14)
Uganda	0.42326118	0.59(0.27 to 1.07)	0.45	0.14	15 (15)
Burkina Faso	0.2851184	0.6(0.29 to 1.07)	0.45	0.15	17 (17)
Democratic Republic of the Congo	0.38317985	0.6(0.28 to 1.14)	0.45	0.15	18 (18)
Rwanda	0.43558871	0.6(0.28 to 1.1)	0.45	0.16	19 (20)
Senegal	0.40805419	0.61(0.29 to 1.09)	0.45	0.16	21 (21)
South Sudan	0.27837113	0.6(0.29 to 1.15)	0.45	0.16	20 (19)
Comoros	0.47597869	0.61(0.28 to 1.14)	0.45	0.17	23 (22)
Malawi	0.38455363	0.62(0.29 to 1.15)	0.45	0.17	24 (24)
United Republic of Tanzania	0.44656827	0.61(0.29 to 1.13)	0.45	0.17	22 (23)
Central African Republic	0.30916769	0.63(0.3 to 1.23)	0.45	0.18	26 (26)
Gambia	0.40971416	0.62(0.31 to 1.1)	0.45	0.18	25 (25)
Nigeria	0.50339083	0.63(0.31 to 1.11)	0.45	0.19	27 (27)
Zambia	0.50594895	0.64(0.31 to 1.16)	0.45	0.19	28 (28)
Coted'Ivoire	0.42594188	0.64(0.31 to 1.14)	0.45	0.2	29 (29)
Benin	0.37348657	0.66(0.31 to 1.17)	0.45	0.21	30 (30)
Angola	0.45372195	0.67(0.32 to 1.27)	0.45	0.22	32 (32)
Malaysia	0.74252383	0.66(0.33 to 1.19)	0.45	0.22	31 (31)
Ghana	0.56493039	0.67(0.29 to 1.24)	0.45	0.23	33 (33)
Cabo Verde	0.53353454	0.69(0.3 to 1.3)	0.45	0.24	34 (34)
Liberia	0.35244245	0.69(0.28 to 1.24)	0.45	0.24	35 (35)
Indonesia	0.65686834	0.7(0.4 to 1.12)	0.45	0.25	36 (36)
Sao Tome and Principe	0.50541375	0.7(0.29 to 1.3)	0.45	0.26	37 (37)
Cameroon	0.47969122	0.75(0.35 to 1.35)	0.45	0.3	38 (38)
Congo	0.58307524	0.75(0.34 to 1.51)	0.45	0.3	40 (40)
Mauritania	0.4989451	0.75(0.33 to 1.38)	0.45	0.3	39 (39)
Papua New Guinea	0.41779744	0.77(0.4 to 1.33)	0.45	0.32	41 (41)
Sri Lanka	0.70153494	0.78(0.41 to 1.34)	0.45	0.33	42 (42)
Equatorial Guinea	0.65785746	0.78(0.35 to 1.46)	0.45	0.34	43 (43)
Belarus	0.78448471	0.89(0.42 to 1.59)	0.45	0.44	45 (45)
Gabon	0.63469139	0.88(0.39 to 1.66)	0.45	0.44	44 (44)
Vanuatu	0.47310071	0.9(0.45 to 1.61)	0.45	0.45	46 (46)
Kiribati	0.52718658	0.93(0.44 to 1.66)	0.45	0.48	47 (47)
Marshall Islands	0.57409113	0.93(0.45 to 1.61)	0.45	0.48	48 (48)
Seychelles	0.73015078	0.94(0.49 to 1.65)	0.45	0.49	50 (50)
Solomon Islands	0.42936032	0.94(0.46 to 1.65)	0.45	0.49	49 (49)
Fiji	0.67505163	0.96(0.48 to 1.74)	0.45	0.52	51 (51)
Timor-Leste	0.44466762	0.98(0.59 to 1.51)	0.45	0.53	53 (53)
Tuvalu	0.57662053	0.98(0.49 to 1.67)	0.45	0.53	52 (52)
Georgia	0.7324736	1.01(0.49 to 1.82)	0.45	0.56	54 (54)
Armenia	0.70183319	1.05(0.52 to 1.89)	0.45	0.6	56 (56)
Azerbaijan	0.69485127	1.04(0.51 to 1.83)	0.45	0.6	55 (55)
Tokelau	0.68642562	1.06(0.54 to 1.86)	0.45	0.61	57 (57)
Serbia	0.79241629	1.07(0.55 to 1.82)	0.45	0.62	58 (58)
Cambodia	0.47362149	1.09(0.65 to 1.66)	0.45	0.64	59 (59)
Samoa	0.59339277	1.09(0.53 to 1.93)	0.45	0.64	60 (60)
Turkmenistan	0.68216078	1.09(0.57 to 1.86)	0.45	0.65	61 (61)
Micronesia (Federated States of)	0.58753497	1.11(0.54 to 1.99)	0.45	0.66	63 (63)
Nauru	0.62517783	1.1(0.52 to 1.96)	0.45	0.66	62 (62)
Suriname	0.63366574	1.12(0.6 to 1.91)	0.45	0.67	64 (64)
Maldives	0.65088663	1.15(0.62 to 1.92)	0.45	0.7	65 (65)
Myanmar	0.53390084	1.15(0.72 to 1.79)	0.45	0.7	66 (66)
Algeria	0.65950092	1.15(0.63 to 1.91)	0.45	0.71	68 (68)
Grenada	0.66899303	1.16(0.64 to 1.97)	0.45	0.71	70 (70)
Lao People's Democratic Republic	0.48913609	1.16(0.71 to 1.78)	0.45	0.71	69 (69)
Tonga	0.62634994	1.15(0.53 to 2.08)	0.45	0.71	67 (67)
India	0.57540165	1.17(0.64 to 1.94)	0.45	0.72	73 (73)
Romania	0.76845386	1.16(0.59 to 2.02)	0.45	0.72	71 (71)
Viet Nam	0.62793372	1.17(0.62 to 1.98)	0.45	0.72	72 (72)
Guam	0.8039822	1.19(0.59 to 2.1)	0.45	0.74	74 (74)
Niue	0.72622205	1.19(0.59 to 2.08)	0.45	0.74	75 (75)
Yemen	0.45037638	1.2(0.64 to 1.93)	0.45	0.75	76 (76)
North Macedonia	0.7506297	1.21(0.6 to 2.21)	0.45	0.76	78 (78)
Palau	0.75404693	1.21(0.57 to 2.13)	0.45	0.76	79 (79)
Thailand	0.68254793	1.2(0.66 to 2)	0.45	0.76	77 (77)
El Salvador	0.56377519	1.22(0.63 to 2.06)	0.45	0.77	80 (80)
Cook Islands	0.77910996	1.22(0.6 to 2.18)	0.45	0.78	81 (81)
Kazakhstan	0.7251445	1.23(0.67 to 2.06)	0.45	0.78	82 (82)
Mauritius	0.71826045	1.24(0.77 to 1.94)	0.45	0.79	83 (83)
Bulgaria	0.76815094	1.25(0.66 to 2.18)	0.45	0.8	86 (86)
Namibia	0.61756487	1.25(0.65 to 2.04)	0.45	0.8	85 (85)
Panama	0.70886483	1.24(0.68 to 2.09)	0.45	0.8	84 (84)
Guyana	0.65081234	1.25(0.7 to 2.04)	0.45	0.81	87 (87)
Slovakia	0.81061053	1.31(0.71 to 2.27)	0.45	0.86	88 (88)

Iran (Islamic Republic of)	0.6972074	1.32(0.79 to 2.1)	0.45	0.87	89 (89)
Hungary	0.79075477	1.32(0.7 to 2.29)	0.45	0.88	90 (90)
Bangladesh	0.49242089	1.34(0.68 to 2.26)	0.45	0.89	91 (91)
Mongolia	0.61762157	1.35(0.76 to 2.25)	0.45	0.9	92 (92)
Republic of Moldova	0.73221488	1.35(0.82 to 2.08)	0.45	0.91	94 (94)
United States Virgin Islands	0.82183085	1.35(0.7 to 2.38)	0.45	0.91	93 (93)
American Samoa	0.72372753	1.37(0.67 to 2.38)	0.45	0.92	96 (96)
Zimbabwe	0.47381949	1.36(0.8 to 2.17)	0.45	0.92	95 (95)
Philippines	0.65121933	1.39(0.88 to 2.13)	0.45	0.94	97 (97)
Taiwan (Province of China)	0.87474705	1.4(0.91 to 2.07)	0.45	0.95	98 (98)
Botswana	0.64272163	1.41(0.76 to 2.37)	0.45	0.96	99 (99)
Northern Mariana Islands	0.77153521	1.41(0.71 to 2.4)	0.45	0.96	100 (100)
Nicaragua	0.52395847	1.41(0.78 to 2.3)	0.45	0.97	101 (101)
Palestine	0.63101167	1.42(0.75 to 2.36)	0.45	0.97	102 (102)
Tajikistan	0.54151119	1.42(0.78 to 2.34)	0.45	0.98	103 (103)
Antigua and Barbuda	0.74988689	1.44(0.86 to 2.31)	0.45	0.99	104 (104)
Singapore	0.85609777	1.45(0.71 to 2.5)	0.45	1	105 (105)
Colombia	0.65544291	1.45(0.79 to 2.41)	0.45	1.01	106 (106)
Guatemala	0.53997242	1.46(0.91 to 2.32)	0.45	1.01	107 (107)
Albania	0.70684979	1.47(0.83 to 2.46)	0.45	1.02	109 (109)
Costa Rica	0.70034048	1.47(0.8 to 2.42)	0.45	1.02	108 (108)
Dominican Republic	0.6193882	1.47(0.87 to 2.36)	0.45	1.02	110 (110)
Iraq	0.66262623	1.47(0.82 to 2.5)	0.45	1.03	111 (111)
Saint Vincent and the Grenadines	0.63719596	1.48(0.88 to 2.38)	0.45	1.03	112 (112)
Croatia	0.79834103	1.5(0.78 to 2.55)	0.45	1.05	114 (114)
Nepal	0.43317464	1.49(0.76 to 2.59)	0.45	1.05	113 (113)
Jamaica	0.68326306	1.51(0.89 to 2.45)	0.45	1.06	115 (115)
Tunisia	0.68243222	1.51(0.81 to 2.46)	0.45	1.06	116 (116)
Bermuda	0.82136542	1.53(0.85 to 2.6)	0.45	1.08	118 (118)
Ukraine	0.76077391	1.53(1.02 to 2.24)	0.45	1.08	117 (117)
Lesotho	0.51039307	1.55(0.86 to 2.4)	0.45	1.1	119 (119)
Slovenia	0.84243073	1.56(0.87 to 2.66)	0.45	1.11	120 (120)
Montenegro	0.79580058	1.58(0.91 to 2.53)	0.45	1.13	122 (122)
Oman	0.7733916	1.58(0.86 to 2.62)	0.45	1.13	121 (121)
Republic of Korea	0.88667527	1.59(0.89 to 2.64)	0.45	1.15	123 (123)
Kyrgyzstan	0.60397933	1.61(0.92 to 2.62)	0.45	1.16	124 (124)
Japan	0.87124181	1.62(0.98 to 2.56)	0.45	1.17	127 (127)
Latvia	0.83066352	1.62(1.07 to 2.46)	0.45	1.17	126 (126)
New Zealand	0.8494425	1.62(0.84 to 2.68)	0.45	1.17	125 (125)
Jordan	0.72530723	1.66(0.94 to 2.77)	0.45	1.21	129 (129)
Saudi Arabia	0.81514349	1.66(0.9 to 2.77)	0.45	1.21	128 (128)
Puerto Rico	0.82552585	1.67(0.9 to 2.85)	0.45	1.22	130 (130)
Bhutan	0.47306238	1.68(0.87 to 2.97)	0.45	1.23	132 (132)
Honduras	0.51303725	1.68(0.96 to 2.73)	0.45	1.23	131 (131)
Bahamas	0.80502067	1.69(1 to 2.73)	0.45	1.24	134 (134)
Egypt	0.60678709	1.69(0.91 to 2.78)	0.45	1.24	133 (133)
Sudan	0.54194974	1.69(0.94 to 2.72)	0.45	1.24	135 (135)
Eswatini	0.58545971	1.72(0.92 to 2.91)	0.45	1.27	136 (136)
Brunei Darussalam	0.81023437	1.76(0.98 to 2.88)	0.45	1.31	137 (137)
Spain	0.7692837	1.76(1.02 to 2.88)	0.45	1.31	138 (138)
Libya	0.7257714	1.77(1.01 to 2.81)	0.45	1.32	139 (139)
Belize	0.610229	1.78(1.1 to 2.77)	0.45	1.34	140 (140)
Morocco	0.5626983	1.78(0.91 to 2.82)	0.45	1.34	141 (141)
Poland	0.81204281	1.8(1.13 to 2.78)	0.45	1.35	142 (142)
Cuba	0.66872986	1.81(1.05 to 2.92)	0.45	1.36	143 (143)
Czechia	0.82845043	1.82(0.97 to 3.05)	0.45	1.37	144 (144)
Italy	0.80577353	1.83(1.11 to 2.88)	0.45	1.38	145 (145)
France	0.83836488	1.84(1.02 to 3.07)	0.45	1.4	146 (146)
Bosnia and Herzegovina	0.72307789	1.87(1.01 to 3.06)	0.45	1.43	147 (147)
Syrian Arab Republic	0.62300408	1.88(1.12 to 2.86)	0.45	1.44	148 (148)
Uzbekistan	0.66262169	1.89(1.07 to 3.05)	0.45	1.44	149 (149)
Norway	0.91613281	1.91(1.04 to 3.14)	0.45	1.46	150 (150)
Saint Lucia	0.67250974	1.91(1.21 to 2.9)	0.45	1.47	151 (151)
Democratic People's Republic of Korea	0.56985463	1.98(1.14 to 3.13)	0.45	1.53	152 (152)
Dominica	0.74696719	2.03(1.18 to 3.2)	0.45	1.58	153 (153)
Australia	0.84425281	2.07(1.03 to 3.56)	0.45	1.62	155 (155)
Barbados	0.74674876	2.07(1.3 to 3.21)	0.45	1.62	156 (156)
Russian Federation	0.80853601	2.07(1.47 to 2.95)	0.45	1.62	154 (154)
Qatar	0.84686058	2.09(0.98 to 3.8)	0.45	1.64	157 (157)
Saint Kitts and Nevis	0.75498706	2.12(1.34 to 3.2)	0.45	1.67	159 (159)
United Arab Emirates	0.84931773	2.12(1.09 to 3.62)	0.45	1.67	160 (160)
Venezuela (Bolivarian Republic of)	0.59651306	2.12(1.36 to 3.26)	0.45	1.67	158 (158)
China	0.72162976	2.15(1.33 to 3.36)	0.45	1.71	161 (161)
Pakistan	0.50402869	2.18(1.22 to 3.47)	0.45	1.73	162 (162)
Haiti	0.44827829	2.2(1.04 to 4.06)	0.45	1.75	163 (163)
Sweden	0.8868803	2.22(1.17 to 3.87)	0.45	1.77	164 (164)
Bahrain	0.7530432	2.23(1.28 to 3.64)	0.45	1.78	165 (165)
Lebanon	0.74474635	2.23(1.08 to 3.93)	0.45	1.78	166 (166)
Trinidad and Tobago	0.76876325	2.25(1.52 to 3.27)	0.45	1.8	167 (167)
Mexico	0.6645753	2.26(1.48 to 3.41)	0.45	1.81	168 (168)
Ecuador	0.66101705	2.32(1.35 to 3.67)	0.45	1.87	169 (169)
South Africa	0.6796266	2.32(1.43 to 3.62)	0.45	1.87	170 (170)
Afghanistan	0.3372	2.34(1.15 to 3.98)	0.45	1.89	172 (172)
Argentina	0.72312297	2.34(1.35 to 3.88)	0.45	1.89	171 (171)
Cyprus	0.83563055	2.41(1.25 to 4.08)	0.45	1.96	173 (173)
Denmark	0.8964242	2.44(1.33 to 4.14)	0.45	1.99	174 (174)
Andorra	0.86944411	2.47(1.22 to 4.23)	0.45	2.02	175 (175)
Luxembourg	0.88442896	2.47(1.27 to 4.15)	0.45	2.02	176 (176)
San Marino	0.88800547	2.47(1.28 to 4.1)	0.45	2.02	177 (177)
Lithuania	0.85648405	2.49(1.73 to 3.58)	0.45	2.04	178 (178)

Switzerland	0.93305911	2.49(1.29 to 4.21)	0.45	2.05	179 (179)
Israel	0.80901165	2.51(1.23 to 4.19)	0.45	2.06	180 (180)
Estonia	0.84491779	2.52(1.82 to 3.49)	0.45	2.07	181 (181)
Canada	0.87317068	2.54(1.52 to 3.8)	0.45	2.1	182 (182)
Portugal	0.74415185	2.56(1.3 to 4.5)	0.45	2.11	183 (183)
Uruguay	0.71928345	2.59(1.63 to 3.94)	0.45	2.14	184 (184)
Austria	0.853837	2.62(1.43 to 4.37)	0.45	2.17	186 (186)
Belgium	0.85365402	2.62(1.43 to 4.38)	0.45	2.17	185 (185)
Monaco	0.90826283	2.64(1.37 to 4.67)	0.45	2.2	187 (187)
Turkey	0.71269267	2.65(1.6 to 3.99)	0.45	2.2	188 (188)
Chile	0.77151472	2.66(1.45 to 4.45)	0.45	2.21	190 (190)
Germany	0.90295709	2.66(1.38 to 4.47)	0.45	2.21	191 (191)
Malta	0.80158503	2.66(1.49 to 4.45)	0.45	2.21	189 (189)
Greece	0.79185441	2.69(1.46 to 4.49)	0.45	2.24	192 (192)
Greenland	0.82621034	2.73(1.38 to 4.65)	0.45	2.29	193 (193)
Iceland	0.87636168	2.82(1.51 to 4.76)	0.45	2.37	194 (194)
Bolivia (Plurinational State of)	0.5990108	2.84(1.57 to 4.6)	0.45	2.39	195 (195)
Netherlands	0.88846426	2.88(1.6 to 4.73)	0.45	2.43	196 (196)
Ireland	0.87375385	3.07(1.67 to 5.04)	0.45	2.63	197 (197)
Brazil	0.65304389	3.1(2.03 to 4.53)	0.45	2.65	198 (198)
Finland	0.85983137	3.63(2.15 to 5.76)	0.45	3.19	199 (199)
Kuwait	0.84665106	3.66(1.78 to 6.28)	0.45	3.21	200 (200)
United States of America	0.86244835	3.94(2.59 to 5.75)	0.45	3.49	201 (201)
United Kingdom	0.85900018	4.33(2.67 to 6.72)	0.45	3.88	202 (202)
Paraguay	0.6357181	4.42(2.79 to 6.57)	0.45	3.97	203 (203)
Peru	0.66205404	5.48(2.97 to 8.92)	0.45	5.03	204 (204)
