# Supplementary material for: Global burden and genetic insights of RA and JIA in ages 0–19 years: GBD 2021 and MR analysis
Source: Front Immunol. 2026 Jan 14;16:1661461. doi: 10.3389/fimmu.2025.1661461 (PMC12847325; doi:10.3389/fimmu.2025.1661461)
Supplement: Supplementary file 4 [file DataSheet4.pdf]

Table S3

			Inverse variance weighted			MR Egger			Weighted median		Weighted mode		Simple mode	
Exposure	N SNPs	Steiger P-value	P-value	OR (95%CI)	Heterogeneity P-value	P-value	OR (95%CI)	Egger intercept P-value	P-value	OR (95%CI)	P-value	OR (95%CI)	P-value	OR (95%CI)
**Immune cells**														
CD4+ AC	13	1.53E-60	0.001169639	1.11 (1.04-1.18)	0.270416872	0.41413455	1.14 (0.85-1.53)	-0.003792484	0.063559412	1.09 (1.00-1.19)	0.130907106	1.12 (0.98-1.29)	0.031530027	1.20 (1.04-1.39)
CX3CR1 on CD14- CD16-	24	2.24E-203	9.10E-05	1.10 (1.05-1.15)	0.008448799	0.08891178	1.16 (0.98-1.37)	-0.010899546	0.001259685	1.09 (1.04-1.16)	0.031077406	1.09 (1.01-1.17)	0.448370367	1.04 (0.94-1.14)
CD14+ CD16- monocyte %monocyte	30	0	3.72E-07	1.09 (1.05-1.12)	0.115798233	0.00108828	1.17 (1.08-1.27)	-0.016420891	0.000964064	1.09 (1.04-1.15)	0.003205188	1.11 (1.04-1.19)	0.069753136	1.10 (1.00-1.22)
DC AC	38	0	6.97E-08	1.08 (1.05-1.10)	0.0947403	0.00167793	1.12 (1.05-1.20)	-0.009522292	2.04E-05	1.10 (1.05-1.14)	0.00093195	1.09 (1.04-1.14)	0.048645358	1.08 (1.00-1.17)
TD DN (CD4-CD8-) AC	21	9.37E-111	0.003564612	1.08 (1.03-1.14)	0.035859632	0.41358001	1.07 (0.91-1.25)	0.002035519	0.05111634	1.07 (1.00-1.14)	0.162727551	1.08 (0.97-1.21)	0.268362795	1.07 (0.95-1.20)
Myeloid DC AC	44	0	3.39E-10	1.07 (1.05-1.10)	0.252907378	2.42E-05	1.12 (1.07-1.18)	-0.011412961	9.22E-07	1.09 (1.05-1.13)	0.000481765	1.09 (1.04-1.13)	0.021174388	1.08 (1.01-1.15)
CD62L- DC AC	50	0	1.54E-07	1.07 (1.05-1.10)	0.009208637	3.06E-05	1.13 (1.07-1.19)	-0.013818585	1.76E-06	1.09 (1.05-1.13)	3.99E-05	1.09 (1.05-1.14)	0.089930557	1.07 (0.99-1.15)
CD62L- DC %DC	47	0	8.71E-07	1.07 (1.04-1.10)	0.007077182	0.00010728	1.13 (1.07-1.19)	-0.0138592	7.10E-05	1.08 (1.04-1.12)	0.00102509	1.07 (1.03-1.11)	0.324228591	1.04 (0.97-1.11)
Naive CD4+ AC	16	1.44E-75	0.010357033	1.07 (1.02-1.12)	0.656230235	0.43892575	1.06 (0.92-1.23)	0.001227421	0.122861053	1.06 (0.98-1.14)	0.452439796	1.05 (0.93-1.18)	0.428819132	1.05 (0.93-1.20)
PB/PC %lymphocyte	14	1.72E-69	0.012959297	1.07 (1.01-1.13)	0.299997202	0.32025388	0.92 (0.78-1.08)	0.025933926	0.038994001	1.09 (1.00-1.18)	0.085298712	1.16 (0.99-1.35)	0.048434242	1.16 (1.01-1.32)
CD28- CD8dim %CD8dim	12	2.01E-64	0.022918072	1.07 (1.01-1.14)	0.136873089	0.58422788	1.09 (0.82-1.44)	-0.001634016	0.171379853	1.07 (0.97-1.17)	0.315351307	1.07 (0.95-1.20)	0.291322233	1.07 (0.95-1.21)
PDL-1 on CD14+ CD16- monocyte	13	8.83E-64	0.034351281	1.07 (1.01-1.14)	0.239958063	0.59873515	1.07 (0.83-1.38)	-0.000374712	0.227893076	1.05 (0.97-1.15)	0.436924898	1.04 (0.94-1.16)	0.729507795	1.02 (0.90-1.17)
CD86+ myeloid DC AC	56	0	2.50E-07	1.06 (1.04-1.08)	0.03338563	0.00021314	1.10 (1.05-1.15)	-0.00932315	1.66E-05	1.07 (1.04-1.10)	0.000138808	1.07 (1.04-1.10)	0.088805278	1.05 (0.99-1.11)
CD80 on myeloid DC	30	2.46E-259	3.78E-05	1.06 (1.03-1.10)	0.272962313	0.00045008	1.16 (1.08-1.25)	-0.01803399	0.008294799	1.06 (1.01-1.11)	0.052553631	1.07 (1.00-1.15)	0.076280516	1.07 (1.00-1.16)
CD25hi CD45RA+ CD4 not Treg AC	30	2.12E-197	0.000173143	1.06 (1.03-1.09)	0.878546447	0.17409949	1.05 (0.98-1.12)	0.002791594	0.053451886	1.04 (1.00-1.09)	0.16656791	1.03 (0.99-1.08)	0.153548632	1.05 (0.98-1.12)
Myeloid DC %DC	40	0	0.000378079	1.06 (1.03-1.09)	0.009481939	0.00205159	1.12 (1.05-1.20)	-0.013610593	9.06E-06	1.10 (1.05-1.14)	0.000658591	1.09 (1.04-1.15)	0.056509148	1.08 (1.00-1.17)
CD127 on CD28+ CD45RA+ CD8br	21	1.59E-109	0.00231715	1.06 (1.02-1.11)	0.320285252	0.98942246	1.00 (0.88-1.13)	0.011861366	0.103712143	1.05 (0.99-1.12)	0.912196625	1.01 (0.88-1.15)	0.934439963	1.01 (0.89-1.13)
CX3CR1 on CD14- CD16+ monocyte	23	2.38E-140	0.003750757	1.06 (1.02-1.10)	0.070029161	0.25345378	1.08 (0.95-1.24)	-0.004433082	0.019368012	1.07 (1.01-1.13)	0.034451073	1.12 (1.01-1.23)	0.086418234	1.10 (0.99-1.22)
TD CD8br %T cell	25	6.42E-164	0.00697978	1.06 (1.01-1.10)	0.625347027	0.70252975	1.03 (0.90-1.17)	0.005238933	0.029863814	1.06 (1.01-1.12)	0.156303878	1.06 (0.98-1.15)	0.180977048	1.06 (0.97-1.16)
CD25hi CD45RA- CD4 not Treg %CD4+	22	4.39E-109	0.011505397	1.06 (1.01-1.11)	0.336509158	0.79764978	1.02 (0.91-1.14)	0.008797322	0.392610898	1.03 (0.96-1.10)	0.898244313	1.01 (0.92-1.11)	0.975576933	1.00 (0.89-1.12)
CD66b++ myeloid cell AC	12	4.45E-61	0.015336105	1.06 (1.01-1.11)	0.872379648	0.89084264	1.01 (0.86-1.19)	0.009952258	0.146728206	1.05 (0.98-1.11)	0.32354193	1.05 (0.96-1.15)	0.232404876	1.06 (0.97-1.17)
CD19 on IgD- CD38br	12	3.64E-58	0.017526659	1.06 (1.01-1.11)	0.416592515	0.1007477	1.08 (0.99-1.18)	-0.006629051	0.064334804	1.07 (1.00-1.14)	0.070952194	1.07 (1.00-1.14)	0.374933737	1.05 (0.95-1.17)
IgD+ AC	11	2.79E-54	0.018149168	1.06 (1.01-1.12)	0.205507823	0.61434835	1.04 (0.90-1.19)	0.005163115	0.02455507	1.08 (1.01-1.16)	0.057054642	1.10 (1.01-1.19)	0.120546201	1.09 (0.99-1.20)
CD127 on CD45RA+ CD4+	26	2.03E-150	0.025236108	1.06 (1.01-1.11)	0.003195866	0.40291626	1.06 (0.93-1.20)	-0.000224599	0.361672909	1.03 (0.97-1.08)	0.76923243	1.01 (0.93-1.10)	0.538771483	1.03 (0.93-1.14)
EM CD4+ %CD4+	17	5.97E-83	0.032475232	1.06 (1.00-1.12)	0.514357063	0.78855742	0.98 (0.84-1.14)	0.013365397	0.214765257	1.05 (0.97-1.13)	0.955886315	1.00 (0.87-1.16)	0.926275524	1.01 (0.88-1.16)
Activated & resting Treg AC	15	7.10E-82	0.039247295	1.06 (1.00-1.12)	0.598223717	0.61965866	1.04 (0.90-1.21)	0.003554337	0.303987571	1.04 (0.96-1.12)	0.576237526	1.03 (0.94-1.13)	0.361452951	1.06 (0.94-1.20)
CD25 on CD39+ activated Treg	13	1.16E-67	0.049050516	1.06 (1.00-1.12)	0.052152591	0.49145209	1.10 (0.84-1.45)	-0.00641296	0.134041256	1.07 (0.98-1.17)	0.360605587	1.07 (0.93-1.24)	0.403290966	1.07 (0.92-1.23)
CD86+ myeloid DC %DC	63	0	8.30E-09	1.05 (1.04-1.07)	0.113190681	0.00125132	1.07 (1.03-1.12)	-0.005177962	3.50E-06	1.07 (1.04-1.10)	0.000352451	1.07 (1.03-1.10)	0.062797198	1.05 (1.00-1.11)
CD62L- CD86+ myeloid DC AC	68	NA	2.18E-08	1.05 (1.03-1.07)	0.121789319	0.00033268	1.07 (1.03-1.11)	-0.007103207	8.67E-06	1.06 (1.03-1.09)	5.31E-05	1.06 (1.03-1.09)	0.018274236	1.06 (1.01-1.11)
CD62L- CD86+ myeloid DC %DC	73	NA	4.28E-06	1.05 (1.03-1.07)	0.002931612	0.00019186	1.08 (1.04-1.12)	-0.009586913	1.87E-05	1.06 (1.03-1.09)	0.000120489	1.06 (1.03-1.09)	0.176141694	1.03 (0.99-1.09)
CD62L- myeloid DC AC	50	0	0.000130068	1.05 (1.03-1.08)	0.017763839	0.00011398	1.11 (1.06-1.17)	-0.014990335	6.84E-06	1.08 (1.05-1.12)	0.000250266	1.07 (1.04-1.11)	0.61555595	1.02 (0.95-1.10)
CD25hi CD45RA+ CD4 not Treg %T cell	31	1.24E-202	0.000376521	1.05 (1.02-1.08)	0.595374063	0.034342	1.06 (1.01-1.12)	-0.003541876	0.078566872	1.04 (1.00-1.09)	0.113842575	1.04 (0.99-1.08)	0.021378133	1.09 (1.02-1.17)
CD28 on CD28+ CD45RA+ CD8br	24	7.65E-256	0.005713352	1.05 (1.02-1.10)	0.020327058	0.19777091	1.07 (0.97-1.17)	-0.002817642	0.009985442	1.06 (1.01-1.11)	0.029078571	1.06 (1.01-1.11)	0.102240938	1.07 (0.99-1.15)
CD25 on memory B cell	33	0	0.00618778	1.05 (1.01-1.08)	0.00625222	0.14555147	1.06 (0.98-1.14)	-0.002621244	0.055342312	1.04 (1.00-1.09)	0.159141527	1.04 (0.98-1.10)	0.398659231	1.03 (0.96-1.12)
CD86 on myeloid DC	27	2.19E-248	0.010120022	1.05 (1.01-1.09)	0.023038263	0.08669953	1.08 (0.99-1.18)	-0.006616807	0.004682665	1.07 (1.02-1.12)	0.038564492	1.07 (1.01-1.14)	0.239391831	1.05 (0.97-1.15)
CD80 on plasmacytoid DC	18	1.40E-120	0.016801786	1.05 (1.01-1.09)	0.431853565	0.10747749	0.89 (0.78-1.02)	0.030086505	0.505591152	1.02 (0.96-1.08)	0.974663585	1.00 (0.92-1.09)	0.835836985	0.99 (0.90-1.08)
CD80 on CD62L+ plasmacytoid DC	18	1.49E-119	0.016866964	1.05 (1.01-1.09)	0.431402808	0.10471712	0.89 (0.78-1.02)	0.030334633	0.50077331	1.02 (0.97-1.08)	0.97185285	1.00 (0.92-1.09)	0.818732958	0.99 (0.90-1.08)
CD25 on IgD+	27	1.05E-250	0.019918649	1.05 (1.01-1.10)	0.003025438	0.20083635	1.07 (0.97-1.20)	-0.004055961	0.069849907	1.05 (1.00-1.10)	0.324874463	1.04 (0.96-1.13)	0.326123857	1.05 (0.96-1.15)
B cell AC	26	3.23E-138	0.021062263	1.05 (1.01-1.09)	0.167450824	0.54670458	1.03 (0.93-1.15)	0.001825192	0.473194316	1.02 (0.96-1.09)	0.678083744	1.02 (0.93-1.11)	0.799675623	1.02 (0.91-1.14)
Granulocyte %leukocyte	20	3.86E-98	0.047184644	1.05 (1.00-1.10)	0.136333918	0.29499248	1.08 (0.94-1.24)	-0.005226419	0.095656543	1.06 (0.99-1.14)	0.235884406	1.08 (0.96-1.21)	0.263809407	1.07 (0.95-1.21)
CD14 on CD14+ CD16- monocyte	14	2.69E-79	0.04972913	1.05 (1.00-1.11)	0.447114414	0.30951684	1.09 (0.93-1.29)	-0.006669193	0.062348359	1.07 (1.00-1.15)	0.160838742	1.07 (0.98-1.16)	0.493597939	1.04 (0.93-1.18)
CX3CR1 on CD14+ CD16+ monocyte	42	0	0.000384909	1.04 (1.02-1.07)	0.103061316	0.07128894	1.06 (1.00-1.13)	-0.004391232	0.000252554	1.07 (1.03-1.11)	0.000247501	1.07 (1.04-1.11)	0.020907307	1.07 (1.01-1.14)
CD33dim HLA DR+ CD11b- %CD33dim HLA DR+	22	1.19E-246	0.000442126	1.04 (1.02-1.06)	0.147499491	0.39456854	1.02 (0.97-1.07)	0.004939005	0.000834126	1.05 (1.02-1.08)	0.017615959	1.05 (1.01-1.08)	0.308920096	1.03 (0.98-1.08)
CX3CR1 on CD14+ CD16- monocyte	40	0	0.002336406	1.04 (1.01-1.07)	0.032281909	0.13845473	1.05 (0.99-1.12)	-0.002128669	0.009566227	1.05 (1.01-1.08)	0.010593545	1.05 (1.01-1.09)	0.711166299	1.01 (0.95-1.08)
CD14 on CD33dim HLA DR+ CD11b+	15	1.35E-91	0.007763739	1.04 (1.01-1.07)	0.060172559	0.98989516	1.00 (0.89-1.13)	0.011421017	0.006504199	1.06 (1.02-1.11)	0.041149337	1.09 (1.01-1.18)	0.097218579	1.09 (0.99-1.21)
CD20 on IgD+ CD38dim	37	6.60E-287	0.013021581	1.04 (1.01-1.07)	0.081299267	0.8584614	0.99 (0.89-1.11)	0.009182609	0.325462477	1.02 (0.98-1.07)	0.392215666	1.03 (0.96-1.11)	0.518607227	1.03 (0.94-1.13)
CD25 on IgD+ CD24+	31	0	0.033245633	1.04 (1.00-1.08)	0.001334107	0.11616242	1.09 (0.98-1.20)	-0.009689349	0.056806106	1.05 (1.00-1.10)	0.315645005	1.05 (0.96-1.16)	0.28633293	1.05 (0.96-1.16)
CD25 on IgD+ CD24-	36	0	0.036185629	1.04 (1.00-1.07)	0.012805344	0.29866854	1.04 (0.96-1.13)	-0.001193125	0.078472275	1.04 (1.00-1.09)	0.383380338	1.03 (0.96-1.10)	0.753841049	1.01 (0.93-1.10)
CD24+ CD27+ %lymphocyte	27	1.54E-161	0.041610468	1.04 (1.00-1.08)	0.429625169	0.82570571	0.99 (0.90-1.09)	0.008197879	0.145660342	1.04 (0.99-1.10)	0.708212192	1.02 (0.93-1.11)	0.528265712	1.04 (0.93-1.16)
EM DN (CD4-CD8-) %DN	22	1.08E-127	0.042901943	1.04 (1.00-1.08)	0.279677083	0.39135063	1.04 (0.95-1.14)	-0.000518161	0.033140463	1.06 (1.00-1.12)	0.068575705	1.08 (1.00-1.17)	0.210027031	1.07 (0.97-1.18)
CD25hi CD45RA+ CD4 not Treg %CD4+	38	2.34E-267	0.015062055	1.03 (1.01-1.06)	0.405420793	0.17049054	1.03 (0.99-1.08)	-0.001149494	0.089728482	1.03 (1.00-1.07)	0.247956726	1.03 (0.98-1.07)	0.394431178	1.03 (0.96-1.10)
TD CD8br %CD8br	45	0	0.022649335	1.03 (1.00-1.06)	0.869232175	0.95474486	1.00 (0.92-1.08)	0.007272145	0.11893643	1.03 (0.99-1.08)	0.435772358	1.03 (0.96-1.10)	0.584658301	1.02 (0.95-1.10)
CD25 on IgD+ CD38- unsw mem	22	4.80E-189	0.031275736	1.03 (1.00-1.07)	0.283110015	0.67288837	1.02 (0.92-1.13)	0.002731604	0.109959556	1.04 (0.99-1.08)	0.325693184	1.03 (0.97-1.10)	0.356546048	1.04 (0.96-1.11)
Resting Treg AC	30	2.31E-217	0.037184114	1.03 (1.00-1.07)	0.477298039	0.13035178	1.07 (0.98-1.17)	-0.00846657	0.022409005	1.06 (1.01-1.11)	0.059105199	1.07 (1.00-1.14)	0.080220196	1.09 (0.99-1.19)
BAFF-R on IgD+ CD38- unsw mem	115	NA	0.032371149	0.99 (0.98-1.00)	0.068784494	0.02085066	0.98 (0.96-1.00)	0.005606684	0.031214965	0.98 (0.96-1.00)	0.049464245	0.98 (0.96-1.00)	0.078568603	0.96 (0.92-1.00)
BAFF-R on transitional	153	NA	0.036110675	0.99 (0.97-1.00)	0.005502456	0.04555578	0.98 (0.96-1.00)	0.00301988	0.084711994	0.98 (0.96-1.00)	0.018067556	0.97 (0.95-1.00)	0.171583246	1.03 (0.99-1.08)
Activated & secreting Treg %CD4 Treg	71	0	0.016525173	0.98 (0.96-1.00)	0.259210521	0.50186107	0.99 (0.95-1.03)	-0.00319748	0.123829636	0.98 (0.94-1.01)	0.172926856	0.97 (0.94-1.01)	0.070369371	0.95 (0.90-1.00)
CD11b on CD14+ monocyte	27	0	0.018078465	0.98 (0.96-1.00)	0.070012984	0.16077548	0.96 (0.91-1.01)	0.006584049	0.000793934	0.95 (0.92-0.98)	0.002180639	0.95 (0.92-0.98)	0.078422563	0.93 (0.87-1.00)
CD3 on activated & secreting Treg	71	0	0.037554898	0.98 (0.96-1.00)	0.005330562	0.64791035	1.01 (0.96-1.06)	-0.009553343	0.089254449	0.97 (0.95-1.00)	0.263380744	1.03 (0.98-1.09)	0.140816317	0.94 (0.87-1.02)
CD40 on monocytes	58	0	0.037670015	0.98 (0.95-1.00)	0.000912132	0.13206331	1.03 (0.99-1.07)	-0.018108544	0.081362501	0.97 (0.94-1.00)	0.092784469	0.97 (0.94-1.00)	0.167610305	0.96 (0.90-1.02)
CD16 on CD14- CD16+ monocyte	133	NA	4.87E-05	0.97 (0.96-0.99)	0.011812113	0.00014431	0.95 (0.93-0.98)	0.007080929	0.000486456	0.96 (0.94-0.98)	0.00011938	0.96 (0.94-0.98)	0.674338723	1.01 (0.96-1.06)
CD27 on IgD- CD38dim	69	0	0.001633186	0.97 (0.95-0.99)	0.059022292	0.01350339	0.93 (0.89-0.98)	0.008320997	0.149536205	0.98 (0.94-1.01)	0.914515663	1.00 (0.92-1.08)	0.507316194	0.97 (0.90-1.05)
CD64 on CD14+ CD16+ monocyte	60	0	0.014835101	0.97 (0.95-0.99)	0.046854211	0.30406046	0.98 (0.94-1.02)	-0.001223219	0.417373661	0.99 (0.96-1.02)	0.995884477	1.00 (0.94-1.06)	0.670766044	0.99 (0.92-1.05)
CD28 on resting Treg	39	0	0.018610041	0.97 (0.94-0.99)	0.380433416	0.11180194	0.95 (0.90-1.01)	0.004434578	0.484644608	0.99 (0.95-1.03)	0.607417613	0.99 (0.94-1.04)	0.589933525	0.98 (0.91-1.05)
CD3 on secreting Treg	56	0	0.021594237	0.97 (0.94-1.00)	8.91E-05	0.76645301	0.99 (0.93-1.05)	-0.006377902	0.048110486	0.97 (0.93-1.00)	0.701051118	1.01 (0.95-1.08)	0.166931389	0.94 (0.86-1.03)
CD11b on Mo MDSC	18	1.90E-139	0.045394561	0.97 (0.94-1.00)	0.025285651	0.1685151	0.94 (0.87-1.02)	0.008162981	0.001908949	0.94 (0.91-0.98)	0.0036935	0.93 (0.89-0.97)	0.059757639	0.92 (0.86-1.00)
CD33dim HLA DR+ CD11b+ AC	22	5.09E-143	0.047627694	0.97 (0.94-1.00)	0.42466229	0.35017339	0.96 (0.89-1.04)	0.002390588	0.214732374	0.97 (0.93-1.02)	0.953591653	1.00 (0.91-1.09)	0.752466216	0.99 (0.91-1.07)
CD33dim HLA DR+ CD11b+ %CD33dim HLA DR+	24	3.90E-273	4.99E-05	0.96 (0.94-0.98)	0.066845121	0.555986	0.98 (0.93-1.04)	-0.008882064	0.00188511	0.95 (0.93-0.98)	0.009211775	0.95 (0.92-0.99)	0.105909376	0.96 (0.91-1.01)
CD3 on CD39+ secreting Treg	55	0	0.00187471	0.96 (0.94-0.99)	0.009238494	0.11207607	0.95 (0.89-1.01)	0.003166507	0.002528505	0.95 (0.92-0.98)	0.117923721	0.92 (0.84-1.02)	0.074508039	0.92 (0.84-1.01)
HLA DR on plasmacytoid DC	66	0	0.002203982	0.96 (0.93-0.99)	1.79E-10	0.13262685	0.95 (0.90-1.01)	0.001637944	0.020169835	0.97 (0.94-0.99)	0.094068773	0.96 (0.91-1.01)	0.414856845	0.97 (0.91-1.04)
CD3 on HLA DR+ T cell	36	2.43E-246	0.005002755	0.96 (0.93-0.99)	0.051614942	0.13367844	0.94 (0.86-1.02)	0.005687976	0.010491574	0.94 (0.90-0.99)	0.15015894	0.95 (0.89-1.02)	0.203314695	0.95 (0.87-1.03)
HVEM on EM CD4+	15	6.60E-87	0.005229892	0.96 (0.93-0.99)	0.214675863	0.07764742	0.92 (0.84-1.00)	0.014180132	0.092265955	0.96 (0.92-1.01)	0.323396842	0.96 (0.89-1.04)	0.532385624	0.97 (0.90-1.06)
HVEM on CD4+	16	3.97E-103	0.006265563	0.96 (0.93-0.99)	0.152177881	0.57383185	0.97 (0.86-1.08)	-0.00190622	0.02965154	0.95 (0.92-1.00)	0.167301254	0.95 (0.89-1.02)	0.270597121	0.96 (0.89-1.03)
CD24 on memory B cell	28	6.68E-229	0.01010865	0.96 (0.93-0.99)	0.682235705	0.13716164	0.95 (0.89-1.01)	0.00156499	0.40475693	0.98 (0.94-1.03)	0.507722706	0.98 (0.92-1.04)	0.736378722	0.99 (0.91-1.07)
CD3 on EM CD8br	32	1.18E-227	0.019474594	0.96 (0.94-0.99)	0.115348447	0.48113291	0.97 (0.90-1.05)	-0.001546053	0.3701742	0.98 (0.93-1.03)	0.753198529	0.99 (0.92-1.06)	0.614382004	0.98 (0.90-1.06)
CD19 on IgD+ CD38- unsw mem	17	1.86E-106	0.022748242	0.96 (0.93-0.99)	0.440492404	0.15494045	0.95 (0.89-1.02)	0.005068064	0.724845306	0.99 (0.94-1.04)	0.691181214	0.99 (0.94-1.04)	0.156549955	0.95 (0.88-1.02)
CD66b on CD66b++ myeloid cell	18	6.66E-102	0.023047838	0.96 (0.93-0.99)	0.074180956	0.77299855	0.98 (0.86-1.12)	-0.005900973	0.785861482	0.99 (0.94-1.05)	0.760785721	0.99 (0.93-1.05)	0.234346452	0.95 (0.88-1.03)
CD11c on monocyte	21	5.73E-158	0.028557533	0.96 (0.93-1.00)	0.368467771	0.03785708	0.91 (0.83-0.99)	0.013898761	0.090925441	0.96 (0.91-1.01)	0.215871349	0.96 (0.91-1.02)	0.514738985	0.98 (0.91-1.05)
CD8 on CM CD8br	23	3.29E-186	0.046350478	0.96 (0.92-1.00)	0.036167842	0.10985958	0.92 (0.84-1.01)	0.010480333	0.010875237	0.94 (0.90-0.99)	0.020471964	0.94 (0.89-0.99)	0.421311754	0.97 (0.90-1.04)
CD16 on CD14+ CD16+ monocyte	64	0	3.49E-09	0.95 (0.93-0.96)	0.101582404	9.95E-05	0.92 (0.88-0.95)	0.009582066	3.25E-06	0.94 (0.91-0.96)	0.000230024	0.94 (0.91-0.97)	0.722828613	0.99 (0.94-1.05)
HVEM on CM CD8br	14	2.44E-91	0.000215738	0.95 (0.92-0.97)	0.105363251	0.09313521	0.90 (0.80-1.01)	0.016676149	0.015894895	0.95 (0.91-0.99)	0.157264491	0.93 (0.84-1.02)	0.133780871	0.92 (0.84-1.02)
PDL-1 on CD14- CD16+ monocyte	42	0	0.00056732	0.95 (0.92-0.98)	0.012333225	0.29569164	0.96 (0.90-1.03)	-0.004093802	0.001201001	0.94 (0.90-0.97)	0.050876598	0.93 (0.86-1.00)	0.660596542	1.02 (0.94-1.11)
HVEM on CD45RA- CD4+	16	6.32E-106	0.001205325	0.95 (0.93-0.98)	0.542668084	0.37999774	0.97 (0.90-1.04)	-0.003643665	0.064927834	0.96 (0.93-1.00)	0.350871182	0.97 (0.91-1.03)	0.289129307	0.97 (0.91-1.03)

CD28 on CD39+ CD8br	15	9.50E-82	0.009887319	0.95 (0.91-0.99)	0.244519368	0.78450786	0.99 (0.89-1.09)	-0.009213498	0.766144899	0.99 (0.93-1.05)	0.821165908	0.99 (0.92-1.07)	0.83343289	0.99 (0.90-1.09)
CD4 on CD45RA+ CD4+	20	5.44E-119	0.010644389	0.95 (0.92-0.99)	0.152707456	0.74311589	0.98 (0.89-1.09)	-0.006769048	0.054173849	0.95 (0.91-1.00)	0.136913689	0.95 (0.90-1.01)	0.434642338	0.97 (0.91-1.04)
CD8 on NKT	16	2.96E-77	0.01850318	0.95 (0.90-0.99)	0.221322463	0.45631174	0.94 (0.81-1.10)	0.000525545	0.375473253	0.97 (0.90-1.04)	0.909437177	0.99 (0.90-1.10)	0.028597262	0.86 (0.76-0.97)
CD19 on IgD+ CD38dim	22	3.34E-125	0.024849702	0.95 (0.91-0.99)	0.566063923	0.0333282	0.89 (0.80-0.98)	0.014084649	0.13969368	0.96 (0.90-1.01)	0.613961051	0.97 (0.87-1.08)	0.246278052	0.93 (0.82-1.05)
CD14+ CD16- monocyte AC	19	8.39E-104	0.026378733	0.95 (0.90-0.99)	0.342718654	0.33536913	0.92 (0.77-1.09)	0.00525411	0.025212968	0.92 (0.86-0.99)	0.246847159	0.94 (0.86-1.04)	0.344375583	0.95 (0.85-1.06)
CD4 on activated Treg	25	2.81E-147	0.041185735	0.95 (0.91-1.00)	0.049047034	0.28550548	1.06 (0.95-1.19)	-0.024123717	0.33569916	0.97 (0.92-1.03)	0.257845396	0.96 (0.88-1.03)	0.427321004	0.96 (0.86-1.06)
HLA DR+ NK %CD3- lymphocyte	53	0	4.71E-10	0.94 (0.92-0.96)	0.087542378	0.00116175	0.92 (0.87-0.96)	0.005051063	6.83E-06	0.93 (0.90-0.96)	0.000955168	0.93 (0.89-0.97)	0.05496085	0.93 (0.87-1.00)
CD14+ CD16+ monocyte %monocyte	58	0	9.93E-10	0.94 (0.92-0.96)	0.1834176	0.03023106	0.95 (0.90-0.99)	-0.001928806	5.90E-06	0.93 (0.90-0.96)	4.21E-05	0.92 (0.89-0.96)	0.02125497	0.93 (0.88-0.99)
HLA DR+ NK %NK	55	0	3.54E-09	0.94 (0.92-0.96)	0.065018974	0.00116425	0.91 (0.86-0.96)	0.006715601	1.41E-05	0.93 (0.90-0.96)	0.000438366	0.93 (0.90-0.97)	0.220933848	0.96 (0.89-1.03)
CCR2 on myeloid DC	22	1.50E-219	3.52E-05	0.94 (0.91-0.97)	0.230544526	0.03855951	0.90 (0.83-0.99)	0.010309164	0.002245009	0.93 (0.89-0.98)	0.009537535	0.93 (0.88-0.98)	0.047752752	0.93 (0.86-0.99)
BAFF-R on IgD- CD38br	31	9.62E-250	8.89E-05	0.94 (0.92-0.97)	0.060221178	0.17010197	0.95 (0.89-1.02)	-0.001994065	0.00545114	0.94 (0.89-0.98)	0.081837248	0.94 (0.88-1.01)	0.153311033	0.93 (0.85-1.02)
CD4 on CM CD4 +	18	1.37E-108	0.000589405	0.94 (0.90-0.97)	0.237513945	0.81730905	0.98 (0.87-1.12)	-0.010308439	0.038117564	0.95 (0.90-1.00)	0.103638033	0.95 (0.90-1.01)	0.243702404	0.96 (0.89-1.03)
FSC-A on myeloid DC	23	3.57E-180	0.00230404	0.94 (0.91-0.98)	0.01461629	0.73634965	0.98 (0.90-1.08)	-0.010743246	0.401781914	0.98 (0.93-1.03)	0.889640966	1.00 (0.93-1.07)	0.7644881	0.99 (0.89-1.09)
Plasmacytoid DC %DC	33	0	0.002712317	0.94 (0.91-0.98)	0.000138197	0.0546322	0.91 (0.82-1.00)	0.008704417	0.000319094	0.93 (0.89-0.97)	0.001989329	0.92 (0.87-0.96)	0.063317119	0.93 (0.86-1.00)
CD25 on CD45RA- CD4 not Treg	18	6.56E-114	0.003804242	0.94 (0.90-0.98)	0.087400638	0.79597923	0.99 (0.89-1.09)	-0.012795375	0.081077129	0.94 (0.88-1.01)	0.289522843	0.96 (0.89-1.03)	0.753704066	0.98 (0.88-1.09)
CD3 on CD39+ resting Treg	19	3.62E-112	0.006247401	0.94 (0.90-0.98)	0.528554482	0.44251286	1.05 (0.93-1.18)	-0.022754614	0.178080407	0.96 (0.90-1.02)	0.335621616	0.96 (0.90-1.04)	0.505676629	0.97 (0.89-1.06)
CCR2 on CD14- CD16-	13	4.25E-72	0.023746177	0.94 (0.90-0.99)	0.223182732	0.09983378	0.88 (0.77-1.01)	0.013951136	0.386768248	0.97 (0.90-1.04)	0.531396627	0.97 (0.88-1.06)	0.451951105	0.96 (0.85-1.07)
HLA DR+ NK AC	56	0	6.31E-07	0.93 (0.91-0.96)	0.009859602	0.00205171	0.91 (0.86-0.96)	0.006995763	8.74E-06	0.92 (0.89-0.96)	0.000248714	0.92 (0.89-0.96)	0.032564078	0.92 (0.86-0.99)
FSC-A on HLA DR+ NK	34	0	8.82E-05	0.93 (0.90-0.97)	0.000345842	0.25859641	0.96 (0.89-1.03)	-0.006686476	0.000119669	0.93 (0.89-0.96)	0.002373087	0.91 (0.86-0.96)	0.514297546	0.97 (0.89-1.06)
IgD+ CD38- AC	14	2.75E-66	0.001096199	0.93 (0.89-0.97)	0.787433935	0.15188297	0.92 (0.83-1.02)	0.001431612	0.002922712	0.91 (0.86-0.97)	0.065873729	0.89 (0.79-1.00)	0.047980487	0.89 (0.80-0.99)
NK AC	16	4.81E-100	0.006014855	0.93 (0.88-0.98)	0.05103095	0.44027838	0.93 (0.77-1.12)	0.000643548	0.173422486	0.95 (0.88-1.02)	0.402884331	0.97 (0.90-1.04)	0.710084989	0.98 (0.89-1.08)
CD3- lymphocyte %leukocyte	14	3.19E-73	0.006803781	0.93 (0.89-0.98)	0.809826124	0.41578257	0.95 (0.83-1.08)	-0.002224495	0.030988855	0.93 (0.87-0.99)	0.143737718	0.93 (0.84-1.02)	0.176033812	0.93 (0.84-1.03)
CD19 on unsw mem	23	3.21E-127	0.039335946	0.93 (0.87-1.00)	0.002132629	0.01431505	0.83 (0.73-0.95)	0.021335002	0.019035576	0.92 (0.86-0.99)	0.13270118	0.91 (0.81-1.02)	0.23030672	0.92 (0.81-1.05)
CD14+ CD16+ monocyte AC	52	0	6.82E-12	0.92 (0.90-0.94)	0.567597778	0.00152767	0.92 (0.87-0.96)	0.002056962	9.11E-07	0.92 (0.89-0.95)	4.55E-05	0.92 (0.88-0.95)	0.022253057	0.93 (0.87-0.99)
HLA DR on DC	64	0	4.14E-10	0.92 (0.90-0.95)	3.18E-10	0.01184167	0.93 (0.88-0.98)	-0.00337184	1.91E-06	0.93 (0.90-0.96)	0.000134019	0.93 (0.89-0.96)	0.002898984	0.92 (0.87-0.97)
SSC-A on HLA DR+ NK	40	0	7.67E-10	0.92 (0.90-0.95)	0.1317143	0.01635259	0.93 (0.87-0.98)	-0.000618419	9.01E-08	0.90 (0.87-0.94)	0.000140539	0.90 (0.85-0.94)	0.009040753	0.90 (0.83-0.97)
HLA DR on myeloid DC	50	0	9.93E-10	0.92 (0.89-0.94)	7.64E-05	0.000126	0.86 (0.79-0.92)	0.019595628	4.88E-10	0.90 (0.87-0.93)	4.84E-07	0.89 (0.86-0.93)	0.001007306	0.90 (0.85-0.96)
CD16+ monocyte %monocyte	33	0	5.99E-07	0.92 (0.90-0.95)	0.117520614	0.00026494	0.85 (0.78-0.92)	0.018527585	0.000991827	0.92 (0.87-0.97)	0.003249909	0.90 (0.84-0.96)	0.020691075	0.89 (0.82-0.98)
CD14- CD16+ monocyte AC	25	3.54E-213	8.55E-06	0.92 (0.89-0.96)	0.241780559	0.01200531	0.87 (0.79-0.96)	0.012484543	0.005298352	0.93 (0.88-0.98)	0.061662507	0.93 (0.87-1.00)	0.189142027	0.94 (0.86-1.03)
CD4 on EM CD4+	16	3.55E-99	5.00E-05	0.92 (0.88-0.96)	0.059883283	0.07258608	0.87 (0.75-1.00)	0.01171775	0.035016138	0.94 (0.88-1.00)	0.068689846	0.94 (0.89-1.00)	0.198711098	0.94 (0.87-1.03)
CD14- CD16+ monocyte %monocyte	28	3.34E-223	0.000361238	0.92 (0.89-0.97)	0.029055449	0.0269919	0.89 (0.80-0.98)	0.00788842	0.001882332	0.91 (0.86-0.97)	0.025186035	0.90 (0.82-0.98)	0.066489716	0.88 (0.78-1.00)
Naive DN (CD4-CD8-) %T cell	15	1.40E-73	0.001615846	0.92 (0.87-0.97)	0.369844375	0.92904036	0.99 (0.81-1.22)	-0.012412447	0.016520073	0.90 (0.83-0.98)	0.042017637	0.86 (0.76-0.98)	0.034075261	0.84 (0.72-0.97)
BAFF-R on CD20-	17	4.90E-112	0.004817677	0.92 (0.87-0.98)	0.04512648	0.45399644	0.95 (0.84-1.08)	-0.007341328	0.011319901	0.91 (0.85-0.98)	0.057555716	0.91 (0.84-1.00)	0.285662427	0.94 (0.84-1.05)
FSC-A on CD4+	16	1.35E-84	0.028581918	0.92 (0.85-0.99)	0.044324382	0.05243807	0.79 (0.63-0.98)	0.025998015	0.533651873	0.97 (0.89-1.06)	0.912994532	0.99 (0.87-1.14)	0.642968519	0.97 (0.85-1.11)
CD4 on CD39+ activated Treg	23	1.28E-141	9.69E-05	0.91 (0.87-0.95)	0.019403871	0.46923727	1.06 (0.91-1.23)	-0.033975697	0.004717438	0.91 (0.86-0.97)	0.077250527	0.91 (0.82-1.01)	0.075377476	0.90 (0.81-1.01)
CD4 on HLA DR+ CD4+	19	3.19E-105	0.001104904	0.90 (0.85-0.96)	0.001601514	0.93913652	0.99 (0.87-1.14)	-0.020503709	0.017738573	0.92 (0.87-0.99)	0.045557376	0.90 (0.82-0.99)	0.051334971	0.89 (0.79-0.99)
HLA DR++ monocyte %leukocyte	5	1.80E-23	2.73E-06	0.82 (0.75-0.89)	0.553384682	0.28164692	0.70 (0.41-1.19)	0.023945178	0.000242923	0.81 (0.72-0.90)	0.037680351	0.80 (0.69-0.92)	0.041273039	0.80 (0.69-0.93)
**Inflammatory cytokines**														
Interferon gamma levels	18	3.73E-83	0.003908355	1.13 (1.04-1.23)	0.133844326	0.00987501	1.28 (1.09-1.51)	-0.016192382	0.049915573	1.12 (1.00-1.26)	0.169005448	1.12 (0.96-1.32)	0.260264905	1.12 (0.93-1.34)
CD40L receptor levels	46	0	2.44E-06	1.12 (1.07-1.18)	0.015466542	2.52E-05	1.23 (1.13-1.34)	-0.01321663	3.22E-06	1.17 (1.10-1.25)	4.70E-05	1.19 (1.10-1.29)	0.09015907	1.13 (0.98-1.29)
C-X-C motif chemokine 10 levels	31	1.74E-185	0.012361037	1.09 (1.02-1.16)	0.589210371	0.11714146	1.11 (0.98-1.25)	-0.001801337	0.164300704	1.08 (0.97-1.19)	0.566331132	1.04 (0.91-1.19)	0.747348538	0.97 (0.81-1.17)
Tumor necrosis factor levels	25	7.48E-113	0.021107203	1.09 (1.01-1.18)	0.617003352	0.69906803	1.04 (0.87-1.24)	0.005488322	0.1110974	1.09 (0.98-1.22)	0.119066064	1.20 (0.96-1.50)	0.200016878	1.17 (0.93-1.49)
Interleukin-17A levels	19	3.04E-82	0.044023253	1.09 (1.00-1.18)	0.327353596	0.87404126	0.99 (0.82-1.18)	0.011290297	0.36918181	1.06 (0.94-1.19)	0.887609051	0.99 (0.81-1.20)	0.56575239	0.94 (0.76-1.16)
Fms-related tyrosine kinase 3 ligand levels	54	0	0.006263796	1.08 (1.02-1.15)	0.004226918	0.01088156	1.15 (1.04-1.27)	-0.00696062	0.000136844	1.16 (1.07-1.24)	0.004494716	1.15 (1.05-1.26)	0.262026253	1.12 (0.92-1.36)
Fibroblast growth factor 21 levels	34	5.69E-227	0.006317857	1.08 (1.02-1.15)	0.79919423	0.10535789	1.12 (0.98-1.28)	-0.00347251	0.013660637	1.11 (1.02-1.21)	0.068703459	1.12 (1.00-1.27)	0.448967733	1.06 (0.91-1.25)
Natural killer cell receptor 2B4 levels	41	0	0.004152972	1.07 (1.02-1.13)	0.063901655	0.12322984	1.09 (0.98-1.22)	-0.001994869	0.18414989	1.06 (0.97-1.16)	0.568435682	1.03 (0.94-1.13)	0.963406781	1.00 (0.86-1.16)
Monocyte chemoattractant protein-4 levels	46	0	0.006100844	1.07 (1.02-1.13)	0.038450314	0.86454949	0.99 (0.89-1.10)	0.010487809	0.745166578	1.01 (0.95-1.08)	0.903919279	1.00 (0.93-1.08)	0.046028845	1.17 (1.01-1.36)
T-cell surface glycoprotein CD6 isoform levels	60	0	5.06E-05	1.06 (1.03-1.09)	0.200106669	0.53456536	1.02 (0.96-1.07)	0.009495158	0.02025248	1.05 (1.01-1.10)	0.004902918	1.07 (1.02-1.11)	0.010992996	1.11 (1.03-1.20)
Interleukin-10 receptor subunit beta levels	54	0	0.000786742	1.06 (1.02-1.09)	0.201278248	0.06164985	1.06 (1.00-1.13)	-0.000754186	0.013556169	1.07 (1.01-1.12)	0.007472993	1.07 (1.02-1.12)	0.185986999	1.08 (0.97-1.20)
Interleukin-18 levels	46	0	0.006734549	1.06 (1.02-1.11)	0.861163077	0.52102807	1.03 (0.93-1.15)	0.003504551	0.086263247	1.06 (0.99-1.13)	0.118843828	1.08 (0.98-1.18)	0.040369755	1.15 (1.01-1.32)
Signaling lymphocytic activation molecule levels	43	3.18E-260	0.037778866	1.06 (1.00-1.11)	0.316896737	0.53610738	1.04 (0.92-1.17)	0.002018012	0.504098928	1.03 (0.94-1.12)	0.560291334	1.04 (0.92-1.16)	0.234106102	1.10 (0.94-1.30)
Interleukin-10 levels	33	1.35E-165	0.042559937	1.06 (1.00-1.13)	0.746128728	0.02107218	1.15 (1.03-1.29)	-0.009370381	0.081993478	1.08 (0.99-1.18)	0.073192064	1.13 (0.99-1.28)	0.089247961	1.17 (0.98-1.39)
C-C motif chemokine 25 levels	108	0	0.001776546	0.97 (0.95-0.99)	0.456005348	0.45524957	0.99 (0.96-1.02)	-0.00399517	0.065405041	0.97 (0.94-1.00)	0.061266084	0.97 (0.95-1.00)	0.422058069	0.98 (0.92-1.03)
C-C motif chemokine 23 levels	44	0	0.000854939	0.95 (0.92-0.98)	0.304004386	0.05656858	0.96 (0.92-1.00)	-0.001454251	0.008562404	0.95 (0.91-0.99)	0.01453612	0.96 (0.93-0.99)	0.231044153	0.96 (0.89-1.03)
Leukemia inhibitory factor receptor levels	40	0	0.001734164	0.93 (0.89-0.97)	0.352106174	0.30296362	0.95 (0.86-1.05)	-0.002349509	0.024039809	0.93 (0.87-0.99)	0.546084105	0.97 (0.89-1.06)	0.154990116	0.90 (0.77-1.04)
Fibroblast growth factor 19 levels	34	4.90E-199	0.004992198	0.92 (0.87-0.97)	0.181005064	0.12348255	0.87 (0.74-1.03)	0.006132918	0.037690682	0.91 (0.84-0.99)	0.075595845	0.89 (0.79-1.01)	0.638106875	0.96 (0.81-1.14)
Interleukin-6 levels	15	4.76E-99	0.02047896	0.90 (0.83-0.98)	0.183748385	0.01777196	0.78 (0.65-0.93)	0.019748656	0.442759836	0.95 (0.83-1.09)	0.006636694	0.78 (0.67-0.91)	0.990977473	1.00 (0.75-1.32)
**Blood metabolites**														
X-12714 levels	20	2.40E-132	0.000291944	1.15 (1.07-1.24)	0.002967592	0.2739867	1.12 (0.92-1.37)	0.005117852	6.58E-05	1.18 (1.09-1.29)	0.00115886	1.21 (1.10-1.34)	0.072486437	1.18 (0.99-1.41)
X-25828 levels	14	5.95E-61	0.015676415	1.14 (1.03-1.27)	0.835086567	0.0941713	1.29 (0.98-1.71)	-0.014815971	0.231503314	1.10 (0.94-1.28)	0.4154187	1.09 (0.89-1.33)	0.387936933	1.11 (0.88-1.39)
3-hydroxylaurate levels	9	2.24E-42	0.02489294	1.13 (1.02-1.25)	0.675133564	0.24571858	1.15 (0.93-1.43)	-0.004009763	0.251208437	1.09 (0.94-1.26)	0.37430207	1.08 (0.92-1.26)	0.58681403	1.06 (0.87-1.29)
1-(1-enyl-palmitoyl)-2-arachidonoyl-GPE (p-16:0/20:4) levels	23	1.01E-120	0.00016604	1.12 (1.06-1.19)	0.186450496	0.52246984	1.04 (0.92-1.19)	0.010447568	0.240057273	1.06 (0.96-1.15)	0.368032266	1.05 (0.95-1.16)	0.488831253	1.05 (0.91-1.21)
X-17335 levels	12	4.00E-55	0.012610903	1.12 (1.03-1.23)	0.97977901	0.18152361	1.14 (0.95-1.36)	-0.002120538	0.151202114	1.09 (0.97-1.24)	0.455201426	1.08 (0.89-1.31)	0.468158437	1.08 (0.89-1.30)
enosine 5'-monophosphate (AMP) to flavin adenine dinucleotide (FAD) r	12	7.45E-71	0.00508131	1.11 (1.03-1.20)	0.793549152	0.29897371	1.11 (0.92-1.35)	-0.000336842	0.04785167	1.11 (1.00-1.22)	0.73265076	1.03 (0.88-1.20)	0.069165637	1.19 (1.00-1.42)
3-methyl-2-oxovalerate to 3-methyl-2-oxobutyrate ratio	21	1.17E-94	0.008804203	1.11 (1.03-1.21)	0.763655516	0.70893184	1.04 (0.86-1.26)	0.008406024	0.05977261	1.11 (1.00-1.24)	0.164818763	1.13 (0.96-1.33)	0.330182881	1.09 (0.92-1.30)
Adenosine 5'-monophosphate (AMP) to serine ratio	13	6.36E-62	0.010160494	1.11 (1.03-1.20)	0.53185895	0.17363305	1.14 (0.96-1.35)	-0.003213719	0.149275068	1.08 (0.97-1.21)	0.286407632	1.08 (0.94-1.25)	0.249581447	1.10 (0.94-1.29)
3-hydroxyoctanoylcarnitine (2) levels	13	3.73E-61	0.014114049	1.11 (1.02-1.20)	0.95292522	0.44896404	1.07 (0.91-1.25)	0.004649558	0.059946168	1.11 (1.00-1.24)	0.138316508	1.15 (0.97-1.35)	0.663703245	1.04 (0.88-1.24)
Picolinoylglycine levels	24	1.73E-111	0.004698156	1.10 (1.03-1.17)	0.092039463	0.47576993	1.07 (0.89-1.27)	0.00354177	0.321539788	1.05 (0.95-1.16)	0.557955458	1.04 (0.92-1.17)	0.455115354	1.06 (0.92-1.22)
Adenosine 5'-diphosphate (ADP) to glycerate ratio	13	3.33E-61	0.007886283	1.10 (1.03-1.18)	0.420549603	0.62297088	1.06 (0.85-1.32)	0.005264605	0.263472691	1.06 (0.96-1.18)	0.063469535	1.21 (1.01-1.46)	0.110630451	1.20 (0.97-1.49)
Arginine to citrulline ratio	36	1.89E-192	0.007886288	1.10 (1.02-1.17)	0.030117717	0.04828388	1.20 (1.01-1.44)	-0.01049336	0.00223491	1.14 (1.05-1.24)	0.085149892	1.12 (0.99-1.27)	0.382954783	1.08 (0.91-1.29)
X-21442 levels	16	1.04E-78	0.008181042	1.10 (1.03-1.18)	0.145437597	0.34964241	1.14 (0.88-1.48)	-0.004236903	0.280549169	1.06 (0.96-1.17)	0.546682003	1.06 (0.89-1.25)	0.57564277	1.06 (0.87-1.30)
Leucine to N-palmitoyl-sphingosine (d18:1 to 16:0) ratio	20	4.12E-90	0.021874264	1.10 (1.01-1.19)	0.230156275	0.21358528	1.20 (0.91-1.59)	-0.008442184	0.234355534	1.07 (0.96-1.20)	0.7657931	1.03 (0.86-1.23)	0.717740413	1.04 (0.85-1.27)
Glucose to N-palmitoyl-sphingosine (d18:1 to 16:0) ratio	22	1.64E-99	0.013520206	1.09 (1.02-1.17)	0.561256195	0.38139713	1.07 (0.92-1.25)	0.002354126	0.105700658	1.09 (0.98-1.20)	0.024291489	1.23 (1.04-1.44)	0.072325451	1.21 (0.99-1.48)
Palmitoleoylcarnitine (C16:1) levels	16	4.91E-82	0.037072185	1.09 (1.01-1.18)	0.151812831	0.41800047	1.11 (0.86-1.43)	-0.002720623	0.075124642	1.12 (0.99-1.26)	0.575318899	1.08 (0.84-1.38)	0.422998865	1.10 (0.87-1.40)
Glycolithocholate to glycolithocholate sulfate ratio	30	5.23E-184	0.001181528	1.08 (1.03-1.13)	0.400072572	0.23690402	1.08 (0.96-1.21)	0.000530638	0.02638072	1.08 (1.01-1.16)	0.251644064	1.06 (0.96-1.16)	0.622693444	1.03 (0.91-1.17)
1-palmitoyl-2-linoleoyl-GPI (16:0/18:2) levels	22	2.77E-136	0.004819346	1.08 (1.02-1.15)	0.631292575	0.03729674	1.13 (1.02-1.27)	-0.006681942	0.256083553	1.05 (0.97-1.14)	0.364542621	1.05 (0.95-1.16)	0.68528228	1.03 (0.89-1.20)
1-(1-enyl-stearoyl)-2-oleoyl-GPE (p-18:0/18:1) levels	19	1.50E-95	0.012024435	1.08 (1.02-1.16)	0.944866285	0.13824314	1.09 (0.98-1.21)	-0.000701084	0.104973994	1.07 (0.99-1.17)	0.154849149	1.08 (0.97-1.20)	0.161354265	1.10 (0.97-1.25)
Stearoylcarnitine levels	23	1.35E-138	0.013010495	1.08 (1.02-1.14)	0.339629163	0.73938682	1.03 (0.87-1.22)	0.005470647	0.002557361	1.15 (1.05-1.25)	0.039943646	1.15 (1.01-1.30)	0.139183328	1.13 (0.97-1.33)
Succinate to acetoacetate ratio	13	6.44E-60	0.022075903	1.08 (1.01-1.16)	0.069989531	0.57865274	1.05 (0.90-1.22)	0.007260668	0.154074402	1.08 (0.97-1.19)	0.616244923	1.04 (0.91-1.18)	0.085820396	1.19 (0.99-1.42)
5-hydroxymethyl-2-furoylcarnitine levels	21	3.84E-97	0.022353265	1.08 (1.01-1.16)	0.031628645	0.94027779	0.99 (0.85-1.16)	0.012853792	0.006112539	1.11 (1.03-1.20)	0.052641731	1.13 (1.01-1.26)	0.100321821	1.12 (0.98-1.28)
X-24334 levels	17	3.67E-99	0.031034522	1.08 (1.01-1.15)	0.787489884	0.44307071	1.07 (0.90-1.27)	0.00077982	0.142318521	1.07 (0.98-1.18)	0.260454447	1.08 (0.95-1.23)	0.252887352	1.10 (0.94-1.29)
Formiminoglutamate levels	24	1.35E-111	0.032095542	1.08 (1.01-1.15)	0.528219614	0.65216629	1.04 (0.89-1.21)	0.004164048	0.14647109	1.07 (0.98-1.18)	0.870121349	0.99 (0.83-1.17)	0.551757422	1.06 (0.88-1.27)
1-(1-enyl-stearoyl)-2-arachidonoyl-GPE (p-18:0/20:4) levels	32	1.35E-245	0.003135217	1.07 (1.02-1.11)	0.172470426	0.04327887	1.12 (1.01-1.23)	-0.00768967	0.002015258	1.11 (1.04-1.19)	0.01478296	1.11 (1.02-1.19)	0.187652085	1.11 (0.95-1.29)
Adenosine 5'-diphosphate (ADP) to mannose ratio	18	3.80E-86	0.010997628	1.07 (1.01-1.12)	0.743911997	0.20838287	1.08 (0.96-1.21)	-0.00186381	0.02797063	1.08 (1.01-1.16)	0.538008456	1.03 (0.94-1.14)	0.960240725	1.00 (0.87-1.14)
Thioproline levels	23	1.99E-109	0.024811139	1.07 (1.01-1.14)	0.079655259	0.87252559	0.99 (0.88-1.12)	0.012000448	0.366213895	1.04 (0.95-1.15)	0.620987765	1.03 (0.93-1.14)	0.309957425	1.10 (0.92-1.33)
Ursodeoxycholate levels	18	7.83E-88	0.033128452	1.07 (1.01-1.15)	0.556096197	0.70874287	1.04 (0.86-1.24)	0.00504022	0.089395983	1.08 (0.99-1.19)	0.288280765	1.08 (0.94-1.24)	0.159041946	1.12 (0.96-1.29)
Gamma-glutamylglutamate levels	30	9.93E-145	0.045347915	1.07 (1.00-1.14)	0.085491496	0.78253308	0.97 (0.80-1.18)	0.010755387	0.078751956	1.09 (0.99-1.20)	0.023040475	1.24 (1.04-1.48)	0.058883797	1.25 (1.00-1.56)

Cortisol levels (plasma)	19	5.73E-91	0.048862214	1.07 (1.00-1.15)	0.469961241	0.26170659	1.10 (0.93-1.30)	-0.003643248	0.360727583	1.05 (0.95-1.15)	0.48235838	1.05 (0.92-1.20)	0.499167126	1.05 (0.91-1.23)
X-24544 levels	60	0	4.18E-05	1.06 (1.03-1.09)	0.375263362	0.06384636	1.05 (1.00-1.10)	0.00218375	0.026573508	1.06 (1.01-1.11)	0.030006343	1.05 (1.01-1.10)	0.164504412	1.07 (0.98-1.16)
Ximenoylcarnitine (C26:1) levels	37	0	0.00223173	1.06 (1.02-1.11)	0.484049969	0.00912126	1.12 (1.03-1.22)	-0.009161796	0.236456762	1.04 (0.98-1.11)	0.374202852	1.04 (0.96-1.12)	0.814333107	1.01 (0.90-1.14)
Adenosine 5'-diphosphate (ADP) to flavin adenine dinucleotide (FAD) rati	22	6.06E-113	0.004248732	1.06 (1.02-1.11)	0.393030688	0.27219288	1.05 (0.97-1.14)	0.003350826	0.171397255	1.05 (0.98-1.12)	0.336732767	1.04 (0.96-1.13)	0.428807896	1.05 (0.94-1.17)
Nervonoylcarnitine (C24:1) levels	30	2.37E-220	0.007411309	1.06 (1.02-1.11)	0.976441529	0.09002561	1.10 (0.99-1.23)	-0.00465501	0.025460614	1.08 (1.01-1.15)	0.084988919	1.07 (0.99-1.16)	0.39674883	1.05 (0.94-1.17)
Adenosine 5'-monophosphate (AMP) to glycine ratio	24	2.49E-187	0.016634192	1.06 (1.01-1.12)	0.406146108	0.24706147	1.07 (0.96-1.19)	-0.000702241	0.355377958	1.04 (0.96-1.12)	0.231330262	1.05 (0.97-1.14)	0.255345732	1.08 (0.95-1.24)
Palmitoyl dihydrosphingomyelin (d18:0/16:0) levels	35	4.02E-190	0.017238762	1.06 (1.01-1.12)	0.319619322	0.41826665	1.06 (0.92-1.21)	0.000683949	0.220734834	1.05 (0.97-1.13)	0.260617974	1.07 (0.95-1.20)	0.281740538	1.08 (0.94-1.25)
Alpha-ketoglutaramate levels	25	1.17E-145	0.023561093	1.06 (1.01-1.12)	0.487973229	0.03437648	1.12 (1.01-1.24)	-0.007981142	0.343139255	1.04 (0.96-1.13)	0.347652644	1.06 (0.94-1.18)	0.41816969	1.07 (0.91-1.26)
Hydantoin-5-propionate levels	19	3.43E-91	0.041994434	1.06 (1.00-1.13)	0.218028915	0.72716036	1.02 (0.90-1.17)	0.005778839	0.438404048	1.04 (0.94-1.15)	0.480031418	1.04 (0.94-1.15)	0.143225397	1.14 (0.96-1.35)
N-acetylputrescine levels	40	0	0.001930071	1.05 (1.02-1.08)	0.286559559	0.22651518	1.03 (0.98-1.09)	0.003218825	0.17837797	1.03 (0.99-1.08)	0.281828981	1.02 (0.98-1.06)	0.960887297	1.00 (0.92-1.09)
Malate levels	18	1.71E-94	0.004077921	1.05 (1.02-1.09)	0.167001153	0.08634505	1.05 (1.00-1.10)	0.001624825	0.044874959	1.05 (1.00-1.10)	0.052472579	1.05 (1.00-1.10)	0.176376685	1.09 (0.97-1.24)
Succinimide levels	19	3.77E-93	0.021052719	1.05 (1.01-1.09)	0.821517954	0.33245665	1.03 (0.97-1.09)	0.004682732	0.188451248	1.04 (0.98-1.10)	0.191758984	1.04 (0.98-1.10)	0.555062293	1.03 (0.94-1.12)
X-16087 levels	29	2.66E-228	0.031163083	1.05 (1.00-1.10)	0.266517565	0.46376987	0.96 (0.87-1.06)	0.013631802	0.140835742	1.06 (0.98-1.14)	0.231604132	1.05 (0.97-1.14)	0.362795527	1.06 (0.93-1.21)
X-17653 levels	28	6.54E-229	0.031176895	1.05 (1.00-1.10)	0.212267352	0.59218372	1.03 (0.93-1.13)	0.004003503	0.973216781	1.00 (0.94-1.07)	0.653815448	0.98 (0.90-1.07)	0.820357703	0.99 (0.88-1.11)
Carnitine to ergothioneine ratio	27	4.40E-190	0.049049012	1.05 (1.00-1.11)	0.137068845	0.02800474	1.21 (1.03-1.42)	-0.017164148	0.029823059	1.09 (1.01-1.19)	0.044808598	1.11 (1.01-1.23)	0.092162924	1.12 (0.99-1.28)
Isoleucine levels	16	4.22E-71	0.049767264	1.05 (1.00-1.10)	0.340447687	0.17650407	1.05 (0.98-1.12)	-9.15E-05	0.163510864	1.04 (0.98-1.11)	0.166253982	1.04 (0.99-1.10)	0.626060699	1.04 (0.89-1.22)
X-12798 levels	70	0	0.000782819	1.04 (1.02-1.06)	0.226391177	0.09448431	1.03 (0.99-1.07)	0.001387432	0.108570953	1.03 (0.99-1.07)	0.02724676	1.04 (1.01-1.08)	0.099566997	1.06 (0.99-1.12)
Arachidonoylcarnitine (C20:4) levels	79	0	0.004932147	1.04 (1.01-1.06)	0.131427166	0.05679491	1.06 (1.00-1.12)	-0.003047673	0.013361733	1.06 (1.01-1.11)	0.001939273	1.07 (1.03-1.12)	0.623846318	1.02 (0.93-1.12)
X-21355 levels	15	7.00E-66	0.010692003	1.04 (1.01-1.07)	0.497572307	0.03887405	1.05 (1.01-1.09)	-0.00372872	0.057413099	1.04 (1.00-1.08)	0.04490526	1.04 (1.00-1.08)	0.048079343	1.14 (1.01-1.28)
1-linoleoyl-2-arachidonoyl-GPC (18:2/20:4n6) levels	37	0	0.018663238	1.04 (1.01-1.08)	0.446549363	0.12384457	1.06 (0.99-1.14)	-0.00257535	0.026255021	1.06 (1.01-1.12)	0.010734741	1.09 (1.02-1.17)	0.227505549	1.08 (0.96-1.21)
Carnitine C18:2 levels	40	5.05E-307	0.033500099	1.04 (1.00-1.07)	0.736438991	0.47192175	1.02 (0.97-1.07)	0.003725818	0.538330868	1.02 (0.96-1.07)	0.382394629	1.02 (0.98-1.07)	0.726715818	1.02 (0.92-1.13)
Urea levels	23	2.42E-103	0.034287801	1.04 (1.00-1.08)	0.036319058	0.12115297	1.04 (0.99-1.08)	0.002010597	0.048038452	1.04 (1.00-1.08)	0.028530054	1.04 (1.01-1.08)	0.371431852	1.08 (0.91-1.29)
Lignoceroylcarnitine (C24) levels	33	1.28E-291	0.038807346	1.04 (1.00-1.08)	0.939352548	0.12421528	1.07 (0.98-1.17)	-0.00509444	0.105464414	1.05 (0.99-1.11)	0.100501657	1.05 (0.99-1.12)	0.514191728	1.03 (0.94-1.13)
X-12830 levels	25	4.94E-118	0.049087286	1.04 (1.00-1.09)	0.555675832	0.61957072	1.02 (0.95-1.09)	0.006005325	0.348437832	1.03 (0.97-1.10)	0.291361653	1.04 (0.97-1.11)	0.15908977	1.08 (0.97-1.19)
5-acetylamino-6-formylamino-3-methyluracil levels	50	0	0.010430171	1.03 (1.01-1.05)	0.367749918	0.18405603	1.03 (0.99-1.07)	0.000832487	0.693151399	1.01 (0.97-1.04)	0.392970698	1.01 (0.98-1.04)	0.871550596	1.00 (0.95-1.07)
Biliverdin levels	49	0	0.023535256	1.03 (1.00-1.06)	0.065370515	0.14350161	1.04 (0.99-1.10)	-0.001951385	0.020187644	1.05 (1.01-1.10)	0.09666832	1.04 (0.99-1.08)	0.415214388	1.03 (0.96-1.10)
Dodecadienoate (12:2) levels	29	2.62E-192	0.033686946	1.03 (1.00-1.05)	0.839577454	0.18178779	1.02 (0.99-1.05)	0.003705783	0.208646904	1.02 (0.99-1.05)	0.203409235	1.02 (0.99-1.05)	0.577778532	1.04 (0.92-1.17)
4-guanidinobutanoate levels	48	0	0.036852973	1.03 (1.00-1.06)	0.483583911	0.85915301	0.99 (0.94-1.05)	0.007822391	0.339656816	1.02 (0.98-1.07)	0.27324168	1.03 (0.98-1.08)	0.021648267	1.13 (1.02-1.25)
1-(1-enyl-palmitoyl)-2-arachidonoyl-gpc (p-16:0/20:4) levels	59	0	0.043542321	1.03 (1.00-1.06)	0.019716711	0.24863279	1.03 (0.98-1.09)	-0.000247462	0.005966439	1.06 (1.02-1.10)	0.014618317	1.06 (1.01-1.11)	0.176846695	1.07 (0.97-1.18)
X-12112 levels	98	0	0.018807304	0.98 (0.96-1.00)	0.298169758	0.28755117	0.99 (0.96-1.01)	-0.001583477	0.022778474	0.97 (0.94-1.00)	0.04164973	0.97 (0.95-1.00)	0.049080163	0.94 (0.89-1.00)
Paraxanthine to 5-acetylamino-6-formylamino-3-methyluracil ratio	64	0	0.001443961	0.97 (0.96-0.99)	0.196852601	0.3040548	0.99 (0.96-1.01)	-0.004458601	0.630207664	0.99 (0.97-1.02)	0.207256022	0.99 (0.97-1.01)	0.38604619	0.98 (0.94-1.02)
Butyrylglycine levels	47	0	0.010470777	0.97 (0.95-0.99)	0.567934722	0.19084889	0.97 (0.93-1.01)	-7.54E-05	0.017692661	0.96 (0.92-0.99)	0.059642539	0.97 (0.94-1.00)	0.490409632	0.98 (0.91-1.04)
Decadienedioic acid (C10:2-DC) levels	67	0	0.015890023	0.97 (0.95-0.99)	0.022921398	0.01126414	0.95 (0.91-0.99)	0.007411034	0.007209384	0.96 (0.93-0.99)	0.005396015	0.96 (0.93-0.99)	0.686462813	1.01 (0.95-1.09)
Ferulic acid 4-sulfate levels	36	1.32E-268	0.022232129	0.97 (0.94-1.00)	0.00012046	0.24710888	0.98 (0.95-1.01)	-0.007993321	0.089712126	0.98 (0.95-1.00)	0.041665318	0.98 (0.95-1.00)	0.559006708	0.97 (0.87-1.08)
1-palmitoyl-2-linoleoyl-GPE (16:0/18:2) levels	53	0	0.032075471	0.97 (0.95-1.00)	0.154094483	0.66150224	0.99 (0.94-1.04)	-0.003567793	0.090223445	0.96 (0.92-1.01)	0.023855795	0.95 (0.92-0.99)	0.23660374	0.95 (0.88-1.03)
Glycine to alanine ratio	36	0	0.042065446	0.97 (0.93-1.00)	0.229696972	0.76379743	0.99 (0.93-1.06)	-0.00548941	0.450434583	0.98 (0.94-1.03)	0.361118018	0.98 (0.93-1.03)	0.040684157	0.90 (0.81-0.99)
Glutarylcarnitine (c5-dc) levels	66	0	0.044977404	0.97 (0.94-1.00)	0.186904331	0.70562137	0.99 (0.92-1.06)	-0.002579966	0.36745819	0.98 (0.93-1.03)	0.277215214	0.97 (0.93-1.02)	0.373817764	0.96 (0.89-1.05)
5-hydroxylysine levels	47	0	0.045660448	0.97 (0.94-1.00)	0.088885984	0.64757895	1.01 (0.95-1.08)	-0.010499886	0.128641711	0.96 (0.91-1.01)	0.312605369	0.97 (0.93-1.02)	0.427143953	0.96 (0.87-1.06)
N-acetyl-aspartyl-glutamate (naag) levels	139	NA	4.86E-10	0.96 (0.94-0.97)	8.31E-08	0.14948158	0.98 (0.96-1.01)	-0.01091556	0.001488755	0.97 (0.95-0.99)	3.94E-06	0.96 (0.95-0.98)	7.79E-06	0.90 (0.86-0.94)
Metabolonic lactone sulfate levels	72	0	0.000312511	0.96 (0.94-0.98)	0.13333082	0.17315615	0.98 (0.94-1.01)	-0.00381813	0.018978158	0.96 (0.93-0.99)	0.048998199	0.97 (0.93-1.00)	0.470250337	0.98 (0.92-1.04)
X-26109 levels	51	0	0.000640394	0.96 (0.94-0.98)	0.411478129	0.012765	0.95 (0.92-0.99)	0.002125517	0.027137351	0.96 (0.92-1.00)	0.047811174	0.96 (0.93-1.00)	0.519574706	0.98 (0.92-1.04)
Glucuronide of piperine metabolite C17H21NO3 (3) levels	16	2.63E-89	0.003680059	0.96 (0.93-0.99)	0.24944087	0.00639907	0.94 (0.90-0.98)	0.0094664	0.079391529	0.96 (0.91-1.01)	0.006900466	0.94 (0.90-0.98)	0.591756658	1.03 (0.92-1.17)
Octanoylcarnitine (c8) levels	48	0	0.02257177	0.96 (0.93-0.99)	0.392107956	0.90420457	1.00 (0.94-1.06)	-0.006835315	0.830455327	0.99 (0.94-1.05)	0.900261467	1.00 (0.94-1.05)	0.57222584	0.97 (0.87-1.08)
Tryptophan betaine levels	44	2.40E-294	0.030638757	0.96 (0.93-1.00)	0.397938989	0.47834568	0.98 (0.91-1.04)	-0.002812044	0.083483807	0.95 (0.89-1.01)	0.225724677	0.95 (0.87-1.03)	0.566510037	0.97 (0.86-1.09)
Succinylcarnitine levels	45	0	0.032444349	0.96 (0.93-1.00)	0.228155613	0.05657361	0.93 (0.87-1.00)	0.006451712	0.00189972	0.92 (0.87-0.97)	0.006341145	0.92 (0.87-0.97)	0.184473577	0.93 (0.85-1.03)
Glucuronide of piperine metabolite C17H21NO3 (4) levels	24	1.74E-139	0.049041642	0.96 (0.92-1.00)	0.01301292	0.00420483	0.93 (0.89-0.97)	0.015030304	0.00967825	0.93 (0.89-0.98)	0.017404356	0.94 (0.90-0.99)	0.579128632	0.96 (0.85-1.09)
2-hydroxy-4-(methylthio)butanoic acid levels	48	0	0.011418379	0.95 (0.92-0.99)	0.066357907	0.12240995	0.93 (0.85-1.02)	0.004274442	0.648504835	0.99 (0.93-1.04)	0.801559502	1.01 (0.93-1.10)	0.727062311	1.02 (0.90-1.16)
Serine to threonine ratio	33	0	0.014710949	0.95 (0.91-0.99)	0.555775629	0.40557081	0.96 (0.88-1.05)	-0.001791696	0.339738624	0.97 (0.91-1.03)	0.327328809	0.97 (0.91-1.03)	0.654378783	0.97 (0.87-1.09)
Hexanoylglutamine levels	47	0	0.016404343	0.95 (0.91-0.99)	0.031124827	0.4558375	1.03 (0.95-1.13)	-0.015063758	0.08510011	0.95 (0.90-1.01)	0.206990672	0.96 (0.91-1.02)	0.275921841	0.93 (0.83-1.05)
1-stearoyl-2-linoleoyl-GPI (18:0/18:2) levels	35	3.96E-275	0.023426851	0.95 (0.91-0.99)	0.481626073	0.24008237	1.07 (0.96-1.19)	-0.015302525	0.107495126	0.94 (0.88-1.01)	0.059531637	0.87 (0.76-1.00)	0.069522796	0.87 (0.76-1.01)
Dihydrocaffeate sulfate (2) levels	29	6.23E-132	0.027576683	0.95 (0.90-0.99)	0.427766868	0.24130271	0.94 (0.84-1.04)	0.001624177	0.090817366	0.94 (0.87-1.01)	0.265501889	0.95 (0.87-1.04)	0.442078051	0.95 (0.85-1.07)
Allantoin levels	24	7.94E-108	0.031652025	0.95 (0.91-1.00)	0.268407661	0.92982924	1.00 (0.93-1.07)	-0.010576783	0.469179605	0.97 (0.90-1.05)	0.567703842	0.98 (0.93-1.04)	0.943173713	1.00 (0.89-1.11)
Hydroxypalmitoyl sphingomyelin (d18:1/16:0(OH)) levels	38	9.62E-273	0.037107124	0.95 (0.90-1.00)	0.024624417	0.80647732	0.99 (0.88-1.10)	-0.006196885	0.001758691	0.90 (0.84-0.96)	0.00789483	0.90 (0.83-0.97)	0.276422035	0.92 (0.79-1.07)
Phosphate to urate ratio	43	1.54E-282	0.037974288	0.95 (0.91-1.00)	0.395966726	0.67507962	0.98 (0.87-1.09)	-0.003093786	0.30442295	0.96 (0.89-1.04)	0.671904022	0.97 (0.87-1.10)	0.751078656	1.03 (0.87-1.22)
Adenosine 5'-diphosphate (ADP) to glycerol ratio	20	5.40E-95	0.040483358	0.95 (0.91-1.00)	0.153919919	0.13623166	1.06 (0.98-1.15)	-0.024539671	0.912035669	1.00 (0.93-1.07)	0.757383801	1.01 (0.94-1.08)	0.600086664	0.97 (0.86-1.09)
4-cholesten-3-one levels	21	4.78E-103	0.046256569	0.95 (0.90-1.00)	0.140271668	0.53951157	0.96 (0.84-1.10)	-0.001750173	0.162940947	0.94 (0.86-1.02)	0.224416389	0.93 (0.83-1.04)	0.282632974	0.93 (0.81-1.06)
N-formylanthranilic acid levels	39	0	2.98E-05	0.94 (0.91-0.97)	0.151005349	0.04005103	0.94 (0.88-0.99)	-0.00040881	0.062437464	0.95 (0.90-1.00)	0.066536472	0.95 (0.91-1.00)	0.199478924	0.94 (0.85-1.03)
Propionylglycine levels	25	1.77E-248	0.001519743	0.94 (0.90-0.98)	0.148060477	0.9758278	1.00 (0.91-1.09)	-0.011956918	0.43362501	0.98 (0.92-1.03)	0.310850672	0.97 (0.91-1.03)	0.14531254	0.92 (0.83-1.03)
Cysteinylglycine disulfide levels	42	0	0.001852618	0.94 (0.90-0.98)	0.025587881	0.46762428	1.03 (0.95-1.13)	-0.017758146	0.04839693	0.94 (0.89-1.00)	0.133073905	0.95 (0.89-1.01)	0.074140852	0.91 (0.83-1.01)
X-24306 levels	25	2.77E-114	0.003578389	0.94 (0.90-0.98)	0.487226103	0.00786193	0.92 (0.86-0.97)	0.00653271	0.125405725	0.95 (0.90-1.01)	0.090163472	0.95 (0.89-1.01)	0.617250534	0.97 (0.87-1.08)
S-1-pyrroline-5-carboxylate levels	34	1.42E-265	0.007658483	0.94 (0.91-0.98)	0.470494699	0.99644926	1.00 (0.91-1.10)	-0.00977694	0.136314308	0.95 (0.89-1.02)	0.461052953	0.97 (0.88-1.06)	0.28809537	0.93 (0.83-1.06)
N-acetylglucosamine/n-acetylgalactosamine levels	23	4.27E-185	0.011634823	0.94 (0.89-0.99)	0.252960116	0.35086794	0.95 (0.86-1.06)	-0.002168128	0.184269461	0.95 (0.89-1.02)	0.229393972	0.95 (0.89-1.03)	0.69809065	0.98 (0.87-1.10)
X-12127 levels	34	1.32E-165	0.017490979	0.94 (0.90-0.99)	0.180633471	0.27155137	0.92 (0.79-1.07)	0.003554426	0.391886534	0.97 (0.90-1.04)	0.422017727	1.07 (0.91-1.26)	0.398267684	1.08 (0.91-1.29)
N-acetylalliin levels	25	3.01E-118	0.019223452	0.94 (0.90-0.99)	0.152466336	0.53795944	0.96 (0.84-1.10)	-0.002245625	0.086671971	0.94 (0.88-1.01)	0.287292752	0.94 (0.84-1.05)	0.367363831	0.94 (0.83-1.07)
Cysteinylglycine to taurine ratio	33	1.99E-181	0.019488332	0.94 (0.90-0.99)	0.130943122	0.44601936	0.95 (0.85-1.07)	-0.001872543	0.279632604	0.96 (0.88-1.04)	0.29780943	0.95 (0.86-1.04)	0.59019946	0.96 (0.82-1.12)
Alpha-hydroxycaproate levels	16	1.06E-74	0.023914666	0.94 (0.90-0.99)	0.7915988	0.50584779	0.96 (0.87-1.07)	-0.005780136	0.113254527	0.94 (0.88-1.01)	0.143243904	0.94 (0.87-1.02)	0.222384932	0.94 (0.84-1.04)
X-11372 levels	27	1.80E-144	0.025472028	0.94 (0.89-0.99)	0.21834409	0.07773738	0.89 (0.78-1.01)	0.007887483	0.088458725	0.93 (0.86-1.01)	0.092739465	0.92 (0.85-1.01)	0.230568751	0.91 (0.78-1.06)
Carotene diol (2) levels	23	2.83E-151	0.027497821	0.94 (0.88-0.99)	0.115732755	0.96786756	1.00 (0.84-1.20)	-0.009986764	0.121497908	0.93 (0.85-1.02)	0.142032486	0.93 (0.86-1.02)	0.22597428	0.92 (0.80-1.05)
Glucuronide of C10H18O2 (7) levels	21	5.27E-100	0.032621352	0.94 (0.88-0.99)	0.053251352	0.16192957	0.89 (0.76-1.04)	0.008074255	0.116144645	0.93 (0.85-1.02)	0.262311612	0.92 (0.80-1.06)	0.254718714	0.91 (0.77-1.07)
Uridine to 2'-deoxyuridine ratio	26	1.29E-139	0.035925241	0.94 (0.89-1.00)	0.251083808	0.7431128	0.97 (0.83-1.14)	-0.004904352	0.230378971	0.94 (0.86-1.04)	0.47869909	0.95 (0.84-1.08)	0.083064356	0.84 (0.70-1.01)
1-stearoyl-2-linoleoyl-gpc (18:0/18:2) levels	25	1.71E-170	0.037269404	0.94 (0.89-1.00)	0.286278772	0.20651253	0.91 (0.80-1.05)	0.004237669	0.011131551	0.90 (0.82-0.98)	0.016906431	0.89 (0.81-0.97)	0.934895358	0.99 (0.85-1.16)
Hypotaurine to cysteine ratio	31	1.91E-159	0.04157524	0.94 (0.89-1.00)	0.242227094	0.00173356	0.75 (0.64-0.89)	0.026057441	0.156593462	0.94 (0.87-1.02)	0.216023662	0.92 (0.81-1.05)	0.257531195	0.91 (0.77-1.07)
X-12849 levels	24	5.03E-107	0.046240936	0.94 (0.89-1.00)	0.390585228	0.40725485	0.95 (0.83-1.08)	-0.000641035	0.099031701	0.93 (0.85-1.01)	0.383178841	0.95 (0.84-1.07)	0.14622334	0.89 (0.77-1.04)
Trans-2-hexenoylglycine levels	28	5.74E-175	0.047273515	0.94 (0.89-1.00)	0.045353787	0.17003802	0.91 (0.80-1.04)	0.005244757	0.310308316	0.96 (0.89-1.04)	0.314708746	0.95 (0.87-1.04)	0.236686866	0.91 (0.79-1.06)
N-acetylvaline levels	19	1.42E-88	0.047748093	0.94 (0.88-1.00)	0.332229924	0.13720642	0.91 (0.82-1.02)	0.004570911	0.231762107	0.94 (0.86-1.04)	0.205322888	0.93 (0.83-1.04)	0.301379864	0.92 (0.79-1.07)
4-allylphenol sulfate levels	23	3.96E-108	0.049528435	0.94 (0.89-1.00)	0.074044804	0.32096576	0.93 (0.80-1.07)	0.002589858	0.131857173	0.94 (0.86-1.02)	0.268266799	0.92 (0.81-1.06)	0.142298438	0.89 (0.76-1.03)
Mannose to glycerol ratio	19	1.11E-112	0.010224556	0.93 (0.88-0.98)	0.440840064	0.72681791	0.98 (0.89-1.08)	-0.009494106	0.375747328	0.96 (0.89-1.05)	0.462078493	0.97 (0.90-1.05)	0.687225562	0.97 (0.84-1.12)
X-11880 levels	17	6.70E-114	0.022598127	0.93 (0.87-0.99)	0.689016435	0.44805078	0.94 (0.80-1.10)	-0.002051326	0.081211365	0.92 (0.84-1.01)	0.159195944	0.91 (0.81-1.03)	0.151173896	0.89 (0.77-1.03)
2-linoleoylglycerol (18:2) levels	21	1.26E-92	0.023834987	0.93 (0.87-0.99)	0.177861099	0.51557137	1.06 (0.90-1.25)	-0.020040186	0.264331566	0.95 (0.87-1.04)	0.436372838	0.94 (0.82-1.09)	0.458886994	0.94 (0.79-1.11)
1-palmitoyl-GPG (16:0) levels	28	1.01E-163	0.024079526	0.93 (0.87-0.99)	0.02892618	0.08707278	0.87 (0.75-1.01)	0.008542195	0.160222869	0.94 (0.86-1.02)	0.123245355	0.94 (0.86-1.02)	0.31332491	0.93 (0.82-1.06)
5-hydroxyindole sulfate levels	20	1.86E-99	0.026157506	0.93 (0.87-0.99)	0.225651456	0.25404085	0.90 (0.75-1.08)	0.004594872	0.799098553	0.99 (0.89-1.09)	0.875129737	1.01 (0.85-1.22)	0.855355026	1.02 (0.83-1.25)
Thymol sulfate levels	20	1.54E-101	0.031245744	0.93 (0.87-0.99)	0.629056782	0.12783066	0.88 (0.74-1.03)	0.008104164	0.196545346	0.94 (0.86-1.03)	0.456215308	0.94 (0.81-1.10)	0.763285242	0.97 (0.83-1.15)
Benzoate to linoleoyl-arachidonoyl-glycerol (18:2 to 20:4) [1] ratio	20	1.56E-157	0.039559709	0.93 (0.86-1.00)	0.002405842	0.84339223	0.98 (0.81-1.18)	-0.009357237	0.005324206	0.89 (0.82-0.97)	0.065048281	0.91 (0.82-1.00)	0.859518544	0.98 (0.82-1.18)
Dihomo-linolenate (20:3n3 or n6) levels	21	8.50E-106	0.040821831	0.93 (0.88-1.00)	0.135191401	0.84511239	0.98 (0.83-1.17)	-0.007241882	0.243866473	0.95 (0.86-1.04)	0.427583259	0.94 (0.81-1.09)	0.273806421	0.90 (0.76-1.08)
X-21821 levels	18	7.30E-83	0.044628254	0.93 (0.87-1.00)	0.937492394	0.21133646	0.91 (0.80-1.05)	0.002891701	0.475698827	0.97 (0.88-1.06)	0.639530088	0.97 (0.84-1.12)	0.7327408	0.97 (0.83-1.14)
Cysteinylglycine levels	23	9.75E-207	0.004126105	0.92 (0.86-0.97)	0.035737855	0.09092865	0.87 (0.75-1.01)	0.00741161	0.009158041	0.91 (0.84-0.98)	0.018044382	0.90 (0.83-0.98)	0.308878263	0.94 (0.83-1.06)
X-17690 levels	35	2.02E-204	0.007043086	0.92 (0.87-0.98)	0.009052862	0.73408992	0.97 (0.84-1.13)	-0.00768673	0.056085374	0.94 (0.87-1.00)	0.219693624	0.93 (0.83-1.04)	0.170396975	0.91 (0.80-1.04)

4-hydroxy-2-oxoglutaric acid levels	27	1.83E-124	0.010439288	0.92 (0.87-0.98)	0.116440351	0.61729687	0.96 (0.83-1.12)	-0.005926354	0.011066858	0.89 (0.81-0.97)	0.125382165	0.88 (0.76-1.03)	0.123111957	0.87 (0.73-1.03)
Alpha-ketobutyrate to 3-methyl-2-oxobutyrate ratio	22	2.68E-105	0.011433513	0.92 (0.86-0.98)	0.689777959	0.65203693	0.96 (0.82-1.13)	-0.006306075	0.172492635	0.94 (0.86-1.03)	0.511534431	0.95 (0.83-1.10)	0.39779371	0.93 (0.79-1.10)
Taurine to cysteine ratio	20	2.55E-87	0.029916596	0.92 (0.85-0.99)	0.266212482	0.79441059	1.02 (0.87-1.21)	-0.013497362	0.159780414	0.92 (0.82-1.03)	0.238837281	0.91 (0.77-1.06)	0.283601782	0.89 (0.73-1.09)
1-palmitoyl-2-dihomo-linolenoyl-GPC (16:0/20:3n3 or 6) levels	34	8.24E-304	0.000344625	0.91 (0.87-0.96)	0.043622327	0.00491504	0.86 (0.77-0.95)	0.010653368	0.002373261	0.91 (0.85-0.97)	0.005554462	0.90 (0.85-0.97)	0.109133515	0.90 (0.79-1.02)
Stachydrine levels	21	2.04E-91	0.013127481	0.91 (0.84-0.98)	0.14504786	0.06284841	0.83 (0.70-1.00)	0.010908343	0.00571609	0.85 (0.76-0.96)	0.066257299	0.86 (0.73-1.00)	0.126534948	0.85 (0.70-1.04)
4-methyl-2-oxopentanoate to 3-methyl-2-oxobutyrate ratio	20	8.56E-100	0.020513189	0.91 (0.84-0.99)	0.635316567	0.85683445	1.02 (0.86-1.21)	-0.014777331	0.194094648	0.92 (0.82-1.04)	0.364585897	0.93 (0.80-1.08)	0.174550629	0.88 (0.73-1.05)
Phosphate to N-palmitoyl-sphingosine (d18:1 to 16:0) ratio	25	7.30E-110	0.024474388	0.91 (0.83-0.99)	0.002216864	0.51891101	0.94 (0.78-1.13)	-0.004650119	0.02855073	0.90 (0.82-0.99)	0.56034718	1.06 (0.87-1.29)	0.63663714	1.06 (0.82-1.37)
X-12680 levels	15	3.21E-70	0.028236688	0.91 (0.84-0.99)	0.69536518	0.06458811	0.74 (0.55-0.99)	0.02125787	0.153768408	0.92 (0.83-1.03)	0.429679319	0.93 (0.79-1.10)	0.609239578	0.95 (0.80-1.14)
1-linoleoylglycerol (18:2) levels	17	7.43E-75	0.041707364	0.91 (0.84-1.00)	0.670164823	0.0411567	0.79 (0.64-0.97)	0.018407094	0.127997194	0.91 (0.80-1.03)	0.407711856	0.91 (0.74-1.12)	0.384804436	0.91 (0.74-1.12)
Docosatrienoate (22:3n3) levels	27	7.34E-180	2.92E-06	0.90 (0.86-0.94)	0.640302876	0.02051593	0.89 (0.82-0.98)	0.000541206	0.021405299	0.92 (0.85-0.99)	0.215106956	0.93 (0.84-1.04)	0.236269893	0.92 (0.81-1.05)
X-11632 levels	22	1.04E-104	0.00147404	0.90 (0.85-0.96)	0.11027261	0.08019051	0.85 (0.72-1.01)	0.007730185	0.010739929	0.88 (0.80-0.97)	0.026901205	0.80 (0.66-0.96)	0.032733341	0.80 (0.66-0.97)
X-25790 levels	14	2.25E-74	0.022934424	0.90 (0.82-0.99)	0.944252134	0.27684633	0.85 (0.64-1.13)	0.006968018	0.146481039	0.91 (0.81-1.03)	0.305337279	0.92 (0.78-1.08)	0.961302638	1.00 (0.83-1.21)
3-hydroxyhexanoate levels	20	2.81E-104	0.001825751	0.89 (0.83-0.96)	0.051097923	0.49622457	1.07 (0.89-1.29)	-0.024315503	0.062778958	0.91 (0.82-1.01)	0.194705442	0.89 (0.75-1.06)	0.106114159	0.85 (0.70-1.03)
