# Supplementary material for: Global burden and genetic insights of RA and JIA in ages 0–19 years: GBD 2021 and MR analysis
Source: Front Immunol. 2026 Jan 14;16:1661461. doi: 10.3389/fimmu.2025.1661461 (PMC12847325; doi:10.3389/fimmu.2025.1661461)
Supplement: Supplementary file 5 [file DataSheet5.pdf]

Table S4

Exposure	N SNPs	Steiger P-value	Inverse variance weighted			MR Egger			Weighted median		Weighted mode		Simple mode	
			P-value	OR (95%CI)	Heterogeneity P-value	P-value	OR (95%CI)	Egger intercept P-value	P-value	OR (95%CI)	P-value	OR (95%CI)	P-value	OR (95%CI)
**Immune cells**														
TD CD4+ %T cell	3	9.90E-09	0.000198124	2.52 (1.55-4.09)	0.696313213	0.5972419	4.14 (0.09-184.95)	-0.069567133	0.001257407	2.79 (1.49-5.19)	0.11486032	2.95 (1.34-6.50)	0.105161578	2.95 (1.40-6.23)
CD20 on IgD- CD38dim	3	9.39E-10	0.006019297	2.01 (1.22-3.31)	0.389212146	0.4873315	0.06 (0.00-12.17)	0.476538268	0.012217236	2.35 (1.21-4.60)	0.177668407	2.53 (1.04-6.15)	0.172998732	2.53 (1.06-6.08)
HLA DR++ monocyte AC	5	2.05E-17	0.041068679	1.96 (1.03-3.76)	0.033726465	0.4324164	5.75 (0.13-254.30)	-0.172192496	0.015324997	2.15 (1.16-3.98)	0.142684669	2.42 (0.93-6.27)	0.110351317	2.77 (1.04-7.38)
SSC-A on T cell	3	9.47E-11	0.044021531	1.93 (1.02-3.67)	0.163570678	0.3257475	541.81 (0.53-553269.15)	-0.758025207	0.052865077	2.29 (0.99-5.29)	0.967555766	0.98 (0.37-2.61)	0.271494423	3.10 (0.71-13.54)
CD25 on IgD+ CD38br	4	3.35E-16	0.015275393	1.72 (1.11-2.65)	0.083367362	0.4618943	4.32 (0.18-103.25)	-0.14638789	0.173745506	1.54 (0.83-2.88)	0.91014173	1.07 (0.35-3.29)	0.820753239	1.14 (0.39-3.34)
IgD on IgD+ CD38- unsw mem	4	5.92E-16	0.001842417	1.67 (1.21-2.31)	0.088955133	0.9484252	0.84 (0.01-98.34)	0.139730788	0.229445776	1.30 (0.85-1.98)	0.2512608	1.33 (0.90-1.96)	0.354443271	1.37 (0.78-2.41)
CD25 on IgD- CD38-	5	2.82E-51	7.33E-05	1.64 (1.29-2.10)	0.359812457	0.8947049	0.93 (0.37-2.36)	0.147404732	0.000301438	1.82 (1.32-2.52)	0.024670995	1.88 (1.32-2.66)	0.073355519	1.88 (1.13-3.13)
CD28- CD8br AC	4	4.25E-17	0.019168037	1.61 (1.08-2.40)	0.821185902	0.9682051	1.07 (0.05-22.78)	0.067098501	0.039654188	1.62 (1.02-2.55)	0.188625784	1.62 (0.93-2.82)	0.192762881	1.73 (0.91-3.29)
CD25 on sw mem	4	2.31E-69	7.39E-05	1.55 (1.25-1.93)	0.259129916	0.7176306	0.85 (0.40-1.83)	0.193830116	0.000161098	1.66 (1.27-2.16)	0.040019026	1.71 (1.26-2.31)	0.088575407	1.72 (1.12-2.62)
HLA DR++ monocyte %monocyte	7	6.03E-30	0.016217369	1.43 (1.07-1.91)	0.14459577	0.900412	1.08 (0.34-3.44)	0.057727316	0.150062726	1.33 (0.90-1.95)	0.286129641	1.34 (0.82-2.20)	0.280071598	1.38 (0.81-2.35)
CD25 on unsw mem	6	3.37E-89	0.038774042	1.25 (1.01-1.54)	0.116070663	0.6837729	1.29 (0.42-3.97)	-0.007939731	0.068794182	1.29 (0.98-1.69)	0.183775083	1.32 (0.93-1.88)	0.356053701	1.28 (0.80-2.04)
CD27 on unsw mem	10	4.13E-126	0.017892084	1.22 (1.04-1.45)	0.895642842	0.3381652	1.25 (0.82-1.90)	-0.005060304	0.108198793	1.20 (0.96-1.49)	0.157762107	1.20 (0.95-1.51)	0.346887326	1.18 (0.85-1.63)
CD25 on memory B cell	9	7.44E-154	0.030675414	1.21 (1.02-1.43)	0.926219704	0.6931604	1.11 (0.68-1.82)	0.025272433	0.218596051	1.15 (0.92-1.43)	0.256488782	1.17 (0.91-1.50)	0.523724066	1.11 (0.81-1.53)
CD25 on IgD- CD38dim	8	1.64E-134	0.045525565	1.21 (1.00-1.46)	0.932839773	0.7959983	1.07 (0.64-1.81)	0.033838049	0.268266963	1.14 (0.90-1.45)	0.539514265	1.10 (0.82-1.49)	0.6509491	1.08 (0.79-1.48)
CD28 on CD28+ CD45RA+ CD8br	5	9.21E-97	0.049121033	1.19 (1.00-1.41)	0.582887723	0.2675859	1.42 (0.85-2.37)	-0.076140523	0.017443591	1.26 (1.04-1.52)	0.088916129	1.26 (1.03-1.53)	0.231538014	1.23 (0.92-1.64)
CD45RA+ CD28- CD8br AC	3	3.37E-15	0.025637324	1.00 (1.00-1.00)	0.21538554	0.943972	1.00 (0.99-1.01)	0.210811991	0.103963804	1.00 (1.00-1.00)	0.370042497	1.00 (1.00-1.00)	0.365782964	1.00 (1.00-1.00)
HLA DR on DC	16	7.20E-187	0.033680265	0.80 (0.66-0.98)	0.000419726	0.9258351	0.96 (0.45-2.06)	-0.058090982	0.004681667	0.77 (0.64-0.92)	0.034744707	0.78 (0.63-0.96)	0.079733753	0.78 (0.61-1.01)
CD25hi %CD4+	6	1.22E-40	0.026342944	0.75 (0.58-0.97)	0.060410809	0.0726623	0.48 (0.26-0.87)	0.131592684	0.015566105	0.65 (0.46-0.92)	0.024461688	0.56 (0.40-0.80)	0.524525365	1.35 (0.57-3.19)
HLA DR on CD14- CD16+ monocyte	16	1.13E-81	0.024154758	0.74 (0.57-0.96)	0.002100565	0.7664277	0.87 (0.35-2.16)	-0.032458511	0.008305728	0.71 (0.55-0.92)	0.023707383	0.67 (0.49-0.91)	0.046799547	0.54 (0.31-0.94)
CD25hi %T cell	5	4.83E-29	0.029097236	0.72 (0.54-0.97)	0.056903233	0.0468366	0.39 (0.23-0.69)	0.157796018	0.188820347	0.75 (0.49-1.15)	0.031322789	0.50 (0.33-0.76)	0.723033333	1.18 (0.51-2.70)
CD8 on CD28+ CD45RA- CD8br	7	3.92E-25	0.028388534	0.71 (0.53-0.96)	0.057072724	0.3644247	0.19 (0.01-4.97)	0.226218878	0.088425449	0.66 (0.41-1.06)	0.169005257	0.64 (0.36-1.12)	0.511090932	1.32 (0.60-2.89)
HLA DR on myeloid DC	13	8.01E-134	0.012247202	0.69 (0.52-0.92)	7.57E-06	0.4434254	0.71 (0.30-1.67)	-0.006836704	0.026968819	0.76 (0.59-0.97)	0.026947848	0.71 (0.55-0.93)	0.010120159	0.53 (0.35-0.80)
Secreting Treg %CD4	5	9.50E-28	0.006402534	0.64 (0.46-0.88)	0.192753255	0.2539589	0.32 (0.06-1.57)	0.138972032	0.028761609	0.62 (0.40-0.95)	0.105912337	0.57 (0.34-0.97)	0.822840354	0.92 (0.48-1.79)
CD8 on CD39+ CD8br	5	4.02E-25	0.015578664	0.64 (0.44-0.92)	0.450740342	0.5799628	2.30 (0.16-32.27)	-0.219954634	0.059445203	0.66 (0.42-1.02)	0.238436017	0.64 (0.35-1.20)	0.333518767	0.70 (0.37-1.32)
CD25hi CD45RA- CD4 not Treg %CD4+	3	3.32E-25	0.001612228	0.63 (0.48-0.84)	0.158570699	0.2320038	0.49 (0.29-0.83)	0.083943186	0.001471839	0.58 (0.42-0.81)	0.070296577	0.56 (0.41-0.77)	0.242986187	0.60 (0.33-1.10)
CD20 on unsw mem	3	1.07E-09	0.03822508	0.56 (0.33-0.97)	0.092681506	0.539066	16.94 (0.03-8972.91)	-0.474597763	0.25241992	0.62 (0.28-1.40)	0.623130384	0.72 (0.23-2.22)	0.446994754	0.60 (0.20-1.76)
CD45RA- CD4+ %T cell	3	9.64E-11	0.000619263	0.52 (0.35-0.75)	0.254616618	0.3132031	0.01 (0.00-1.29)	0.709200023	0.069077473	0.59 (0.34-1.04)	0.31498785	0.65 (0.34-1.23)	0.329845112	0.65 (0.33-1.26)
CD4 on CD39+ CD4+	4	4.97E-15	0.000657573	0.51 (0.34-0.75)	0.265712249	0.4707108	3.10 (0.25-38.08)	-0.318369987	0.023736246	0.54 (0.32-0.92)	0.140243558	0.55 (0.31- 0.99)	0.283586608	0.62 (0.31-1.27)
CD4 on secreting Treg	3	1.63E-10	0.004100741	0.48 (0.29-0.79)	0.101586242	0.275937	0.16 (0.03-0.84)	0.216166553	0.133894862	0.56 (0.26-1.20)	0.791439545	0.85 (0.29-2.46)	0.887464357	0.91 (0.29-2.83)
CD28 on CD39+ activated Treg	3	9.59E-15	0.003894176	0.46 (0.27-0.78)	0.591434668	0.3802154	0.08 (0.00-2.28)	0.380977025	0.088646975	0.54 (0.27-1.10)	0.27623604	0.55 (0.25-1.21)	0.251275592	0.55 (0.26-1.15)
CD8 on CD28- CD8br	5	8.98E-17	0.000614728	0.44 (0.28-0.71)	0.864980544	0.5626866	0.29 (0.01-12.25)	0.06427334	0.015490158	0.46 (0.24-0.86)	0.093465135	0.47 (0.24- 0.92)	0.111058541	0.46 (0.22-0.97)
CD4 on HLA DR+ CD4+	8	3.95E-27	0.00025553	0.39 (0.23-0.65)	0.017860376	0.3694196	20.66 (0.05-9375.59)	-0.632933105	0.041653365	0.59 (0.35-0.98)	0.069986794	0.59 (0.36-0.96)	0.151708372	0.52 (0.23-1.16)
CD4 on activated Treg	4	5.86E-15	1.53E-06	0.37 (0.25-0.56)	0.235123592	0.3570748	4.04 (0.40-40.40)	-0.399276703	0.000428148	0.37 (0.21-0.64)	0.067816086	0.43 (0.24-0.78)	0.059249385	0.27 (0.11-0.64)
**Inflammatory cytokines**														
TNF-beta levels	3	1.53E-86	4.16E-11	0.42 (0.33-0.54)	0.213560506	0.0304501	0.47 (0.27-0.85)	-0.018313152	2.00E-06	0.45 (0.33-0.63)	0.001347222	0.48 (0.34-0.67)	0.003219177	0.28 (0.14-0.54)
C-X-C motif chemokine 9 levels	4	2.12E-05	2.47E-10	10.97 (5.23- 23.05)	0.990428819	0.4295412	8.69 (0.29-257.73)	0.019745264	1.58E-06	10.84 (4.10- 28.69)	0.050143999	10.75 (3.64- 31.76)	0.057692354	10.84 (3.35- 35.06)
T-cell surface glycoprotein CD5 levels	3	2.94E-09	0.000140156	4.22 (2.01- 8.85)	0.407694952	0.2209695	12.00 (2.06-69.88)	-0.117207351	0.000266524	4.89 (2.08-11.49)	0.069183351	5.09 (2.10-12.35)	0.134610637	5.33 (1.39-20.44)
C-C motif chemokine 4 levels	4	1.20E-34	0.003219076	0.52 (0.34-0.80)	0.761847214	0.2263948	0.35 (0.09-1.47)	0.041088971	0.006616081	0.47 (0.28-0.81)	0.391976986	0.68 (0.31-1.52)	0.412660807	0.70 (0.33-1.52)
Signaling lymphocytic activation molecule levels	6	5.20E-05	0.012359919	7.53 (1.55- 36.60)	0.00691448	0.7333594	0.30 (0.00-129.00)	0.269097534	0.000505478	7.60 (2.42- 23.84)	0.043621395	9.34 (2.54- 34.35)	0.077768316	10.60 (1.83- 61.25)
Fms-related tyrosine kinase 3 ligand levels	12	5.50E-15	0.037994947	2.01 (1.04- 3.88)	0.473193543	0.4919844	0.38 (0.04- 3.72)	0.143051843	0.048355017	2.16 (1.01- 4.62)	0.727127104	1.19 (0.49- 2.93)	0.169341001	3.52 (0.90-13.87)
**Blood metabolites**														
Cholesterol to oleoyl-linoleoyl-glycerol (18:1 to 18:2) [2] ratio	3	1.71E-06	0.028600006	2.55 (1.10-5.91)	0.108623887	0.9261837	7.84 (0.00-8.804622e+15)	-0.083623139	0.090344074	2.78 (0.85-9.07)	0.235391681	3.77 (0.80-1.778000e+01)	0.22535832	4.15 (0.83-2.077000e+01)
Aspartate levels	3	8.28E-17	0.003398062	2.32 (1.32-4.09)	0.393625457	0.6840588	1.88 (0.19-18.38)	0.027130693	0.009137238	2.37 (1.24-4.55)	0.12438078	2.42 (1.23-4.75)	0.161560328	2.82 (1.11- 7.17)
X-11470 levels	8	5.44E-27	0.036291668	0.61 (0.38-0.97)	0.337610752	0.5957678	0.64 (0.14-3.01)	-0.00755335	0.184149941	0.64 (0.33-1.24)	0.39380783	0.67 (0.28-1.60)	0.950172586	1.04 (0.33-3.25)
X-24544 levels	4	1.17E-28	0.021824028	0.57 (0.35-0.92)	0.634967727	0.2685604	0.38 (0.11-1.33)	0.060967283	0.016791477	0.54 (0.32-0.89)	0.152266173	0.56 (0.31-1.02)	0.128967119	0.42 (0.19-0.95)
1-(1-enyl-palmitoyl)-2-oleoyl-GPE (p-16:0/18:1) levels	3	6.73E-08	0.009083466	0.34 (0.15-0.77)	0.8661249	0.9265351	2.45 (0.00-9144802.60)	-0.186210834	0.021069968	0.31 (0.11-0.84)	0.163444255	0.29 (0.09-0.89)	0.167808557	0.29 (0.09-0.91)
Propyl 4-hydroxybenzoate sulfate levels	3	6.56E-06	0.00208217	0.25 (0.10-0.60)	0.63902695	0.6518469	0.42 (0.03-6.87)	-0.076267456	0.030282363	0.28 (0.09-0.89)	0.226349556	0.32 (0.09-1.17)	0.211151686	0.28 (0.07-1.11)
